# Antigens Expressed by Breast Cancer Cells Undergoing EMT Stimulate Cytotoxic CD8^+^ T Cell Immunity

**DOI:** 10.3390/cancers14184397

**Published:** 2022-09-09

**Authors:** Faye A. Camp, Tonya M. Brunetti, Michelle M. Williams, Jessica L. Christenson, Varsha Sreekanth, James C. Costello, Zachary L. Z. Hay, Ross M. Kedl, Jennifer K. Richer, Jill E. Slansky

**Affiliations:** 1 Department of Immunology and Microbiology, University of Colorado School of Medicine, Aurora, CO 80045, USA; 2 Department of Pathology, University of Colorado School of Medicine, Aurora, CO 80045, USA; 3 Department of Pharmacology, University of Colorado School of Medicine, Aurora, CO 80045, USA

**Keywords:** neojunction, neoantigen, intron retention, mIR-200c, epithelial-to-mesenchymal transition (EMT), CD8^+^ T cells, peptide vaccines, whole cell vaccines

## Abstract

**Simple Summary:**

The transition of cells with epithelial characteristics to those with mesenchymal characteristics (termed EMT) facilitates breast cancer invasive capacity. The EMT program can also contribute to immunosuppressive and immunoevasive properties, altering susceptibility to immune cell recognition and killing. The goal of our study was to manipulate EMT to reveal potential neoantigens that might affect the ability of tumor cells to circumvent immune escape and/or be utilized as an anticancer vaccine to kill cancer cells exhibiting the cellular plasticity that permits therapy resistance and metastatic progression. We identified potential neoantigens resulting from EMT-associated altered gene expression and alternative splicing events and observed increased immunogenicity and susceptibility to killing of the more epithelial-like cancer cells. Although the tested peptides did not protect from tumor growth, a limited number of predicted neoantigens derived from intron retention events were tested. In the future, refined prediction programs may facilitate exciting antigen discoveries.

**Abstract:**

Antigenic differences formed by alterations in gene expression and alternative splicing are predicted in breast cancer cells undergoing epithelial to mesenchymal transition (EMT) and the reverse plasticity known as MET. How these antigenic differences impact immune interactions and the degree to which they can be exploited to enhance immune responses against mesenchymal cells is not fully understood. We utilized a master microRNA regulator of EMT to alter mesenchymal-like EO771 mammary carcinoma cells to a more epithelial phenotype. A computational approach was used to identify neoantigens derived from the resultant differentially expressed somatic variants (SNV) and alternative splicing events (neojunctions). Using whole cell vaccines and peptide-based vaccines, we find superior cytotoxicity against the more-epithelial cells and explore the potential of neojunction-derived antigens to elicit T cell responses through experiments designed to validate the computationally predicted neoantigens. Overall, results identify EMT-associated splicing factors common to both mouse and human breast cancer cells as well as immunogenic SNV- and neojunction-derived neoantigens in mammary carcinoma cells.

## 1. Introduction

Metastatic cancer remains the leading cause of all cancer-related deaths [1]. Improved treatments and methods of early detection have led to a dramatic reduction in mortality in women who present with non-metastatic breast cancer, yet we have very limited ways of preventing or combating metastatic disease. Thus, metastatic breast cancer (de novo or recurrent) represents the second leading cause of death in women in the United States and Europe [2,3,4]. Indeed, the 5-year survival rate is 99% for those presenting with localized breast cancer and only 27% for patients with distant-metastatic disease, highlighting the need for new and improved treatment options [5].

One factor in the progression of localized breast cancer to metastatic disease is the activation of a cellular program termed epithelial-to-mesenchymal transition (EMT) [6,7]. As reviewed by Kalluri [8] and Baum et al. [9], EMT occurs during wound healing, tissue fibrosis, cellular migration, and during normal cellular processes throughout embryogenesis. It is proposed that the EMT pathway is hijacked by cancerous cells during metastasis, particularly during invasion and intravasation steps of the metastatic cascade. Broadly, oncogenic EMT confers mesenchymal cellular characteristics, such as anchorage-independent survival, motility, invasive capacity, the plasticity associated with therapeutic resistance, and stem-cell like properties. A major regulator of the epithelial phenotype is the microRNA, miR-200c, often deemed the “guardian of epithelial integrity” [10,11]. Carcinomas can lose miR-200c by microdeletions or silencing due to methylation, and this major determinant of EMT has been extensively used to study properties of breast cancer plasticity and progression [12,13,14,15]. A central pathway in EMT is a reciprocal negative regulatory loop between miR-200c and ZEB1, a repressor of E-cadherin [16].

The adaptive immune system has inherent antitumor capabilities due in part to activation of CD8^+^ T cells following recognition of tumor-derived neoantigens as peptide:MHC class I complexes. This recognition by CD8^+^ T cells triggers a cascade of events that result in tumor-specific cell death. Interestingly, the EMT program promotes several immunosuppressive and immunoevasive properties to the mesenchymal cells. Dongre et al. showed an association between epithelial tumors and mesenchymal tumors and their susceptibility to immune attack, with the epithelial tumors being more susceptible to immunotherapy (anti-CTLA-4) and expressing elevated levels of MHC class I, when compared to mesenchymal counterparts [17]. In addition, miR-200c was found to target a program of genes encoding immune-suppressive factors used to achieve maternal fetal tolerance, but co-opted by carcinoma cells to suppress the immune system to evade immune recognition [13].

The benefits and limitations of targeting cancer cells through neoantigens, particularly SNV (single nucleotide variant)-derived neoantigens, has been known for some time [18]. One study tested the hypothesis that T cells from breast cancer patients could respond to SNV-derived antigens [19]. A total of 2 of the 10 predicted peptides stimulated T cells to secrete IFNγ and were cytotoxic to autologous tumor cells, suggesting the presence of neoantigen-specific T cells, which may be suitable vaccine targets. In another study designed to identify a neoantigen signature for breast cancer, Zhou et al. integrated mutational data from 8 published cohorts of close to 6000 breast cancer patients [20]. Interestingly, they found that four mutations (3 from PIK3CA and 1 from AKT1), predicted to be restricted by HLA alleles common in Han Chinese and Americans, were identified in 2.5–14% of the samples. Thus, a neoantigen from breast cancers can indeed be immunogenic, although the SNVs are rarely shared between patients, requiring each vaccine to be patient specific.

Recently, alternative splicing within cancer cells has emerged as a potential source of neoantigens. By comparing RNA sequencing from tumors in The Cancer Genome Atlas (TCGA) to paired normal tissue and reference tissue from the Genotype-Tissue Expression project (GTEx), Rätsch’s group showed that tumors have 30% more alternative splicing than the respective normal tissues of origin [21,22]. This novel cancer-specific splicing leads to new proteins that may be processed, bind MHC molecules, and presented to T cells as neoantigens. In this analysis, many cancer-type commonalities were observed, suggesting a source of shared antigens not just between patients with the same cancer but across various types of cancer. Evidence of these “neojunctions” has also been identified using mass spectrometry approaches [23]; however, the immunogenicity of these antigens was not confirmed. Furthermore, Shapiro et al. [24] found an EMT-associated alternative splicing program that occurs in human breast cancer cells, resulting in thousands of alterative splicing events regulated by several key splicing factors, such as ESRPs, RBFOX, CELF, and MBNL, suggesting antigen derived from EMT-driven alternative splicing may be shared between cancers.

Using miR-200c restoration in EO771 cells to force MET, we find that CD8^+^ T cells are required in the anticancer immune response against EO771 tumor growth and when miR-200c is restored to render an epithelial-like phenotype (MET), immune cell cytotoxicity was enhanced. EMT associated splicing factors were identified in this mouse mammary carcinoma model and human triple negative breast cancer (TNBC) cells. We surveyed the alternative splicing landscape in EO771 cells with and without miR-200c by bulk RNA sequencing and used computational approaches to conduct an in-depth analysis of intron retention events and resultant neoantigens. Importantly, we developed a novel approach to determine if neojunction-derived antigens elicit T cell responses.

## 2. Materials and Methods

### 2.1. Mice

From Charles River Laboratories, C57BL/6 mice were obtained. All animals used for experiments were 6–8-week-old female mice. Animals were maintained in compliance with the guidelines and protocols approved by the Animal Care and Use Committees at the University of Colorado Anschutz Medical Campus.

### 2.2. Cell Lines and Cell Culture

Cell lines EO771, EO771-TripZ-EV, and EO771-TripZ-200c were cultured in RPMI 1640 Medium with l-glutamine (ThermoFisher (Waltham, MA, USA), Cat. #11875093) supplemented with 5% fetal bovine serum (FBS) and 10 μ/mL Penicillin-Streptomycin (PS, ThermoFisher Cat. #15070063). Cells of BT549 (ATCC Cat. #HTB-122, RRID:CVCL_1092) and BT549 TripZ-200c were maintained in RPMI-1640 with 10% FBS, 1× MEM non-essential amino acids (ThermoFisher Cat. #11140050) and 10 μg/mL PS, and 5 μg/mL insulin. All cells were tested for mycoplasma contamination using the Barbara Davis Center BioResources Core at the University of Colorado. T cells were grown in RPMI 1640 Medium, 10 μ/mL PS, 10% FBS, 1× MEM non-essential amino acids, 1× GlutaMAX (ThermoFisher, Cat. #35050079), and 0.1 mM beta-mercaptoenthanol.

### 2.3. Flow Cytometry

Single-cell suspensions were obtained from tumor, spleen, peripheral blood, and cell culture. All tumors were minced by scissors and razor blade in 60 mm dishes with 5 mL of media (RPMI + PS, FBS-free) and then digested with 250 μL/5 mL media Liberase for 30 min at 37 °C. A quantity of 100 μL of 100 mM EDTA was added to stop 1 mL of enzymatic digestion (500 μL for 5 mL). Digested tissue was pipetted up and down 30 times using a glass Pasteur pipette and then passed through a 100-μm nylon filter to acquire single-cell suspensions. Spleens and peripheral blood were treated with 5 mL of ACK lysis buffer, followed rapidly by the addition of complete media (RPMI, 5% FBS, P/S). Cells were resuspended in flow cytometry staining buffer. All cells were stained with Live Dead Blue Cell Stain (ThermoFisher, Cat. #L23105) and stained at recommended dilution for 30 min with conjugated antibodies. Cell trace violet (CTV, ThermoFisher, Cat. #C34557) was used to label splenocytes in experiments involving CD8^+^ T cell proliferation.

### 2.4. Antibodies

Flow cytometry: CD45 PerCP (clone 30-F11), CD4 FITC (clone GK1.5), CD4 PacBlue (clone GK1.5), CD8b (clone YTS156.7.7), H-2K^b^/H-2D^b^ PE (clone 28-8-8) were purchased from Biolegend (San Diego, CA, USA). Zeb1 Alexa Fluor 488 (clone E2G6Y) was purchased from Cell Signaling. Western blot: Zeb1 (clone E2G6Y), GAPDH (clone D16H11) were purchased from Cell Signaling (Danvers, MA, USA).

### 2.5. Whole Cell Vaccine and Tumor Studies

To generate EO771 whole cell vaccines, cells were resuspended at 1 × 10^6^ cells/mL in RPMI supplemented with 5% FBS and irradiated at a dose of 5000 rads (50 Gy) with a (137)Cs irradiator. Cells were washed 2× with PBS and mice were injected intraperitoneally (IP) with 1 × 10^6^ irradiated EO771 cells plus adjuvant on day 21 and day 7. Adjuvant consisted of 40 μg/mouse of polyI:C (InVivoGen, Cat. #tlrl-pic) and 40 μg/mouse of CD40 mAb [25]. On day 0, mice were challenged with EO771 cells orthotopically in the 4th inguinal mammary pad. Mice were monitored for tumor growth every other day by calipers and when the tumor volume reached 1500 mm^3^, mice were sacrificed. Mice that developed ulcers were excluded from the study. In vaccination studies comparing EO771 ± miR-200c whole cell vaccines, cells were transfected with oligos prior to irradiation (see transient miR-200c model below). Cell lines of EO771 TripZ were not used in whole cell vaccine due to the antigenic nature of the TripZ vector.

### 2.6. CD8 Depletion

Three days prior to primary vaccination, mice were treated with 500 μg of In Vivo Plus anti-mouse CD8a antibody (53-6.7, BioXCell, Lebanon, NH, USA, Cat. #BP0004-1) injected IP (100 μL in PBS) followed by weekly injections of 250 μg. In Vivo Plus rat IgG2a isotype control, anti-trinitrophenol (clone 2A3, BioXCell Cat. #BP0089) was administered as a control. Two to three days post-treatment, single-cell suspensions from peripheral blood were isolated and stained as described above for CD8 depletion analysis.

### 2.7. Inducible miR-200c Model

An inducible lentiviral pTripZ-RFP vector (Dharmacon, Lafayette, CO, USA, Cat. #RHS4750) generated in the laboratory of Dr. Jennifer Richer, encoding the empty vector or precursor sequence for miR-200c (TripZ-EV or TripZ-200c, respectively) was transduced into EO771 cells using Polybrene (Sigma Aldrich, St. Louis, MO, USA, Cat. #TR-1003) per the manufacturer’s protocol. EO771-TripZ-EV cells and EO771-TripZ-200c cells represent expansion from multiple clones. The pTripZ vector has an RFP tag and EO771 TripZ-EV and EO771-TripZ-200c cells each went through selection via flow cytometry to generate non-leaky doxycycline (Doxy) inducible cell lines. To select for cells that induced high expression of miR-200c, only the EO771-TripZ-200c cells went through a second round of selection for cells with high RFP-positivity upon treatment with 1.0 μg/mL Doxy (Sigma Cat. #D9891). For all cell culture experiments, EO771-TripZ-EV or EO771-TripZ-200c cells were similarly treated ±1.0 μg/mL Doxy. BT549 TripZ-200c cells were generated as described by Rogers et al. [13].

### 2.8. Transient miR-200c Model

Mammary carcinoma cells were transfected in 10 mm dishes (1 × 10^6^–2 × 10^6^ cells/dish) through reverse transfection with 600 pmol of scramble control or miR-200c mimic (Thermo Fisher, Cat. #4464058 and Cat. #4464066, respectively) plus 30 μL of Lipofectamine3000. Sixteen hours post transfection, medium containing transfection reagents was removed and replaced with fresh medium appropriate to the cancer cell type.

### 2.9. Quantitative Real-Time PCR

Total mRNA was isolated using TRIzol (Thermo Fisher Cat. #15596018) extraction, according to the manufacturer’s protocol. A total of 500 μg of total mRNA was reverse transcribed into cDNA using iScript cDNA Synthesis Kits (BioRad, Hercules, CA, USA, Cat. #1708891), according to the manufacturer’s protocol. Expression (ΔΔCT) of genes of interest were determined relative to *Gapdh* by qRT-PCR using YBR Green PCR Master Mix (ThermoFisher Cat. #4309155), a 7500 Fast Real-Time PCR System (Applied Biosystems, Waltham, MA, USA), and 7500 Software (ver2.3, RRID:SCR_014596). Expression (ΔΔCT) of miR-200c was detected using TaqMan probes (ThermoFisher, Cat. #4427975, Assay ID 002300) and was presented relative to expression of *U6*. Primers are shown in Supplementary Table S1.

### 2.10. Cloning of EO771 to Express B7-1 (CD80)

Mouse B7-1 cDNA ORF Clone in Cloning Vector (SinoBiological, Beijing, China, Cat. #MG50446-G) was transfected into 5-alpha Competent *E. coli* cells (NEB, Ipswich, MA, USA, Cat. #C2987H), following manufacturer’s protocol. Colony PCR for B7.1 using the following primers: 5′ACGTACAGATCTATGGCTTGCAATTGTCAGTTGATG′3, 5′CATGCAGTCGACCTAAAGGAAGACGGTCTGTTCAGC′3 was performed to identify bacteria with the correct plasmid. All plasmids were verified by sequencing. B7.1 was ligated into the destination vector MSCV-IRES-Thy1.1 DEST (pMIT) (Addgene, Watertown, MA, USA, Cat. #17442). pMIT-B7.1 was transfected into Platinum E cells using Lipofectamine3000. Viral supernatant was collected and used to transduce EO771 cells. EO771-B7.1 expressing cells were selected for double positive Thy1.1/B7.1 expression by fluorescence-activated cell sorting (FACS) to avoid selecting for specific antigenic changes.

### 2.11. Western Blot

Cells were lysed using RIPA buffer supplemented with complete, Mini Protease Inhibitor Cocktail (Roche, Basel, Switzerland, Cat. #11836153001) for 30 min on ice. Cell debris was removed by centrifugation 12,000 rpm for 20 min at 4 °C. Protein concentration were determined using Pierce BCA Protein Assay Kit (ThermoFisher, Cat. #23225), according to manufacturer’s protocol. Proteins were reduced and denatured in 4× Laemmli Sample Buffer (Bio-Rad, Cat. #1610747) supplemented with 2-mercaptoethanol (Bio-Rad Cat. #1610710XTU) at 100 °C for 10 min. A total of 25 μg of protein was resolved on a 4–15% Mini-PROTEAN TGX gel (Bio-Rad Cat. #4561083) and transferred to a nitrocellulose membrane using the Trans-Blot Turbo Transfer System and Trans-Blot Turbo Transfer Packs (Bio-Rad, Cat. #170-4158). Protein expression was determined using Pierce ECL Western Blotting Substrate (ThermoFisher, Cat. #32109) and the Syngene G:BOX Chemi imaging system and GeneTools analysis software after overnight incubation with primary antibodies at 4 °C and 1 h incubation at room temperature with secondary antibody. All westerns presented in a single figure panel are from the same experiment and full uncropped images can be seen in Supplementary Figure S2.

### 2.12. RNAseq Library Preparation and Sequencing

All samples were prepared for RNA sequencing using the Universal Plus mRNA-seq library preparation kit with NuQuant (TECAN, Männedorf, Switzerland, Cat. #M01485 V8). Dual and uniquely indexed samples were pooled and sequenced on the same flow cell using the NovaSEQ 6000 (2 × 150 bp) with a target of ~100 million reads per sample. The RNAseq data are deposited in the National Center for Biotechnology Information’s Gene Expression Omnibus.

### 2.13. Raw Data Quality Control and Trimming

Fastq files were assessed for base and read quality using FastQC v0.11.9. Trimming and read removal, analyzed as pairs, were performed using Cutadapt v2.9 executed under Python v.3.6.10. Illumina universal adapters were removed, in addition to removing any pairs with a minimum read length less than 10 and Phred quality scores of less than 30. Trimmed sequences were analyzed through FastQC v0.11.9 a second time to ensure adapter removal and trimming were successfully applied.

### 2.14. RNAseq Alignment, Quality Control, and Transcript Quantification

RNAseq reads used for RNA variant calling were aligned using three different splice-aware aligners: STAR v2.7.3a, TopHat v2.1.1, and HISAT2 v2.2.1 [26,27,28]. Reads were aligned to the GRCm38.p6 primary assembly with the corresponding GTF annotation file from NCBI build accession ID: GCA_000001635.8. For STAR alignment, default parameters were used in addition to —twopassMode Basic and—outSAMattributes NH HI NM MD AS nM jM jI XS so the resulting bam files could be re-used in the alternative splice event detection analysis—sjdbOverhang 150 and—outReadsUnmapped Fastx were set to adjust for the 2 × 150 bp reads length and to filter all unmapped reads to a separate fastq file for quality control purposes. Default parameters were used for reads alignment using TopHat in addition to setting—library-type fr-secondstrand. Similarly, default parameters were used for HISAT2. Metrics for all alignments were collected using the log statistics generated by each software. Mapping quality was further assessed for all samples for each alignment software using PicardTools v2.21.1 CollectRnaSeqMetrics and samtools v1.8 flagstat [29]. Metrics from all sources were combined and plotted to confirm high quality mapping and to aid in the identification of any outliers. To further clean up alignments, the software Opossum was used to prepare alignments for variant calling, particularly to help to reduce SNP artifacts specific to RNA-seq variant calling [30]. Opossum was also used to remove improperly paired reads and to keep alignments with mapping quality >40. Transcript quantification was performed using Salmon v1.3.0 [31]. Default setting were used in addition to the—validate Mappings flag at runtime.

### 2.15. RNA Variant Calling and Quality Control

All samples generated from each aligner had variants called independently using Platypus v0.8.1 [32]. The following parameters were used for independent variant calling: —filterDuplicates = 0; minMapQual = 0; minFlank = 0; maxReadLength = 500; minGoodQualBases = 10; minBaseQual = 20. Following variants calling, each vcf was independently filtered to retain only SNPs passing default filtering parameters set by Platypus and retaining SNPs that a depth of had at greater than 10 reads using bcftools v1.11 [33].

A custom script was written to identify all SNPs concordant across all three aligners in each sample. SNPs that were not concordant across all aligners were discarded. Concordant SNPs were merged across all samples into a single large multi-sample VCF. All bam files were listed in a comma-separated list into a single Platypus command to backfill missing SNPs with the same parameters as previously described. For backfilling, the following additional parameters were specified at runtime: —minPosterior = 0—GetVariantsFromBAMs = 0—Source/path/to/multisample.vcf.gz. SNPs passing the Platypus default filtering thresholds were retained and the resulting VCF was normalized using vt v0.5772 for downstream neoantigen detection [34].

### 2.16. WES Variant Calling

Publicly available data paired-end fastq files from the EO771 cell line were downloaded from the European Nucleotide Archive with accession id ERS3019428 and ERS3019429 from study accession PRJEB30681 [35]. Data quality assessment and read clean-up was performed as previously described. Paired-end reads were aligned to the GRCm38 primary assembly using BWA v0.7.17 [36], followed by alignment QC and duplicate read removed using samtools v1.8 and PicardTools v2.21.1. Variants were called using Platypus v0.8.1 against the GRCm38 genome. Variants containing multiallelic sites were split into separate lines, and any variants that did not pass the standard default filters supplied by Platypus were removed, in addition to any variants that were not supported by at least 20 reads using BCFtools v1.11 and HTSlib v1.11. Passing variants were normalized using vt v0.5772.

### 2.17. Variant Integration and Annotation

Variants present in both the DNA WES data and RNA-seq variant data were retained along with their corresponding VCF information fields and merged to generate a multi-sample VCF. These variants were annotated using variant effect predictor (VEP) GRCm38 v102 [37]. Any variant that was annotated as one of the following feature types by VEP was considered for neoantigen analysis: non-synonymous/missense, frameshift, splice, start, stop, UTR, and regulatory.

### 2.18. Detection of Neoantigens

Every variant was assessed for MHC-I binding for H-2D^b^ and H-2K^b^ alleles, which pertain to the C57BL/6J mice genotype using peptide lengths of 8-mers and 9-mers. For each variant, the genomic sequence was extracted from starting from kmer_length −1 from the variant position to kmer_length +1 as the ending position to ensure every k-mer has the mutational variant of interest represented at every nucleotide in the peptide. Peptide binding affinity for both the mutant and wildtype peptide counterparts was assessed using NetMHC v4.0 [38,39]. Data from the variant integration and annotation step was merged into each set of NetMHC results independently, in addition to the transcript quantification results. Any variant that did not contain at least one transcript that had a quantification threshold of at least one transcript per million (TPM) was filtered out. Additionally, any variant that did not have any predicted binding affinity to the tested MHC-I allele was also filtered out.

### 2.19. Detection of Neojunctions

The sorted bam outputs from STAR were used as input into SplAdder v2.4.4 [22] to generated splice graphs for alternative splicing event detection with interaction convergence set to 10 and confidence levels set to 2. Differential alternative splicing between biological groups was also generated using SplAdder. Detected splice events by SplAdder output were read into Python and each event was assessed for whether the event contained a novel junction. Novel junctions were identified based upon whether the junctions detected in the STAR output of inputSJ.out.tab files were present in the annotated GTF. Any junction that had been identified as previously annotated was filtered out. Junction regions were extracted and all open reading frames were assessed. The proper reading frame was identified using a custom protein blast database that we generated and the junction was translated so that it encompassed every possible 9-mer position upstream and downstream of the junction that resulted in at least a single amino acid change overlap. Peptides were removed that were less than the 9-mer length post-translation or if it resulted in a premature stop that was upstream of the novel junction site. The peptides were then used as input into NetMHC v4.0 to determine binding capability by the MHC-I alleles previously described. Any peptides that had no predicted binding capability were removed. More information on this portion of the analysis is in our GitHub repository, https://github.com/tbrunetti/neoantigENcyclopedia, accessed on 1 June 2022.

### 2.20. Intron Retention (IR) Validation

To identify intron retention events expressed differently in EO771 cells ± miR-200c, IR events were filtered as follows: (1) mean intron confidence >5 alignments spanning the intron in at least one group, (2) the difference in percent spliced in (PSI) between EO771-EV versus EO771-miR-200c, and (3) visual validation of IR using IGV_2.8.11. Primers were designed against the flanking exons of the intron retention events meeting the above criteria to confirm expression using cDNA from EO771-EV and EO771-miR-200c via RT-PCR. cDNA was also made from peripheral tissue (spleen) and IR events found in the spleen were excluded from further studies. Primers are in Supplementary Table S2.

### 2.21. T Cell Assays

Spleens were harvested from mice, crushed in a filter of 100 microns and lysed in ACK lysis buffer (ThermoFisher Cat. #A1049201) for 5 min at room temperature to remove red blood cells. Splenocytes were plated in 24-well dish at 4 × 10^6^ cells/well with either 10 μg peptide or 1 × 10^5^ EO771-B7.1 cells in the presence of 20 units IL-2. Cells were then collected after 7 days and challenged with either peptide or EO771-B7.1 cells for either 3 days for flow cytometry proliferation assays or 24 h for LDH cytotoxic assays (Biolegend Cat. #426401) and IFNγ ELISA (ThermoFisher, Cat. #88-7314-22) following manufacture’s protocol.

## 3. Results

### 3.1. Antigens from Mesenchymal-like EO771 Whole Cell Vaccine Elicit CD8^+^ T Cell-Specific Antitumor Immunity

**Figure 1:**
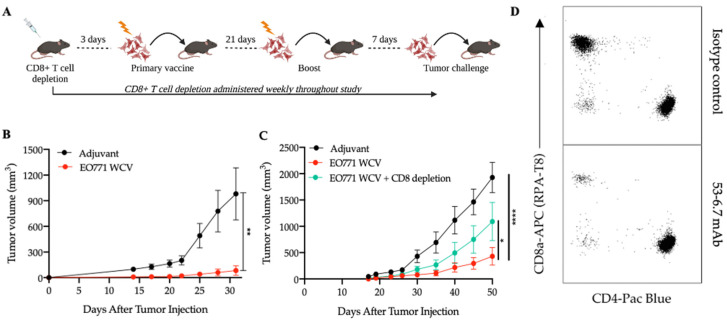
CD8^+^ T cells are required for protection against mesenchymal-like EO771 tumor growth using EO771 whole cell vaccine (WCV). (**A**) Overview of tumor studies is shown. (**B**) Tumor growth curve of EO771 cells in mice treated with adjuvant alone (*n* = 10) or EO771 WCV (*n* = 10). Error bars depict mean with SEM. A two-way ANOVA was performed, and significant protection was provided by the EO771 WCV (*p* = 0.0052). This experiment represents 4 tumor studies. (**C**) Tumor growth curve of EO771 cells in mice treated with adjuvant (*n* = 16), EO771 WCV (*n* = 16) or EO771 WCV plus 53-6.7 mAb (*n* = 16). Graph represents 2 independent experiments; error bars depict mean with SEM. A two-way ANOVA was performed using Dunnett’s multiple comparisons test. At day 50, there was significant protection against tumor growth in mice treated with EO771 WCV (*p* < 0.0001), which was partly reversed in mice that were also CD8-deficient (*p* = 0.0157). (**D**) Representative plots from flow cytometric analysis depicting CD8^+^ T cell depletion from the peripheral blood of mice treated with CD8^+^ T cell depleting mAb (clone 53-6.7) or isotype control mAb (clone 2A3).

The murine EO771 cancer cell line is a mesenchymal, basal-like, spontaneously metastasizing mammary cancer model that effectively mimics human breast cancer [40,41]. To evaluate the potential of this model for antigen discovery, we first investigated its immunogenicity as a whole cell vaccine (WCV). To this end, mice were vaccinated with irradiated EO771 cells plus adjuvant consisting of poly I:C, a TLR3 agonist shown to cause robust antigen-specific killing against apoptotic cells in C57BL/6 (WT) mice [42], and anti-CD40 antibody, a potent dendritic cell activator [25]. Control mice were treated with adjuvant alone (Figure 1A). The vaccine containing irradiated EO771 cells provided significant protection from tumor growth for the duration of all experiments relative to adjuvant-treated mice (Figure 1B). To assess the role of CD8^+^ T cells in this antitumor response, we depleted CD8^+^ T cells in mice receiving the irradiated EO771 cell vaccine using the 53-6.7 mAb (Figure 1D) [43]. As expected, mice that received the CD8^+^ depleting antibody, in addition to the EO771 whole cell vaccine, lost a significant amount of protection against tumor growth compared to the CD8^+^ T cell-sufficient mice (Figure 1C). In similar studies involving CD4^+^ T cell depletion using the GK1.5 mAb, there was no change in protection provided against tumor growth by the EO771 WCV suggesting a limited role of CD4^+^ T cells in this particular anticancer vaccine (Supplementary Figure S1). Thus, these results provide a rationale to identify neoantigens and develop MHC class I-based antitumor vaccines using this model as proof of principle.

### 3.2. Restoration of miR-200c in Mesenchymal EO771 Cells Effectively Reverses the EMT Program

**Figure 2:**
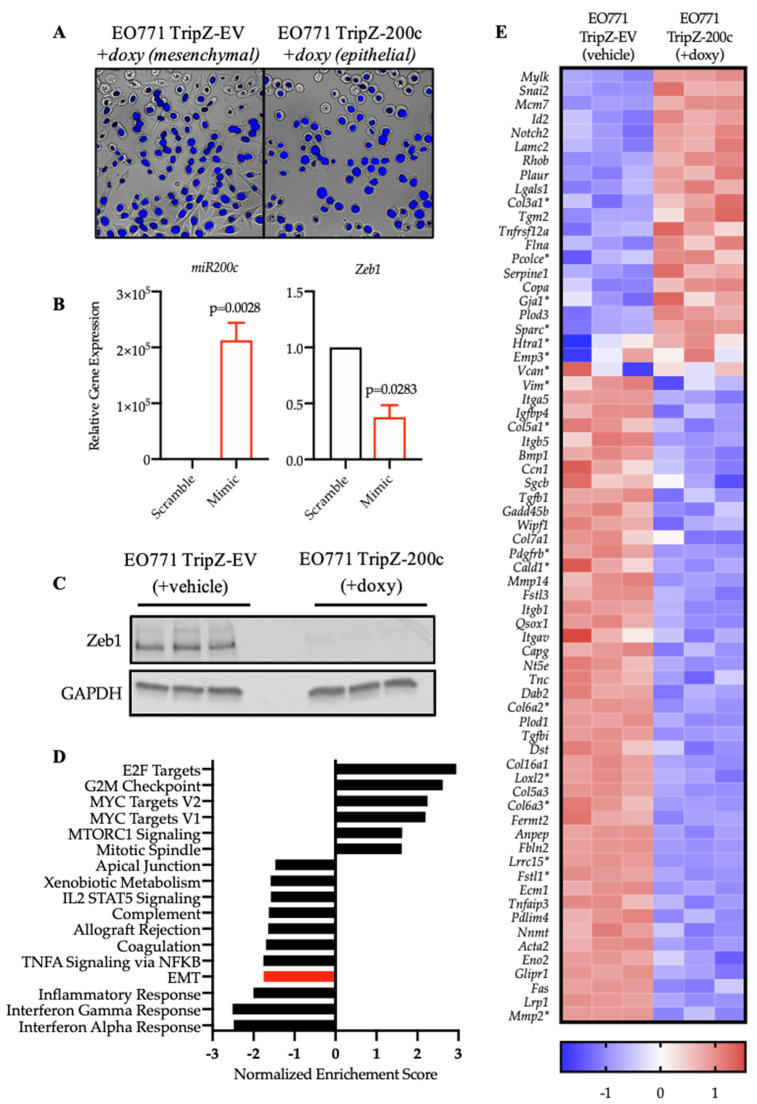
Restoration of miR-200c in mesenchymal-like EO771 murine mammary cancer cells restores a more epithelial phenotype. (**A**) Representative images of the change in morphology of EO771 cells in the presence of miR-200c. Cells were stained with DAPI and visualized by microscopy (*n* = 2, with 10 fields of view). (**B**) Relative gene expression of miR-200c and Zeb1 by qRT-PCR analysis following treatment with miR-200c mimic or scrambled oligo is shown. Data were normalized to either *U6* or *Gapdh* and are presented as fold change relative to the scrambled oligo. (**C**) Western blot analysis revealed downregulation in Zeb1 protein levels with miR-200c restoration. GAPDH was used as a loading control. (**D**) Gene set enrichment analysis (GSEA) identified the top 15 altered “Hallmark” pathways along with their respective normalized enrichment score (NES). Epithelial to mesenchymal transition (EMT) is highlighted in red. (**E**) Heatmap of the top altered genes from the Hallmark-EMT pathway that were altered by miR-200c restoration. Each column represents an individual biological replicate of EO771-TripZ-EV (Vehicle-treated, *n* = 3) or EO771-TripZ-miR-200c (+doxy, *n* = 3) for 72 h. Asterisks (*) indicate similarities found in Mak et al. [47], a published human pan-carcinoma EMT signature. Genes depicted as Z-scores.

To understand the degree to which miR-200c restoration alters antigen expression, an inducible model of miR-200c overexpression was utilized. We introduced a TripZ-empty vector (TripZ-EV) control or TripZ-miR-200c (TripZ-200c) vector into EO771 cells [12]. Morphological changes in cells undergoing EMT or MET represent a hallmark of this transition [44]. Restoration of miR-200c caused a striking change in morphology of EO771-TripZ-200c cells, from being elongated and fibroblast-like to compact and round (Figure 2A). As expected, miR-200c expression increased significantly with doxycycline treatment in EO771-TripZ-200c cells, and the expression of *Zeb1*, a direct target of miR-200c, decreased significantly [45,46] (Figure 2B). The effect of miR-200c restoration within these cells could also be detected through western blot analysis as a loss of Zeb1 protein in miR-200c-treated cells (Figure 2C).

To characterize the effect of miR-200c restoration more comprehensively on EO771 cells, we performed bulk RNAseq analysis on EO771-TripZ-EV cells treated with vehicle or doxycycline (mesenchymal-like) and EO771-TripZ-miR-200c cells treated with doxycycline (epithelial-like). Gene set enrichment analysis (GSEA) showed that the Hallmark-EMT pathway was within the most significantly decreased pathways following miR-200c restoration [48] (Figure 2D). Interestingly, a number of immune-related pathways are among the most altered Hallmark Pathways, consistent with a body of evidence that has shown the relationship between carcinomas cells undergoing EMT and modulation of the immune system (discussed above). Analysis of the top altered genes within the Hallmark-EMT pathway revealed upregulation and downregulation of well-established EMT-related genes (Figure 2E).

Notably, when compared to a published human pan-carcinoma EMT signature from Mak et al. [47], there was significant overlap in the EO771 genes upregulated or downregulated by miR-200c (marked with asterisks in Figure 2E), demonstrating miR-200c restoration in EO771 cells recapitulates EMT profiles found across human cancers. Finally, we interrogated overall differential gene expression in EO771 cells ± miR-200c to assess the scale of potential mesenchymal-associated and epithelial-associated antigens and found over 1500 genes significantly upregulated or downregulated (Supplementary Table S3) upon the induction of miR-200c (*p*.adj. < 0.05, log_2_FC > ±1.2), highlighting the profound impact on gene expression that occurs as cells undergo EMT and the vast potential for antigen discovery [24].

### 3.3. Reversal of EMT Elicited a Superior Cytotoxic Response against EO771-miR-200c Target Cells

**Figure 3:**
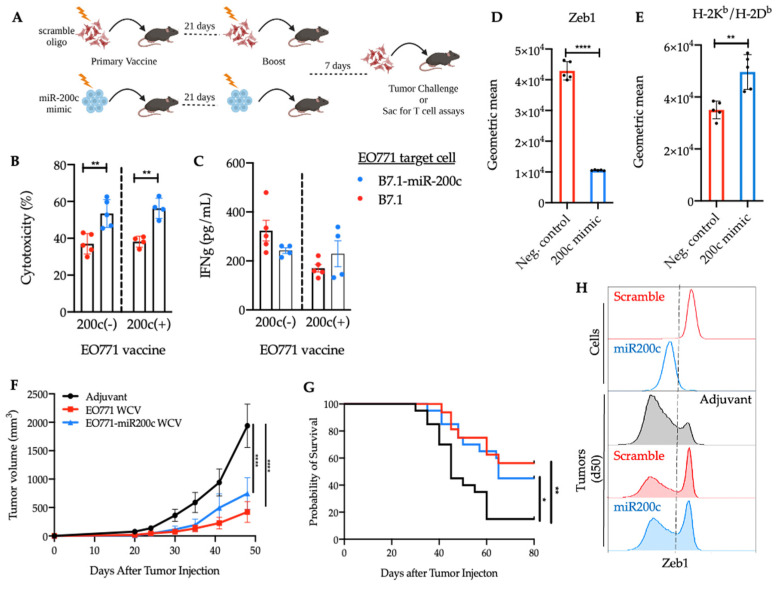
Following miR-200c restoration, enhanced cytotoxicity against EO771 target cells is associated with MHC class I expression. (**A**) Overview of study design. (**B**) Quantification of percent cytotoxicity and (**C**) IFNγ measurements from splenocytes of mice against EO771-B7.1 ± miR-200c target cells following vaccination with EO771 WCV ± miR-200c. Cells were co-cultured at E:T ratio of 20:1. Target cells are shown in the figure legend. A two-way ANOVA was performed using Sidak’s multiple comparisons test, finding a significant increase in cytotoxicity against EO771-B7.1-miR-200c+ target cells in both the EO771 WCV (*p* = 0.001) and EO771-miR-200c WCV (*p* = 0.0012) groups. Data are representative of three independent experiments and are shown as mean ± SD. (**D**) Quantification of geometric mean of Zeb1 (*n* = 5) and (**E**) MHC class I (*n* = 5) on EO771 cells transfected with miR-200c for 72 h by flow cytometric analysis is shown. An unpaired *t* test shows a significant difference in Zeb1 expression (*p* < 0.0001) and MHC class I expression (*p* < 0.024) following miR-200c transfection. Data are representative of three independent experiments and are shown as mean ±SD. (**F**) Tumor growth curve of EO771 (mesenchymal-like) cells in mice treated with adjuvant only (*n* = 20), EO771 WCV (mesenchymal-like) (*n* = 16) or EO771-miR-200c treated WCV (epithelial-like) (*n* = 20). Error bars depict mean with SEM. A two-way ANOVA was performed using Tukey’s multiple comparisons test and significant protection was provided by the EO771 WCV (*p* < 0.0001) and EO771-miR-200c WCV (*p* < 0.0001). (**G**) Survival curve of the same mice as in f; log rank (Mantel-Cox) test = 0.0035. (**H**) Representative histograms of change in Zeb1 expression in EO771 tumors at day 50 in mice that received adjuvant, EO771 WCV, or EO771-miR-200c vaccine relative to baseline *Zeb1* expression in EO771 cells ± miR-200c. The numerical *p* values listed in the figure legend correlate with the asterisks in the figure.

The results from Figure 2 demonstrate that restoration of miR-200c in EO771 cells effectively reverses the EMT program, in addition to having a profound impact on overall gene transcription. To test whether this impact on gene regulation also effects tumor immunogenicity (ability to elicit T cells) or antigenicity (ability to be recognized by T cells), we used oligonucleotide mimics that encode the seed sequence of miR-200c and bind target 3′UTRs but do not present the immunogenic caveats of the inducible vectors encoding miR-200c. To this end, we evaluated EO771-scramble-treated (mesenchymal-like) and EO771-miR-200c-mimic-treated (epithelial-like) whole cell vaccines for stimulation of antitumor immunity as measured by (1) cytotoxic response against EO771-B7.1 ± miR-200c target cells and (2) protection from EO771 tumor growth (Figure 3A). We first introduced the costimulatory B7.1 (CD80) molecule into EO771 cells, which is normally expressed by antigen presenting cells, providing signals for proliferation and differentiation, in addition to antigen-specific signaling (TCR:pMHC). Thus, EO771-B7.1 cells provide antigen-specific and co-stimulatory signals to CD8^+^ T cells in the absence of other immune cells and effectively act as both the target cell and antigen presenting cell [49,50].

Splenocytes from mice that received either the EO771 WCV (mesenchymal-like) or the EO771-miR-200c WCV (epithelial-like) demonstrated superior cytotoxicity against EO771-miR-200c-B7.1 target cells compared to EO771-B7.1 target cells (Figure 3B) and while IFNγ was detected in the supernatant of all groups (Figure 3C), there was not a direct correlation with cytotoxicity. Next, we interrogated MHC class I levels on EO771 cells ± miR-200c and found Zeb1 significantly downregulated (Figure 3D) and MHC class I (Figure 3E) to be significantly upregulated in the miR-200c-treated cells. This suggests that more-mesenchymal cells possess cell-intrinsic immunoevasive properties that are not fully overcome by orchestrating a mesenchymal-directed adaptive immune response. Furthermore, in addition to the role of CD8^+^ T cells, other innate effector immune cells should be considered in the context of altered cytotoxic responses and MHC class I expression, such as NK cells, which remain to be explored.

In line with these findings, we found no significant difference in EO771 tumor growth or survival in mice vaccinated with EO771 WCV (mesenchymal-like) or the EO771-miR-200c WCV (epithelial-like) WCV and subsequently challenged with EO771 WT cells (mesenchymal-like) (Figure 3F,G). However, upon further investigation of the tumor and its EMT status, the adjuvant-treated mice had tumors consisting predominantly of a Zeb1 low cells (more epithelial) rather than the Zeb1 high cells (more mesenchymal) that were injected, suggesting these tumors are dynamic [51] and undergo a mesenchymal to epithelial transition (MET) in vivo. Interestingly, mice that received the EO771 WCV ± miR-200c had a significant reduction in the Zeb1 low population (Figure 3H). This suggests both WCVs elicit an immune response directed at the Zeb1 low population (more epithelial), which is consistent with the enhanced cytotoxic phenotype seen against EO771-miR-200c-B7.1 target cells seen ex vivo.

### 3.4. SNV-Derived Antigens Upregulated in Mesenchymal EO771 Cells Elicit Superior Cytotoxic Responses against EO771-miR-200c+ Target Cells

**Figure 4:**
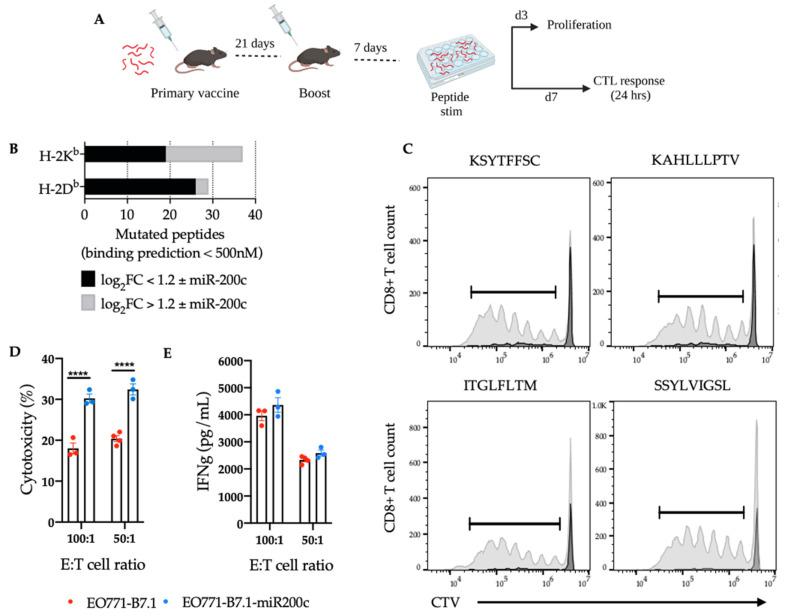
Restoration of miR-200c in EO771 alters expression of MHC class I SNV-derived neoantigens and putative SNV-derived peptides upregulated in mesenchymal-like EO771 cells elicit superior cytotoxic responses against epithelial-like EO771-miR-200c+ cells. (**A**) Overview of study design vaccinating mice with pooled MHC class I peptides followed by ex vivo T cell assays. (**B**) Quantification of putative peptides resulting from somatic variants per H-2D^b^/H-2K^b^ allele with strong binding affinity predictions (<500 nM) is shown. The gray bar fraction corresponds to the number of peptides with significant change in expression ± miR-200c. (**C**) Flow cytometry analysis depicting CD8^+^ T cell proliferation in response to stimulation with individual MHC class I SNV-derived peptides. The gray peaks represent the dilution of cell trace violet (CTV) stain as CD8^+^ T cells proliferate in response to specific peptide. The black peak represents CTV stain in non-proliferating CD8^+^ T cells treated with negative control (no peptide). (**D**) Quantification of percent cytotoxicity and (**E**) IFNγ measurements from splenocytes of mice vaccinated with pooled peptides expressed more in the mesenchymal conditions and challenged with EO771-B7.1 ± miR-200c target cells ex vivo at the respective Effector: Target cell ratio (E:T). A two-way ANOVA was performed using Sidak’s multiple comparisons and found significant differences in cytotoxicity against EO771-B7.1-miR-200c target cells at both E:T ratios (*p* < 0.0001). The numerical *p* values listed in the figure legend correlate with the asterisks in the figure.

SNV-derived neoantigens are novel epitopes from accumulated mutations within the cancer genome capable of expanding CD8^+^ T cell populations when presented on MHC class I. To understand the mutational burden in EO771 cells and the difference in mutational expression as cells undergo EMT, we performed variant calling from our RNAseq data. We identified 9 genes with 10 mutant MHC class I-specific peptides containing an SNV upregulated in the mesenchymal state and 6 genes with 11 MHC class I-specific mutant peptides upregulated in the epithelial state (Figure 4B).

Five of the strongest mesenchymal-related MHC class I mutant peptides (Table 1) were selected for (1) CD8^+^ T cell proliferation and cytotoxic responses against EO771-B7.1 ± miR-200c target cells in culture and (2) protection against EO771 tumor growth when delivered as a pooled vaccine in vivo. To this end, mice were vaccinated and boosted with pooled peptides upregulated in the more mesenchymal state and 7 days later, splenocytes were stimulated with individual peptides to assess CD8^+^ T cell proliferation as well as cytotoxicity against EO771-B7.1 ± miR-200c target cells in addition to IFNγ production (Figure 4A). Quantification of putative peptides showed a significant difference in peptides that change in expression with and without miR-200c (Figure 4B). Four out of the five peptides elicited strong CD8^+^ T cell proliferation, indicating these particular peptide:MHC complexes activated a responding CD8^+^ T cell-specific repertoire (Figure 4C and Supplementary Figure S3). Interestingly, T cells that were primed using these peptides and then challenged with EO771-B7.1 ± miR-200c target cells elicited stronger cytotoxic responses and IFNγ against EO771-miR-200c-B7.1 cells (Figure 4D,E), despite being identified as upregulated in the mesenchymal RNAseq variant calling. Lastly, these peptides were tested for protection against EO771 tumor growth in mice and we found no significant difference in protection versus irrelevant peptide (data not shown). Together, these experiments suggest that not all neoantigens are sufficient to drive an antitumor response in vivo and the protection seen from our whole cell vaccine is driven by multiple effector immune cells as well as untested antigens.

### 3.5. Reversal of EMT Alters RNA Splicing Factors and Splicing Events in EO771 Cells That Are Conserved in Human BT549 Breast Cancer Cells

Recent analyses of the human transcriptome have shown that alternative splicing is increased in cancer samples compared to matched normal samples by up to 30% [21,52]. Although the bioinformatics suggest that these neojunctions may in fact be good targets for immunotherapies, it has yet to be determined whether antigens derived from these neojunctions (defined as exon-exon junctions derived from alternative splicing found predominately in tumor samples) elicit antitumor T cell responses.

**Figure 5:**
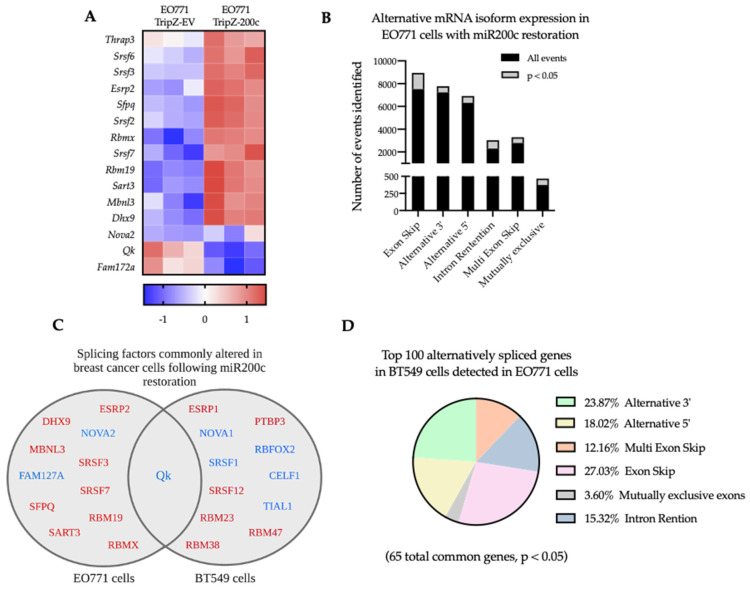
Alterations in RNA splicing in murine EO771 cells are conserved in human BT549 breast cancer cells following restoration of miR-200c. (**A**) Heatmap of splicing factors expressed differentially in EO771 cells ± miR-200c identified through “GO_Alternative_mRNA_Splicing_via_Spliceosome” GSEA is shown (*p* < 0.05, log_2_FC > 1.2). (**B**) Quantification of genes alternatively spliced in EO771 cells ± miR-200c using SplAdder software. Graph depicts the total number of events of each type (black) and the number of events that are significantly different between EO771-EV and EO771-miR-200c (*p* < 0.05). (**C**) Venn diagram shows splicing factors undergoing change in transcription following miR-200c restoration in EO771 cells and human BT549 cells (*p* < 0.05). Red transcripts increase and blue transcripts decrease. (**D**) Pie chart depicting alternatively spliced genes shared in BT549 cells and EO771 cells relative to the splicing event that gene is undergoing in EO771 cells (*n* = 65).

In EO771 cells, gene set enrichment analysis (GSEA) identified the GO-Alternative mRNA Splicing pathway as significantly altered (*p*.adj = 0.042, NES 1.8), following miR-200c restoration. Within this pathway, 15 splicing factors were significantly up or downregulated with miR-200c restoration (*p* < 0.05, log2FC > 1.2), specifically splicing factors typically expressed in epithelial cells were increased upon induction of miR-200c, while the more mesenchymal-like splicing factors, such as QKI and Fam172a decreased [24] (Figure 5A). Furthermore, adapting a recently developed methodology developed by Kahles et al. [21,22] that constructs splicing graphs from RNAseq data, we identified 3885 splicing events that were significantly different between EO771 cells ± miR-200c across 6 types of splicing events (Figure 5B).

Since EMT-related alternative splicing is program-specific, we expect some of the same EMT related events to be shared across other types of carcinomas and some commonalities between mammals. We compared our top altered splicing factors and alternatively spliced genes to the human BT549 cell line ± miR-200c restoration. Among the top 100 genes found to be alternatively spliced in the human BT549 breast cancer cells, 65 of the same genes were also identified as alternative spliced in the mouse mammary EO771 cell line upon miR-200c restoration (Figure 4D, Supplementary Tables S4 and S5), despite finding only one conserved splicing factor (QK) (Figure 5C). Of note, a number of the significantly altered splicing factors upon miR-200c restoration in EO771 cells and BT549 cells represent highly homologous RNA-binding proteins with redundant functions, including NOVA1/NOVA2 [53] and ESRP1/ESRP2 [54]. Comparison of the spliced genes shared in human BT549 cells and mouse EO771 cells relative to the splicing event that gene is undergoing in EO771 cells suggests that similar genes are spliced (Figure 5D).

### 3.6. Validation of Neojunction-Derived Antigens from Alternative Splicing in EO771 Cancer Cells

In silico approaches in combination with mass spectrometry have identified neoepitopes from intron retention events that are processed and complexed with MHC class I molecules on the surface of cancer cells [23]. However, in vitro or in vivo approaches have yet to demonstrate CD8^+^ T cell immune responses against these neoepitopes. Using the SplAdder algorithm, we identified 1288 putative tumor-specific intron retention (IR) events in the EO771 cells. Next, from each IR event, splice junctions of the tumor isoform were translated into peptides, generating 2715 putative tumor-associated epitopes. Percent Spliced In (PSI), a measure of splicing event abundance, was used to prioritize peptides derived from IR events differentially expressed in EO771 tumor cells ± miR-200c with the intent of targeting EO771 cells in either a more mesenchymal or epithelial cell state.

**Figure 6:**
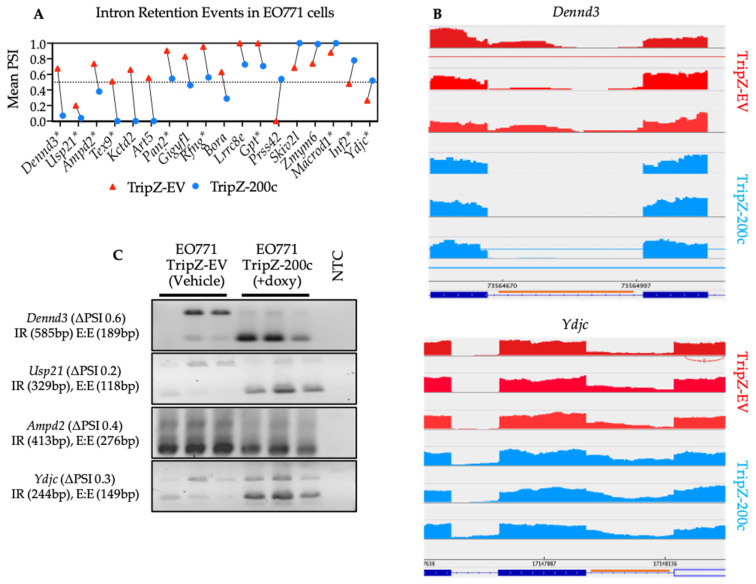
Validation of differentially expressed intron retention events in EO771 cells ± miR-200c. (**A**) Strip plot depicting genes of interest with predicted significant change in mean Percent Spliced In (PSI) of IR events following miR-200c restoration. Genes marked with asterisks (*) showed that splicing events with the Integrative Genomics Viewer (IGV). (**B**) Representative sashimi plots generated using IGV software. The splicing graph depicts the retained intron (orange line) between its flanking exons. (**C**) Representative images of RT-PCR validation of cDNA depicting intron retention events in EO771 cells following miR-200c restoration. When two bands are present, the larger bands correspond to the variant with the retention (IR), whereas the smaller band corresponds to the variant with the normal exon-exon junction (E:E).

Of the 18 IR events with differences in PSI, only 10 (marked with asterisks in Figure 6A) were visualized using the Integrative Genomics Viewer (IGV) [55] (Figure 6A,B). Ten additional IR events were selected based on mean intron confidence criteria only. To validate these splicing events, primers were designed against the flanking exons for RT-PCR using cDNA from EO771-EV, EO771-miR-200c cells and spleen (to assess IR expression in non-cancer tissue). Of the 20 IR events that underwent validation by PCR, 4 were not detected and of the remaining 16 events that were detected, only 4 were unique to the EO771 cancer cells and also not found in the spleen cDNA. The validated, tumor-specific IR events occur in *Dennd3*, *Usp21*, *Ampd2* and *Ydjc* (Figure 6C) and their respective putative peptides and predicted H-2K^b^ and/or H-2D^b^ binding affinities are listed in Table 2.

These IR-derived peptides were tested for stimulation of CD8^+^ T cell proliferation. To this end, five mice were vaccinated with pooled peptides and 7 days later, splenocytes were isolated and stimulated for 4 days with individual peptides to assess CD8^+^ T cell proliferation by flow cytometry. No proliferation was detected (data not shown), suggesting these peptides may be self-derived and reactive T cells have been deleted or tolerized. These results also highlight the importance of considering multiple tissue types and developmental time points as normal control to identify “cancer-specific neojunctions”.

## 4. Discussion

In most cancers, treatments become less effective as the disease progresses and resistant cells can recur as metastatic disseminated disease. The more mesenchymal TNBC subtype recurs more rapidly (within the first few years post-diagnosis), while the more epithelial estrogen receptor positive or luminal subtype often takes much longer to metastasize [56]. EMT is thought to facilitate the intravasation step of the metastatic cascade, while MET (reversion back to an epithelial-like state) is thought to facilitate outgrowth at the metastatic site. This plasticity of tumor cells thus facilitates the process of metastasis [57]. To reveal changes in breast cancer immunogenicity associated with EMT/MET plasticity in a simplified system, we used miR-200c to cause a transition of the more mesenchymal-like cells to undergo MET and become more epithelial-like, with the ultimate goal of contributing to antigen-specific immunotherapies that target the more plastic and metastatic breast cancer cells. As expected from other recent studies [17,58,59] and consistent with the tenet that more epithelial-like ER+ breast cancers progress more slowly, we found that the epithelial-like miR-200c-competent cells express more antigen-presenting molecules, which provides more potential T cell targets than the mesenchymal counterparts.

In addition to expression of MHC molecules, T cells also require recognition of peptide antigens to which they are not tolerized for activation. Although few mutated antigens are shared among breast cancers, previous studies have established increased and aberrant splicing patterns in breast and other cancers (reviewed in [60]). Rätsch’s group described an in silico method for identification of many potential cancer-specific antigens derived from alternative splicing, also known as neojunctions, inspiring further investigation into these antigens [21]. Using their computational analysis as a guide, we identified 2715 potential neoantigens generated from intron retentions predicted to be presented by MHC molecules. Of these, we tested 10 that were predicted to be immunogenic. We have not yet tested those predicted from other types of splicing events, such as the exon skip, multi-exon skip, and 3′ and 5′ alternative splicing events where neojunctions may lead to immunogenic peptides.

While identifying these neojunctions, we encountered several computational limitations that we attempted to overcome. To maximize reproducibility when identifying the SNVs, we used three different alignment mutation calling algorithms and only used overlapping results for the final investigation. Identification of neojunction-derived peptides requires multiple critical analytic steps including alignment, novel splice event detection, and peptide: MHC binding algorithms. Each computational analysis is prone to a degree of error that only compounds per analysis, generating high false discovery rates and low validation rates. Our data suggest that low validation rates are high in neojunction-derived peptides (0/10 elicited T cell proliferation) relative to the less computationally intensive steps involved in identifying the SNV-derived peptides (4/5 elicited T cell proliferation). We expect that future computational tools, more technical replicates, and more precise sequencing technology will improve these limitations and result in a better “hit rate” in neojunction-derived neo-antigen prediction.

Despite these computational challenges, using a model of miR-200c restoration we have shown that alterations in the expression of SNV-derived and neojunction-derived antigens occur as a result of MET. We carried out an in-depth analysis of intron retention events using a novel approach to validate these splicing events and took the next step to test the immunogenicity of the predicted resultant neoantigens using T cell assays. Across all antigens tested, enhanced CD8^+^ T cell cytotoxicity was measured against miR-200c-expressing EO771 cells, suggesting that miR-200c and the cells that express it provide an antigen-non-specific boost in immunogenicity relative to cells that have lost miR-200c.

## 5. Conclusions

In the present study, we utilized miR-200c to identify potential splicing events that play a role in breast cancer cell plasticity that may affect tumor progression by affecting tumor immunoevasion. The data show that restoration of miR-200c to the mammary cancer cell line EO771 alters gene expression, splicing factor expression, and splicing events, as it does in human TNBC lines (as demonstrated here and prior studies [24]). For the first time, our study investigates potential effects on antigen expression and the ensuing CD8^+^ T cell response. While the Rätsch’s group identified copious cancer-specific splicing events from The Cancer Genome Atlas data [21], they did not test immunogenicity. As previously observed upon modulation of EMT, the level of MHC class I increased in the presence of miR-200c, but the SNV- and neojunction-derived neoantigens also changed, providing an avenue for targeting the plasticity-associated neoantigens. RNA splicing was recently found to control colorectal cancer cell plasticity [61]. In this study, we considered neoantigens generated by miR-200c-mediated alterations in gene expression and gene splicing due to altered expression of splicing factors (SNV and neojunction-derived neoantigens, respectively) in EO771 cells following miR-200c restoration as a means to target mesenchymal cells and found mesenchymal-associated antigen elicited superior cytotoxicity against epithelial counterparts. The study highlights the extreme complexity involved in accurate prediction of neojunction-derived antigens resulting in low validation rates [52]. Thus, translating neojunction-derived antigens to anticancer immunotherapy will require additional layers of computational biology combined with new and existing technologies, including some that have long been a benefit to antigen discovery, such as mass spectrometry. The intersection of these complementary methods will facilitate the accurate identification of neojunction-derived neoantigens and enable design of vaccines to prevent breast cancer recurrence/progression.

## Figures and Tables

**Table 1:** SNV-derived peptides selected for CD8^+^ T cell immunogenicity assays.

Gene	Mutant Peptide	Allele	Log_2_FC (EO771-EV/EO771-miR-200c)	Binding Affinity (nM)
*Extl1*	VWQSFPEL	H-2K^b^	1.56	20.3
*Mt-Cytb*	ITGLFLTM	H-2K^b^	2.7	120.2
*Mt-Nd5*	SSYLVIGSL	H-2K^b^	2.53	25.3
*CD46*	KSYTFFSC	H-2K^b^	2.03	36.2
*Ptprt*	KAHLLLPTV	H-2D^b^	1.22	451

**Table 2:** Intron retention-derived peptides and predicted MHC class I binding affinities selected for CD8^+^ T cell immunogenicity assays.

Gene	Neojunction-Derived Peptide	H-2D^b^ Binding (nM)	H-2K^b^ Binding (nM)
*Dennd3*	VTLRSQRGL	-	420.6
	TTHLHSPPL	-	172.4
	SKVVSATPL	707.4	-
	RGLKNMLSA	1704.4	-
*Usp21*	ILIFTFLFL TFLFLLGYL	6191	902
		6003.4	779
	SSPSFEFPL	382	13
	LIFTFLFLL	2071	53.6
	FTFLFLLGY	-	1175.5
	CHPDFLCHL	-	371.6
*Ampd2*	VSGGYWVPL	-	50.1
*Ydjc*	VHVLPGTRL	-	966.3

## Data Availability

RNAseq data of the EO771 cells +/− miR-200c is deposited at GEO, accession ID GSE212537.

## Supplementary Materials

The following supporting information can be downloaded at: https://www.mdpi.com/article/10.3390/cancers14184397/s1, Figures S1–S3: Limited role of CD4+ T cells in immunity elicited by EO771 WCV against mesenchymal EO771 tumor growth, Uncropped western blot images and densitometry from Figure 2C, and Gating strategy to identify proliferating CD8+ T cell population from Figure 4B flow cytometry analysis; Tables S1 and S2: Primers for qRT-PCR and Primers for intron retention events; Table S3, Differential gene expression in EO771 cells following miR200c restoration; Tables S4 and S5, Alternatively spliced genes in TNBC cells following miR-200c restoration and Alternatively spliced genes in TNBC cells following miR-200c restoration.
