# Supplementary material for: Antigens Expressed by Breast Cancer Cells Undergoing EMT Stimulate Cytotoxic CD8+ T Cell Immunity
Source: Cancers (Basel). 2022 Sep 9;14(18):4397. doi: 10.3390/cancers14184397 (PMC9496737; doi:10.3390/cancers14184397)

Figure S1. Limited role of CD4+ T cells in immunity elicited by EO771 WCV against mesenchymal EO771 tumor growth.

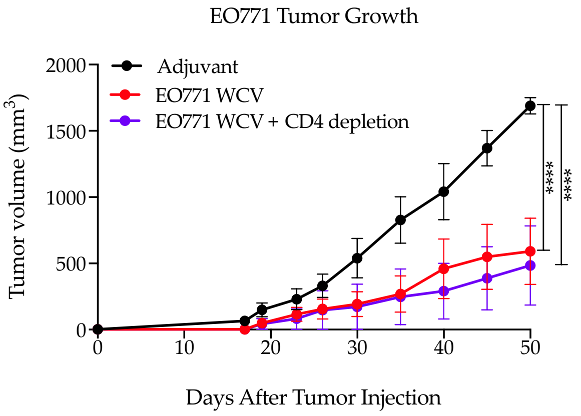

Tumor growth curve of EO771 cells in mice treated with adjuvant (n=5), EO771 WCV (n=5) or EO771 WCV plus CD4+ T cell depletion (Ab clone GK1.5) (n=5). Error bars depict mean with SEM. A two-way ANOVA was performed using Dunnett's multiple comparisons test. At day 50, when compared to adjuvant alone, significant protection was provided by treatment with both EO771 WCV ($p<0.0001$) as well as EO771 WCV +CD4 depletion ($p<0.0001$).

Figure S2. Uncropped western blot images and densitometry from Figure 2c.

Zeb1

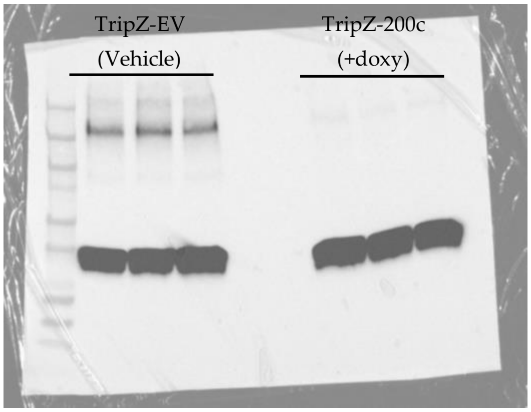

GAPDH

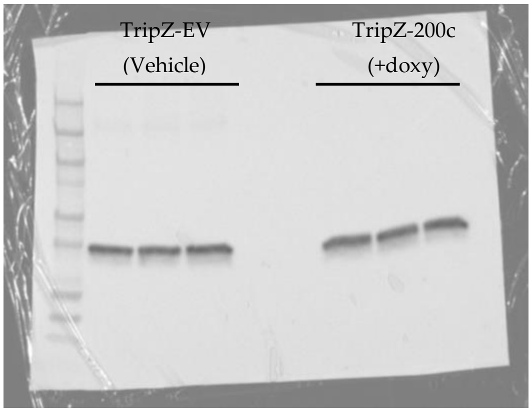

Densitometry

Zeb1			GAPDH		
	Mean pixel	Pixel area		Mean pixel	Pixel area
Spot 1	1407.42	12728	Spot 1	16451.58	16848
Spot 2	1523.83	12728	Spot 2	16705.06	16848
Spot 3	1354.71	12728	Spot 3	19561.31	16848
Spot 4	218.33	12728	Spot 4	20008.52	16848
Spot 5	190.24	12728	Spot 5	18765.15	16848
Spot 6	197.94	12728	Spot 6	18775.73	16848
Background	96.19	12728	Background	336.06	16848

Zeb1

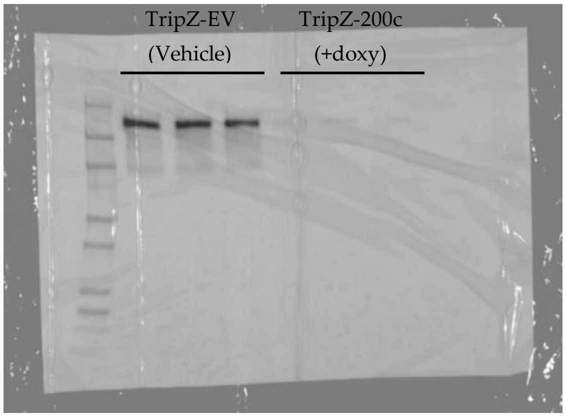

B-actin (shorter exposure)

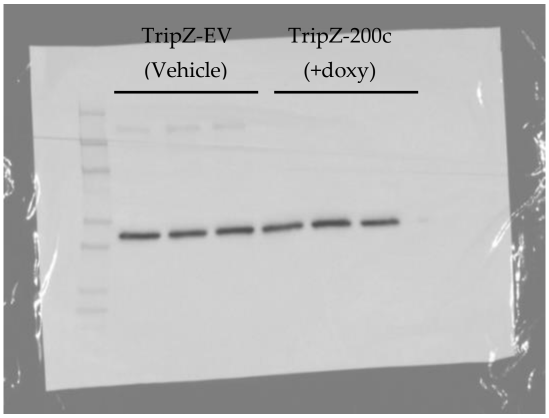

Zeb1				B-actin	
	Mean pixel	Pixel area		Mean pixel	Pixel area
Spot 1	19218.37	9450	Spot 1	25058.26	7350
Spot 2	17998.71	9450	Spot 2	22782.59	7350
Spot 3	16304.25	9450	Spot 3	25193.4	7350
Spot 4	1531.76	9450	Spot 4	23097.39	7350
Spot 5	1789.48	9450	Spot 5	26547.88	7350
Spot 6	1399.07	9450	Spot 6	20689.65	7350
Background	596.32	9450	Background	605.71	7350

Figure S3. Gating strategy to identify proliferating CD8+ T cell population from Fig. 4b flow cytometry analysis.

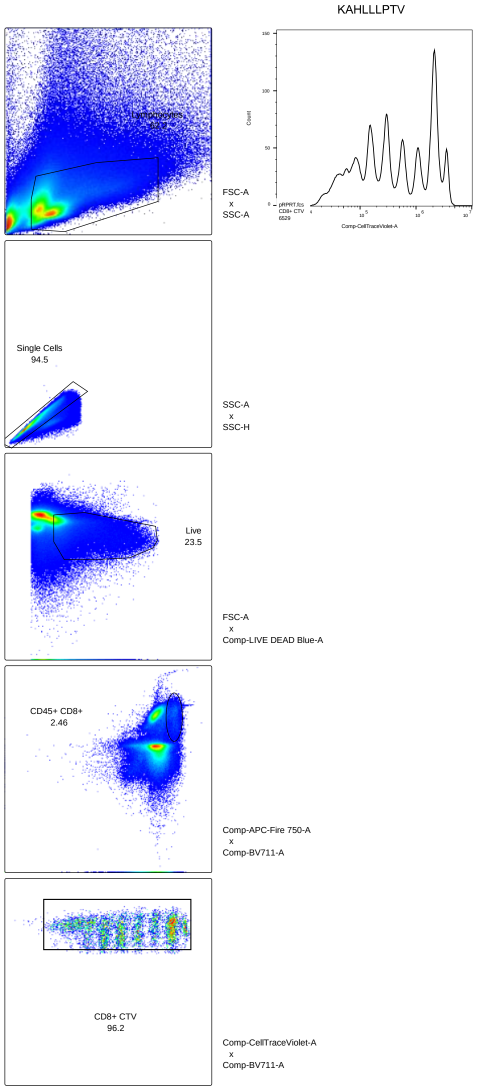

Supplement: Supplementary file 1 [file cancers-14-04397-s001.zip › cancers-1873714-supplementary-Figures.pdf]
