# Supplementary material for: Antigens Expressed by Breast Cancer Cells Undergoing EMT Stimulate Cytotoxic CD8+ T Cell Immunity
Source: Cancers (Basel). 2022 Sep 9;14(18):4397. doi: 10.3390/cancers14184397 (PMC9496737; doi:10.3390/cancers14184397)
Supplement: Supplementary file 1 [file cancers-14-04397-s001.zip › cancers-1873714-supplementary-Tables.pdf]

cancers MDPI

Table S1. Primers for qRT-PCR.

Target	Forward	Reverse
Qki	GAACTCCGCCGCCAAG	GCCTCTATTTTGCTCCTCCTTCT
ESPR1	CTTCCAAGGTTACCAGTATGCAA	TTGTTGTAAGGCTTTGCTGGGTT
RMB47 Isoform 1, 4	GGAAGAGCAGGGATTGCAGA	CTCTGAGGCAAACCCGAACT
RMB47 Isoform 2, 3	AACAGGCTTAGCGCTCCATT	CTCTGAGGCAAACCCGAACT
ESPR2	TAGAGGTGGCCCGTTTCCTA	CATAGCGGTTAAGGACCTGCT
NOVA2	CCAAAGACTTCTACCCAGGAAC	GTCGGGGTTCATCGTGGTTT
SRSF6	GCGTCTACATAGGACGCCTG	TCCACGAAACCGTACCCATT
CDH1 (mine)	ATGATGCCCCCAACACTCC	TGGCAATGGGTGAACCATCA
Cdh2	GCTTCGTGACACCCCTCAC	ACAGACGCCTGAAGCAAGG
ZEB1 (mine)	GCGGCGCAATAACGTTACAA	TCATGACTGCTGGCTTCTGG
Vim	GGCTGCGAGAGAAATTGCAG	CGATCTGGACATGCTGTTCCT

Table S2. Primers for intron retention events.

Target	Forward	Reverse
Macrod1	AAGGACGAGGGCATTTACCG	CCTGTTCAGGGTGGGATGTG
Dvl1	CAGCATCACAGACTCCACCAT	ATCTCCCGAAGCACCCGTA
Rhod	GCTGCAAGATAGACCTGCGT	ATTCTGAGTAATCCGCCGCC
Kmt2d	CCTCTTTGCTCCCTTTGGCA	TCGGCGGGTTACTCAGGTTA
Ocel	GTACCCACCAGTGACCAACA	CCCTCGCTTCTTCTCAAACTCT
Rabggta	CTCCAAGATGGGGACCTTGC	CTGTTCATCAGTGGCTGAGTC
Trim11	GACATTAAGGACGCCCTGTG	TCCCACGTGTGGTCAGAAAG
Inf2	CCTCCTGGTATGTGGATGCC	TCACACACAGCCTTTTTGCCT
Tex9	GACTCCACTCTGCAGATAAAGAA	TTGGCAGTCTGCGCATC
Dennd3	AGTGGTCGTGTTTGATGCCT	GGAGAACGGTGATCCGTGAG
Pan2	CGTCCACCCTACTCTTCACAC	CTCCAGGCAGATACTTCGCT
Rfng (4/5)	ACGAAAATGGTTCTGCCACG	CGGGCTCATTTTGAGGGCAA
(5/6)	TCTGGTTTGCTACTGGTGGG	GCAAAATAGCACCAGACGGC
Gpt (8/9)	GGGTTTCGTGGTGGCTATGT	GAAGACCTGCTCCGTGAGTT
(9/10)	CTTCCCTCAAATTCAGCTGCC	AATGATAGGTGCCCTCCTGCT
Ydjc	AACTAGAAGCTCAACTCAGCCG	CAGCCGTGTGAAGCGGA
Usp21	TTCTGGGCTCTGGTCATGTTG	AAGTCCCTTCGCAGACAGAAG
Ampd2	GCCTGTTCCTCAGCTACCAC	TCATGAGCACGCTGTTACGG
Cchcr1	TTCGTGGTCGGTGCTATGAAC	CATAGCTGAGTCGGTTGCTGA
Ddx39b	GCAAAGAGATCCGCCCAGTC	TGGTCTCGTCATCCACGAAG
Gtpbp2	TGAACCCATCTCAGTACCGC	CATAATCCACCTCTCGCTCCC
Npepl1	GACGGGGTGTCTTACGCTTG	TCAGGGCAGTAGACTAGCGG

Table S3. Differential gene expression in EO771 cells following miR200c restoration (p.adj. < 0.05, log2FC > 1.2).

Genes	log2FC	AveExpr	t	*p*-Value	adj.P.Val	B
Slco2b1	−1.58	0.27	−14.40	0.00	0.000	10.26
Gbp3	−1.56	−1.40	−11.49	0.00	0.000	8.33
Mmp2	−1.80	−1.86	−12.58	0.00	0.000	9.12
Fam214a	−1.77	0.22	−11.19	0.00	0.000	8.15
Mndal	−1.70	−1.17	−13.42	0.00	0.000	9.66
Aldh1l1	−1.68	1.19	−12.51	0.00	0.000	9.47
Rtp4	−1.59	−1.09	−12.24	0.00	0.000	8.91
Ifi203	−1.41	−1.42	−10.71	0.00	0.000	7.74
Aspa	−1.40	−1.27	−12.87	0.00	0.000	9.25
Capn6	−1.32	−1.86	−16.57	0.00	0.000	11.00
Mpeg1	−1.97	−1.27	−12.50	0.00	0.000	8.97
Rnase4	−1.84	−1.80	−11.16	0.00	0.000	8.08
Casp12	−1.82	0.31	−10.52	0.00	0.000	7.59
Oas1g	−1.80	−1.95	−1.16	0.00	0.000	6.28
Dlc1	−1.67	0.56	−1.04	0.00	0.000	6.20
Oas2	−1.59	−1.62	−11.75	0.00	0.000	8.48
Gm13373	−1.50	−1.90	−1.69	0.00	0.000	6.79
Oasl2	−1.48	1.31	−15.15	0.00	0.000	11.92
4631405J19Rik	−1.45	−1.04	−1.05	0.00	0.000	5.14
Trim30a	−1.32	−1.17	−10.85	0.00	0.000	7.79
Irgm2	−1.30	−1.13	−1.67	0.00	0.000	5.80
5033428I22Rik	−1.25	−1.28	−1.03	0.00	0.000	6.11
Ifi27l2a	−1.17	−1.46	−1.27	0.00	0.000	4.13
Agt	−1.01	−1.81	−1.69	0.00	0.000	4.67
Ypel4	−1.88	−1.40	−1.81	0.00	0.000	6.92
Slc2a6	−1.88	0.25	−12.70	0.00	0.000	9.34
Ptgds	−1.86	2.21	−18.64	0.00	0.000	14.72
Selenop	−1.82	1.31	−12.82	0.00	0.000	10.11
Thrsp	−1.80	−1.67	−1.97	0.00	0.000	7.02
Prelp	−1.80	1.89	−17.36	0.00	0.000	13.32
Ifit1	−1.78	−1.06	−10.25	0.00	0.000	7.57
Sema6d	−1.65	−1.03	−1.66	0.00	0.000	4.59
Xaf1	−1.46	−1.64	−11.75	0.00	0.000	8.82
Plxnb3	−1.44	−1.05	−1.86	0.00	0.000	3.61
Fmo1	−1.35	0.68	−14.97	0.00	0.000	11.39
Igtp	−1.34	−1.11	−1.93	0.00	0.000	3.70
Aldh3a1	−1.30	−1.71	−1.11	0.00	0.000	6.32
Gprc5b	−1.25	−1.97	−1.77	0.00	0.000	3.48
Ctso	−1.23	1.16	−11.79	0.00	0.000	9.27
Pla2g4a	−1.23	−1.32	−1.31	0.00	0.000	4.18
Layn	−1.19	0.44	−10.04	0.00	0.000	7.29
Ypel2	−1.17	0.95	−11.40	0.00	0.000	8.81
Cd69	−1.16	−1.98	−1.34	0.00	0.000	4.18
Nsd1	−1.13	1.78	−17.10	0.00	0.000	13.21
Adgra2	−1.11	1.79	−14.84	0.00	0.000	11.71
Pbx1	−1.03	−1.74	−1.51	0.00	0.000	4.43
Egr1	−1.00	1.08	−12.34	0.00	0.000	9.99
Ifi44	−1.98	0.04	−13.22	0.00	0.000	10.33

Oas1b	−1.97	−1.91	−1.80	0.00	0.000	2.08
Sall2	−1.97	0.32	−10.57	0.00	0.000	7.99
Map2k6	−1.95	0.61	−1.38	0.00	0.000	6.80
Ifi207	−1.90	−1.43	−1.23	0.00	0.000	2.72
C1ra	−1.88	3.00	−17.85	0.00	0.000	14.45
Irf7	−1.83	0.30	−11.54	0.00	0.000	9.24
Gab3	−1.80	0.46	−11.45	0.00	0.000	8.73
Acrbp	−1.79	−1.79	−1.03	0.00	0.001	0.88
Tle2	−1.76	3.69	−10.24	0.00	0.000	16.19
Slfn2	−1.75	0.15	−1.97	0.00	0.000	5.15
Prickle2	−1.60	−1.88	−1.35	0.00	0.000	6.41
Pik3ip1	−1.54	1.69	−15.44	0.00	0.000	12.43
Ppargc1a	−1.47	−1.09	−1.45	0.00	0.000	4.45
Plscr4	−1.41	0.05	−1.13	0.00	0.000	5.38
Zfr2	−1.40	1.59	−12.85	0.00	0.000	10.47
Prtn3	−1.39	3.26	−18.58	0.00	0.000	15.14
S100a7a	−1.38	−1.70	−1.29	0.00	0.000	4.19
Col13a1	−1.37	0.13	−10.80	0.00	0.000	8.18
Apol9a	−1.37	1.51	−10.19	0.00	0.000	8.06
Fabp4	−1.36	0.78	−10.05	0.00	0.000	7.74
Sgk3	−1.35	1.10	−11.11	0.00	0.000	8.77
Fbxo41	−1.33	0.72	−1.13	0.00	0.000	5.45
Fkbp7	−1.30	0.71	−10.55	0.00	0.000	8.08
2900041M22Rik	−1.26	0.27	−1.40	0.00	0.000	5.77
Casp4	−1.24	2.88	−14.14	0.00	0.000	11.84
Oas1a	−1.22	1.72	−12.90	0.00	0.000	10.64
Kifc2	−1.20	−1.13	−1.39	0.00	0.002	-0.24
Cetn4	−1.19	0.49	−1.17	0.00	0.000	5.47
Calml4	−1.17	0.68	−10.06	0.00	0.000	7.74
1810010H24Rik	−1.14	1.46	−12.82	0.00	0.000	10.43
Gm14027	−1.10	−1.02	−1.50	0.00	0.001	0.00
Isg15	−1.09	0.97	−10.45	0.00	0.000	8.30
Esr1	−1.08	1.39	−11.27	0.00	0.000	9.01
Fndc5	−1.08	−1.76	−1.96	0.00	0.000	3.84
Cpeb1	−1.05	1.12	−10.31	0.00	0.000	7.96
Lbp	−1.04	2.55	−15.60	0.00	0.000	13.09
Rsad2	−1.01	1.32	−13.26	0.00	0.000	10.91
Ifit3	−1.98	−1.41	−1.82	0.00	0.000	7.24
Sema6c	−1.96	−1.79	−1.16	0.00	0.000	5.34
Vwa5b1	−1.96	−1.01	−1.58	0.00	0.000	5.86
Rassf2	−1.95	−1.47	−1.28	0.00	0.000	5.50
Slfn8	−1.94	0.09	−1.14	0.00	0.000	2.67
Lrp1	−1.93	7.91	−11.87	0.00	0.000	22.43
Padi3	−1.89	0.73	−1.50	0.00	0.000	7.26
Hopx	−1.87	0.59	−10.31	0.00	0.000	7.88
Abcg2	−1.85	5.19	−16.69	0.00	0.000	19.98
Btnl7-ps	−1.85	−1.70	−1.16	0.00	0.000	6.66
Apol9b	−1.84	1.50	−15.34	0.00	0.000	12.72
Clec1a	−1.83	−1.22	−1.92	0.00	0.000	3.82
Ccl5	−1.83	−1.58	−1.96	0.00	0.000	3.84
Pde4d	−1.81	1.12	−1.53	0.00	0.000	7.18

Enpp2	−1.81	0.64	−1.55	0.00	0.000	7.16
Gm7967	−1.80	0.47	−1.67	0.00	0.000	6.18
Gm29216	−1.79	5.95	−1.44	0.00	0.000	6.86
Sema4g	−1.79	0.65	−10.48	0.00	0.000	8.29
Lrrc32	−1.78	−1.11	−1.16	0.00	0.000	6.65
mt-Tt	−1.78	−1.46	−1.24	0.00	0.002	-0.46
C1s1	−1.77	3.93	−19.78	0.00	0.000	16.16
Cd68	−1.73	5.24	−14.59	0.00	0.000	19.00
Nppb	−1.70	0.84	−1.21	0.00	0.000	5.58
Podnl1	−1.68	0.18	−1.25	0.00	0.000	4.30
Casp1	−1.66	0.03	−1.57	0.00	0.000	4.69
Dglucy	−1.65	0.59	−1.87	0.00	0.000	6.37
Ddx58	−1.64	3.78	−18.32	0.00	0.000	15.23
Adamts6	−1.59	1.79	−10.36	0.00	0.000	8.23
Izumo4	−1.56	0.39	−1.19	0.00	0.000	4.24
Coq8a	−1.55	3.39	−17.32	0.00	0.000	14.49
Prrt1	−1.55	0.15	−1.96	0.00	0.000	6.41
Tcp11l2	−1.55	4.14	−18.97	0.00	0.000	15.70
Fxyd1	−1.52	0.94	−1.17	0.00	0.000	5.60
mt-Co1	−1.52	11.62	−1.20	0.00	0.000	3.42
Nr1h4	−1.51	−1.25	−1.99	0.00	0.000	3.88
Gm13340	−1.50	4.62	−1.71	0.00	0.000	4.66
Mr1	−1.49	5.32	−14.64	0.00	0.000	19.06
Gm36738	−1.49	−1.43	−1.95	0.00	0.001	0.73
Clec2d	−1.48	0.88	−10.24	0.00	0.000	7.93
Ccl2	−1.48	3.24	−16.08	0.00	0.000	13.58
9930017N22Rik	−1.46	0.76	−1.58	0.00	0.000	1.77
Cnrip1	−1.45	2.74	−16.59	0.00	0.000	13.73
Calcrl	−1.43	0.20	−1.02	0.00	0.000	6.46
Zc4h2	−1.43	1.40	−10.97	0.00	0.000	8.81
Gm43414	−1.43	0.71	−1.64	0.00	0.000	3.43
Ccl7	−1.43	0.45	−1.48	0.00	0.000	4.64
Sept4	−1.41	3.44	−17.69	0.00	0.000	14.79
mt-Tw	−1.39	0.33	−1.74	0.00	0.000	2.05
Oas3	−1.38	0.54	−12.04	0.00	0.000	9.93
Arhgap25	−1.33	0.15	−1.19	0.00	0.000	4.20
Sp100	−1.31	2.67	−14.44	0.00	0.000	12.25
Clcnka	−1.30	−1.56	−1.58	0.00	0.000	1.79
Gm20342	−1.30	0.52	−1.84	0.00	0.001	0.51
Wfdc1	−1.29	4.23	−10.87	0.00	0.000	16.90
Epor	−1.29	0.33	−1.06	0.00	0.000	4.07
Ing4	−1.28	−1.45	−1.82	0.00	0.004	-1.26
Psd2	−1.28	1.82	−1.74	0.00	0.000	6.35
Hebp2	−1.26	0.53	−1.61	0.00	0.000	6.09
BC028528	−1.25	2.27	−11.49	0.00	0.000	9.51
Gm38157	−1.25	−1.33	−1.37	0.00	0.002	-0.24
Sat2	−1.24	1.08	−1.20	0.00	0.000	6.86
Prr5	−1.23	1.94	−12.82	0.00	0.000	10.74
Napsa	−1.21	3.52	−15.63	0.00	0.000	13.27
Ssu2	−1.20	0.21	−1.79	0.00	0.000	3.66
Mkln1os	−1.20	−1.19	−1.99	0.00	0.000	2.44

Gm8899	−1.19	−1.57	−1.58	0.00	0.000	1.78
Gm35019	−1.17	0.37	−1.01	0.00	0.001	0.83
Tmem191c	−1.17	0.33	−1.06	0.00	0.000	5.42
Poln	−1.16	−1.04	−1.55	0.00	0.000	4.68
Fas	−1.15	0.24	−1.25	0.00	0.000	4.31
Edn2	−1.14	1.16	−1.60	0.00	0.000	7.39
Adh1	−1.12	−1.52	−1.58	0.00	0.000	3.31
Mir142hg	−1.12	−1.67	−1.25	0.00	0.002	-0.44
Fbxl21	−1.08	0.42	−1.93	0.00	0.000	6.48
1110046J04Rik	−1.08	1.25	−10.28	0.00	0.000	8.07
Ifi209	−1.08	0.90	−1.02	0.00	0.000	5.37
Gm37261	−1.08	−1.34	−1.50	0.00	0.001	-0.04
Gm42636	−1.07	−1.38	−1.88	0.00	0.004	-1.12
Fmo5	−1.06	−1.35	−1.91	0.00	0.000	3.82
Cmpk2	−1.05	−1.61	−1.63	0.00	0.000	3.42
Tceal1	−1.05	0.48	−1.95	0.00	0.000	5.28
Gstm6	−1.04	1.22	−1.63	0.00	0.000	6.19
Isoc2b	−1.04	1.53	−11.60	0.00	0.000	9.55
Dhx58	−1.04	2.36	−10.94	0.00	0.000	8.94
Plekha4	−1.00	0.26	−1.08	0.00	0.000	6.65
Glipr1	−1.00	3.98	−15.88	0.00	0.000	13.50
Parp14	−1.00	2.02	−13.40	0.00	0.000	11.35
Col2a1	−1.00	2.99	−1.30	0.00	0.000	5.71
Rnf207	−1.00	−1.04	−1.99	0.00	0.000	6.50
Mir7688	−1.99	0.09	−1.55	0.00	0.001	0.03
H2-K2	−1.97	1.86	−1.88	0.00	0.000	5.21
Apobec1	−1.97	2.65	−11.41	0.00	0.000	9.43
Ndufa4l2	−1.95	−1.19	−1.96	0.00	0.000	2.39
1700001J03Rik	−1.92	−1.62	−1.86	0.01	0.022	-2.94
Lnx1	−1.92	1.34	−1.01	0.00	0.000	6.67
Sept12	−1.91	−1.28	−1.24	0.00	0.000	1.23
Chrd	−1.90	2.20	−13.48	0.00	0.000	11.36
Lamb2	−1.89	6.77	−17.01	0.00	0.000	20.31
Mir5125	−1.89	2.36	−1.47	0.00	0.000	4.61
Gm28578	−1.88	0.90	−1.58	0.00	0.000	1.72
Nod1	−1.87	2.71	−12.24	0.00	0.000	10.28
Prdm11	−1.86	−1.16	−1.99	0.00	0.000	3.95
Zscan10	−1.86	0.27	−1.48	0.00	0.000	4.65
4930550C14Rik	−1.85	−1.30	−1.15	0.00	0.000	4.12
Gstt1	−1.85	5.88	−17.72	0.00	0.000	20.62
Mfsd7a	−1.85	0.85	−1.75	0.00	0.000	3.61
Slc6a4	−1.85	−1.24	−1.68	0.00	0.000	4.90
Gm28661	−1.84	5.98	−1.33	0.00	0.000	2.21
mt-Co3	−1.83	5.64	−1.47	0.00	0.000	2.53
Gstk1	−1.82	0.49	−1.30	0.00	0.000	1.31
Ebf4	−1.81	1.31	−11.62	0.00	0.000	9.53
Ankrd44	−1.81	2.80	−13.54	0.00	0.000	11.48
Psmb9	−1.81	2.29	−11.34	0.00	0.000	9.35
Serpinb8	−1.80	0.21	−1.94	0.00	0.000	3.88
Gm5751	−1.80	0.66	−1.44	0.00	0.000	1.50
Cbr1	−1.80	−1.06	−1.89	0.00	0.000	2.28

Dlg4	−1.79	4.11	−19.89	0.00	0.000	16.30
Sept1	−1.79	−1.26	−1.71	0.00	0.001	0.33
Ncf4	−1.78	1.03	−1.70	0.00	0.000	6.28
Ggt7	−1.78	0.49	−1.23	0.00	0.000	5.65
Smarca2	−1.77	0.76	−1.73	0.00	0.000	5.00
Icam1	−1.77	2.97	−14.43	0.00	0.000	12.30
Gm49492	−1.77	−1.33	−1.32	0.00	0.000	2.94
Tcn2	−1.76	4.36	−19.80	0.00	0.000	16.29
AC079441.1	−1.76	−1.02	−1.05	0.00	0.000	2.53
Enox1	−1.75	0.53	−1.70	0.00	0.000	3.53
Mmp28	−1.74	3.24	−16.90	0.00	0.000	14.23
Pde5a	−1.74	0.29	−1.77	0.00	0.000	5.02
AC122252.2	−1.73	2.52	−1.90	0.00	0.000	2.07
Ppm1k	−1.73	3.71	−12.41	0.00	0.000	10.44
Ablim3	−1.72	1.35	−1.71	0.00	0.000	4.98
Macrod1	−1.72	4.65	−17.94	0.00	0.000	15.05
Wnk4	−1.72	2.76	−12.24	0.00	0.000	10.28
Stard9	−1.71	2.32	−1.03	0.00	0.000	5.36
Tmem150a	−1.69	3.91	−14.41	0.00	0.000	12.29
Malat1	−1.68	7.78	−1.19	0.00	0.002	-1.86
Fcgrt	−1.68	4.99	−13.08	0.00	0.000	18.26
Ccdc17	−1.68	−1.13	−1.01	0.00	0.001	0.81
mt-Co2	−1.68	6.20	−1.22	0.00	0.000	1.98
Cxcl10	−1.68	−1.12	−1.03	0.00	0.000	4.00
Foxl1	−1.68	1.74	−1.02	0.00	0.000	6.70
Uba7	−1.68	−1.09	−1.65	0.00	0.000	4.88
Klf8	−1.67	1.86	−1.45	0.00	0.000	4.60
Padi2	−1.67	0.59	−1.06	0.00	0.000	5.44
9330102E08Rik	−1.66	1.88	−11.67	0.00	0.000	9.64
1700109H08Rik	−1.66	−1.81	−1.59	0.02	0.035	-3.38
3110039I08Rik	−1.66	1.73	−1.79	0.00	0.000	3.62
Zfp607b	−1.65	−1.26	−1.17	0.00	0.000	1.09
Gm28437	−1.64	5.62	−1.87	0.00	0.000	3.16
Slc9a9	−1.63	1.71	−11.28	0.00	0.000	9.26
Hspa12b	−1.62	3.36	−15.94	0.00	0.000	13.53
Lipa	−1.62	4.63	−11.69	0.00	0.000	17.44
Lhfp	−1.62	5.04	−18.48	0.00	0.000	15.43
Myh7b	−1.62	0.20	−1.64	0.00	0.001	0.14
Ankrd24	−1.61	4.15	−13.40	0.00	0.000	11.37
Tmem71	−1.61	2.12	−10.24	0.00	0.000	8.16
Pde4c	−1.60	0.32	−1.78	0.00	0.001	0.41
Gm45494	−1.60	−1.24	−1.73	0.00	0.000	2.02
Zcchc24	−1.60	6.32	−19.59	0.00	0.000	21.46
Mgll	−1.60	−1.36	−1.80	0.00	0.000	2.13
Ndrg4	−1.59	3.23	−11.51	0.00	0.000	9.55
H2-DMa	−1.59	3.31	−14.06	0.00	0.000	11.98
Ifih1	−1.59	1.93	−11.99	0.00	0.000	10.00
Mid1ip1	−1.59	0.23	−1.06	0.00	0.001	0.89
Ifi211	−1.59	2.07	−10.41	0.00	0.000	8.34
Meiob	−1.58	0.10	−1.52	0.00	0.000	4.66
Mapk4	−1.57	−1.12	−1.77	0.00	0.000	5.03

Stac3	−1.57	2.08	−11.96	0.00	0.000	9.97
Kdm7a	−1.57	5.45	−16.06	0.00	0.000	13.61
Acyp1	−1.57	3.75	−11.27	0.00	0.000	9.25
Tfr2	−1.56	0.01	−1.30	0.00	0.000	2.92
Ifi27	−1.56	5.04	−12.46	0.00	0.000	17.92
Lpin1	−1.55	3.96	−14.03	0.00	0.000	11.96
Ahnak2	−1.55	6.23	−15.83	0.00	0.000	13.35
Tmem140	−1.55	1.00	−1.96	0.00	0.000	6.64
Nos3	−1.54	−1.37	−1.19	0.00	0.000	1.14
Ifi204	−1.54	2.25	−1.81	0.00	0.000	7.67
Vwf	−1.54	0.26	−1.71	0.00	0.000	6.26
Lipn	−1.53	0.17	−1.44	0.00	0.000	1.53
Slc25a27	−1.53	2.67	−1.07	0.00	0.000	5.42
Naglu	−1.53	4.85	−16.87	0.00	0.000	14.26
Hacd4	−1.52	0.11	−1.07	0.00	0.000	4.07
Irf9	−1.52	3.70	−18.77	0.00	0.000	15.60
Gm15832	−1.52	−1.02	−1.14	0.00	0.001	1.05
Arhgap18	−1.51	5.84	−12.89	0.00	0.000	18.17
Xkr8	−1.51	2.37	−1.79	0.00	0.000	7.65
Slc5a5	−1.51	1.46	−1.56	0.00	0.000	7.36
Pold4	−1.51	4.10	−16.51	0.00	0.000	13.99
Fgd2	−1.50	1.15	−1.78	0.00	0.000	7.61
Azin2	−1.50	2.24	−1.18	0.00	0.000	5.58
Ilvbl	−1.50	4.25	−13.53	0.00	0.000	11.48
Adgrd1	−1.50	0.58	−1.26	0.00	0.000	5.71
Apcdd1	−1.50	2.14	−11.23	0.00	0.000	9.22
Dnm1	−1.50	4.47	−14.66	0.00	0.000	12.48
Loxl3	−1.50	−1.39	−1.04	0.00	0.001	0.88
mt-Atp6	−1.50	6.72	−1.00	0.00	0.000	1.54
Cyp39a1	−1.49	2.40	−11.47	0.00	0.000	9.51
Sesn1	−1.49	4.57	−17.50	0.00	0.000	14.73
Rmdn1	−1.49	1.17	−1.26	0.00	0.000	4.33
Asb2	−1.49	−1.05	−1.28	0.00	0.000	1.24
Des	−1.49	2.11	−10.90	0.00	0.000	8.89
Selenbp1	−1.48	4.17	−14.84	0.00	0.000	12.62
Il2rg	−1.47	0.43	−1.88	0.00	0.000	3.81
1700003E16Rik	−1.46	−1.24	−1.31	0.01	0.010	-2.25
mt-Rnr2	−1.46	8.03	−1.55	0.00	0.000	0.67
Gm43858	−1.45	0.08	−1.49	0.00	0.007	-1.89
1190001M18Rik	−1.45	−1.28	−1.64	0.00	0.000	1.89
Adamts7	−1.45	4.07	−14.61	0.00	0.000	12.46
Inca1	−1.44	0.78	−1.05	0.00	0.000	4.05
Slc28a2	−1.44	0.81	−1.09	0.00	0.000	5.49
Gm10925	−1.43	7.08	−1.55	0.00	0.000	0.72
Haghl	−1.43	2.34	−1.39	0.00	0.000	4.44
Ccno	−1.43	1.72	−1.12	0.00	0.000	6.83
Lyz2	−1.43	3.05	−14.41	0.00	0.000	12.27
Dqx1	−1.42	1.03	−1.46	0.00	0.001	-0.25
Kctd14	−1.42	2.45	−12.94	0.00	0.000	10.94
Ovgp1	−1.41	0.68	−1.86	0.00	0.000	2.22
Plxna3	−1.41	4.31	−10.86	0.00	0.000	8.72

Pramef12	−1.40	2.14	−10.51	0.00	0.000	8.46
Nxph4	−1.39	2.78	−10.72	0.00	0.000	8.69
Aqp1	−1.38	1.41	−1.84	0.00	0.000	6.47
Cdc42ep5	−1.38	3.56	−11.67	0.00	0.000	9.67
Sp7	−1.37	−1.14	−1.89	0.00	0.000	2.28
Naga	−1.37	5.50	−14.12	0.00	0.000	11.91
Vsir	−1.37	4.90	−12.33	0.00	0.000	17.84
Lhx9	−1.37	2.76	−11.84	0.00	0.000	9.89
Sh2d3c	−1.36	2.21	−1.34	0.00	0.000	7.10
Rab19	−1.36	−1.33	−1.09	0.00	0.003	-0.79
Dcxr	−1.36	2.23	−10.39	0.00	0.000	8.34
Ccdc159	−1.35	1.26	−1.93	0.00	0.000	2.26
Ajm1	−1.35	1.90	−1.43	0.00	0.000	7.21
1700013F07Rik	−1.35	1.69	−1.21	0.00	0.000	5.67
Rgl1	−1.34	3.11	−12.97	0.00	0.000	10.99
Celf5	−1.33	1.13	−1.02	0.00	0.000	5.39
AC154187.1	−1.33	0.42	−1.19	0.00	0.000	2.74
Pla2g16	−1.33	1.00	−1.73	0.00	0.000	5.02
Klhl24	−1.32	4.42	−11.51	0.00	0.000	9.41
Hectd2	−1.32	0.35	−1.20	0.00	0.000	1.15
1700120G11Rik	−1.32	−1.13	−1.16	0.00	0.000	1.09
Cx3cl1	−1.32	1.17	−1.24	0.00	0.000	5.70
Mxd4	−1.32	5.30	−11.05	0.00	0.000	17.09
Gm15867	−1.31	1.65	−1.22	0.00	0.000	6.96
Lif	−1.31	1.53	−1.89	0.00	0.000	3.79
Tjp3	−1.30	−1.11	−1.88	0.00	0.000	3.80
Cabp1	−1.29	3.58	−11.19	0.00	0.000	9.17
1700034P13Rik	−1.29	0.63	−1.37	0.00	0.000	3.02
Mogat2	−1.29	2.81	−12.19	0.00	0.000	10.24
Eno2	−1.29	2.68	−1.59	0.00	0.000	7.39
Foxn3	−1.28	3.62	−13.54	0.00	0.000	11.52
Acta2	−1.28	2.97	−10.34	0.00	0.000	8.27
Rnf122	−1.28	2.04	−1.36	0.00	0.000	7.12
Sept5	−1.28	0.53	−1.48	0.00	0.000	4.66
Mxra8	−1.28	6.68	−11.32	0.00	0.000	17.24
A730063M14Rik	−1.27	−1.07	−1.08	0.00	0.001	0.96
mt-Rnr1	−1.27	7.14	−1.27	0.00	0.000	0.20
Rras	−1.27	5.81	−15.84	0.00	0.000	13.38
Bc1	−1.27	7.50	−1.65	0.00	0.000	0.86
Ramp2	−1.26	2.74	−11.03	0.00	0.000	9.04
Zeb2	−1.25	5.38	−11.27	0.00	0.000	9.06
AI413582	−1.24	5.47	−14.88	0.00	0.000	12.59
Mertk	−1.24	3.72	−10.20	0.00	0.000	8.00
Nxf3	−1.24	6.12	−11.38	0.00	0.000	9.08
Mill2	−1.24	0.78	−1.58	0.00	0.000	3.34
Nnmt	−1.23	1.80	−1.63	0.00	0.000	4.87
Ddb2	−1.23	3.24	−10.98	0.00	0.000	8.96
Pcmtd1	−1.22	4.97	−14.01	0.00	0.000	11.86
Rdh5	−1.22	1.18	−1.15	0.00	0.000	2.64
4933430I17Rik	−1.22	−1.07	−1.10	0.00	0.000	4.12
Stat2	−1.22	4.12	−17.33	0.00	0.000	14.61

Cdh13	−1.22	6.54	−12.16	0.00	0.000	17.75
4933412E12Rik	−1.21	0.36	−1.77	0.00	0.000	3.63
Frat2	−1.21	2.46	−12.68	0.00	0.000	10.70
Fos	−1.21	0.67	−1.51	0.00	0.000	1.59
Gm5930	−1.21	2.13	−1.04	0.00	0.000	6.73
Entpd7	−1.21	4.78	−11.47	0.00	0.000	9.34
Nod2	−1.20	−1.05	−1.98	0.00	0.001	0.78
Usp44	−1.20	−1.08	−1.47	0.00	0.000	1.61
Mmd	−1.19	1.48	−1.41	0.00	0.000	4.55
Dtx3	−1.19	6.18	−15.24	0.00	0.000	12.84
Syde1	−1.19	5.80	−10.38	0.00	0.000	16.67
Fhl4	−1.19	0.27	−1.30	0.00	0.000	2.92
1810021B22Rik	−1.19	−1.06	−1.27	0.00	0.000	2.88
Vamp5	−1.18	3.41	−13.05	0.00	0.000	11.06
Trim12c	−1.18	0.01	−1.18	0.00	0.000	4.24
Bex2	−1.18	−1.40	−1.02	0.01	0.017	-2.71
H2-M3	−1.17	0.78	−1.63	0.00	0.000	1.83
Adamtsl4	−1.17	3.50	−11.05	0.00	0.000	9.02
Itga7	−1.17	2.97	−12.23	0.00	0.000	10.28
Ptpn22	−1.16	0.75	−1.85	0.00	0.000	2.14
Plekhg6	−1.16	0.39	−1.12	0.00	0.000	2.64
Fhod1	−1.16	5.60	−10.19	0.00	0.000	16.55
Gm10457	−1.16	−1.13	−1.15	0.00	0.002	-0.70
Eva1b	−1.16	5.11	−15.56	0.00	0.000	13.17
Samd9l	−1.15	4.42	−19.29	0.00	0.000	15.97
Ube2l6	−1.15	1.26	−1.35	0.00	0.000	4.46
Gm17491	−1.15	2.14	−1.38	0.00	0.000	1.21
Ripk2	−1.15	3.85	−11.78	0.00	0.000	9.78
Me3	−1.15	2.49	−10.97	0.00	0.000	8.97
Dbn1	−1.15	3.72	−12.91	0.00	0.000	10.92
H2-K1	−1.15	6.97	−17.75	0.00	0.000	14.81
Catsperg1	−1.14	0.45	−1.92	0.00	0.001	0.63
Rn7sk	−1.14	1.96	−1.78	0.00	0.001	0.18
Bach2	−1.14	0.20	−1.76	0.00	0.000	3.62
Gm37499	−1.14	0.54	−1.61	0.00	0.000	1.81
Rnasel	−1.14	3.04	−1.34	0.00	0.000	7.02
Fam193b	−1.14	5.96	−12.33	0.00	0.000	10.12
Dennd5a	−1.14	6.53	−10.47	0.00	0.000	16.71
Nckap5l	−1.13	3.85	−15.15	0.00	0.000	12.92
Acsl6	−1.13	5.20	−18.18	0.00	0.000	15.21
Ppm1j	−1.13	3.53	−14.10	0.00	0.000	12.03
Rorc	−1.12	4.35	−18.22	0.00	0.000	15.25
Vstm5	−1.12	0.67	−1.09	0.00	0.001	0.91
Atp8b3	−1.12	0.08	−1.99	0.00	0.000	3.95
Aldh6a1	−1.12	3.43	−1.73	0.00	0.000	7.46
4930539E08Rik	−1.11	1.60	−1.34	0.00	0.000	5.84
Slc7a7	−1.11	3.63	−13.82	0.00	0.000	11.77
Tet1	−1.11	0.17	−1.28	0.00	0.000	1.25
Usp35	−1.11	2.18	−1.56	0.00	0.000	7.36
G730013B05Rik	−1.11	4.73	−17.49	0.00	0.000	14.72
Fam49a	−1.10	3.15	−13.27	0.00	0.000	11.28

Lrrc74b	−1.10	−1.11	−1.43	0.00	0.000	3.11
4933439C10Rik	−1.10	1.44	−1.61	0.00	0.000	1.74
Trim21	−1.09	1.08	−1.55	0.00	0.000	4.74
Gbp2	−1.09	−1.39	−1.92	0.00	0.001	0.63
Arap3	−1.09	4.00	−14.13	0.00	0.000	12.03
Csad	−1.09	2.90	−11.19	0.00	0.000	9.20
C230037L18Rik	−1.09	0.57	−1.28	0.00	0.000	1.24
Pdk2	−1.09	3.50	−13.26	0.00	0.000	11.26
Gm13341	−1.08	0.63	−1.74	0.00	0.005	-1.53
Gm15421	−1.07	0.27	−1.03	0.00	0.001	0.84
Znrf1	−1.07	5.90	−15.90	0.00	0.000	13.42
Stat1	−1.07	3.48	−14.99	0.00	0.000	12.78
Nptx1	−1.07	6.10	−10.07	0.00	0.000	16.46
Arl4d	−1.07	2.41	−1.31	0.00	0.000	5.75
Zfp30	−1.06	2.46	−10.95	0.00	0.000	8.95
Tsga10	−1.06	0.58	−1.91	0.00	0.000	2.27
Cyp27a1	−1.05	4.59	−12.66	0.00	0.000	10.59
Vdr	−1.05	2.66	−1.62	0.00	0.000	7.40
mt-Cytb	−1.05	10.61	−1.53	0.00	0.001	-1.25
Gimap6	−1.04	0.99	−1.87	0.00	0.000	2.19
Gm46404	−1.04	1.45	−1.49	0.00	0.000	4.67
Zeb1	−1.04	5.25	−14.09	0.00	0.000	11.91
C2	−1.04	3.06	−11.07	0.00	0.000	9.05
Ccn5	−1.03	2.28	−11.03	0.00	0.000	9.02
Thra	−1.03	5.89	−10.95	0.00	0.000	17.03
Ppp1r12b	−1.02	4.54	−12.05	0.00	0.000	9.98
E130307A14Rik	−1.02	0.87	−1.32	0.00	0.000	1.27
Sele	−1.02	0.57	−1.55	0.00	0.000	3.29
Slc25a28	−1.02	6.71	−13.38	0.00	0.000	11.12
Zfp14	−1.01	0.54	−1.60	0.00	0.000	3.37
Alx3	−1.01	−1.12	−1.86	0.00	0.004	-1.24
Akap7	−1.01	4.71	−15.41	0.00	0.000	13.10
Stau2	−1.01	3.27	−11.46	0.00	0.000	9.47
Papln	−1.01	−1.25	−1.29	0.00	0.002	-0.49
Flot1	−1.01	7.21	−12.50	0.00	0.000	17.93
H3f3aos	−1.01	0.36	−1.57	0.00	0.000	1.74
Cbr2	−1.01	3.96	−15.03	0.00	0.000	12.81
Phf1	−1.01	5.15	−17.70	0.00	0.000	14.85
Gm14443	−1.01	−1.16	−1.18	0.00	0.002	-0.68
Rwdd3	−1.01	2.19	−1.82	0.00	0.000	5.07
Hnrnph3	−1.00	4.94	−13.45	0.00	0.000	11.35
Izumo1	−1.00	1.17	−1.68	0.00	0.000	1.85
Lims2	−1.00	4.39	−13.78	0.00	0.000	11.68
Tmod1	−1.00	2.96	−12.15	0.00	0.000	10.19
Crebrf	−1.99	4.50	−12.69	0.00	0.000	10.63
Hyi	−1.99	4.04	−10.31	0.00	0.000	8.09
Msn	−1.99	9.06	−11.55	0.00	0.000	17.35
Gm14267	−1.99	1.40	−1.55	0.00	0.000	6.11
Ghdc	−1.99	3.82	−14.23	0.00	0.000	12.13
Rasgrp2	−1.99	−1.19	−1.46	0.00	0.000	3.17
Gabbr1	−1.99	2.96	−1.45	0.00	0.000	2.87

Tmem205	−1.99	4.42	−13.55	0.00	0.000	11.47
Slc2a9	−1.98	2.71	−1.69	0.00	0.000	7.49
Il11ra1	−1.98	2.88	−10.86	0.00	0.000	8.83
Tcea3	−1.97	2.05	−1.75	0.00	0.000	6.33
Mical1	−1.97	4.53	−16.30	0.00	0.000	13.82
Metap1d	−1.97	3.15	−1.92	0.00	0.000	7.74
Hoxc8	−1.97	2.21	−1.70	0.00	0.005	-1.89
Lrrc51	−1.97	1.57	−1.69	0.00	0.000	4.94
Gm34248	−1.96	−1.55	−1.87	0.01	0.022	-2.94
Adamts10	−1.96	3.78	−11.27	0.00	0.000	9.21
1700025G04Rik	−1.95	1.96	−1.45	0.00	0.000	4.56
Hexb	−1.95	5.15	−16.33	0.00	0.000	13.81
Mapk8ip3	−1.95	6.59	−1.18	0.00	0.000	4.97
Pdlim4	−1.95	5.99	−13.98	0.00	0.000	11.72
Gm17501	−1.95	0.47	−1.88	0.00	0.000	2.24
Neil1	−1.95	3.27	−10.62	0.00	0.000	8.54
Rapgefl1	−1.95	0.78	−1.76	0.00	0.000	5.05
Fbxo32	−1.94	3.98	−16.79	0.00	0.000	14.21
B230303O12Rik	−1.94	1.92	−1.73	0.00	0.005	-1.77
AC149090.1	−1.94	7.42	−12.75	0.00	0.000	10.45
Gm26885	−1.94	−1.21	−1.37	0.00	0.009	-2.14
Hist1h3c	−1.94	−1.08	−1.21	0.00	0.002	-0.58
Arhgef4	−1.93	1.66	−1.92	0.00	0.000	3.78
Zfp429	−1.93	1.91	−1.17	0.00	0.000	6.90
Ccpg1	−1.92	0.59	−1.17	0.00	0.000	2.70
Appl2	−1.92	5.64	−15.74	0.00	0.000	13.30
Tecpr1	−1.91	6.52	−17.24	0.00	0.000	14.44
Ppox	−1.91	4.73	−10.99	0.00	0.000	8.78
Pou6f1	−1.91	4.05	−11.75	0.00	0.000	9.72
Gna15	−1.90	3.52	−14.02	0.00	0.000	11.94
Ap3b2	−1.90	0.47	−1.82	0.00	0.000	2.16
Cyp4f13	−1.90	4.38	−15.15	0.00	0.000	12.89
Spred3	−1.90	3.80	−1.14	0.00	0.000	6.67
Ppm1f	−1.89	5.14	−17.23	0.00	0.000	14.51
Tlr2	−1.89	2.21	−1.83	0.00	0.000	6.45
Creb3l2	−1.89	6.76	−19.46	0.00	0.000	16.03
Tnfsf12	−1.89	1.33	−1.17	0.00	0.000	4.18
Napepld	−1.89	3.09	−12.13	0.00	0.000	10.18
Zbtb20	−1.89	6.14	−1.96	0.00	0.000	7.39
Msh5	−1.89	1.22	−1.03	0.00	0.000	2.39
Gm16196	−1.89	2.84	−1.81	0.00	0.000	1.83
Gm37123	−1.89	0.83	−1.15	0.00	0.002	-0.78
Vill	−1.88	5.03	−15.22	0.00	0.000	12.89
Polr3gl	−1.88	4.95	−13.64	0.00	0.000	11.49
mt-Nd3	−1.88	3.64	−1.59	0.00	0.001	-0.50
Plcg1	−1.88	5.56	−17.74	0.00	0.000	14.87
Rab6b	−1.88	3.90	−14.12	0.00	0.000	12.02
Angptl6	−1.87	5.09	−12.11	0.00	0.000	9.99
CAAA01141682.1	−1.87	0.69	−1.64	0.00	0.001	0.09
Rps12-ps24	−1.87	0.96	−1.88	0.00	0.001	0.48
Epha8	−1.87	3.30	−13.00	0.00	0.000	11.01

Gypc	−1.86	4.07	−13.17	0.00	0.000	11.14
Cpeb3	−1.86	2.06	−1.43	0.00	0.000	5.91
Gm26664	−1.86	−1.05	−1.16	0.00	0.002	-0.70
Ankrd13d	−1.86	1.07	−1.30	0.00	0.000	2.83
Gstt2	−1.85	1.17	−1.74	0.00	0.000	1.95
2010001A14Rik	−1.85	1.44	−1.46	0.00	0.000	3.08
Cyp2j6	−1.85	3.42	−11.46	0.00	0.000	9.46
Ypel3	−1.85	5.45	−14.71	0.00	0.000	12.43
9530077C05Rik	−1.85	0.96	−1.57	0.00	0.000	4.77
Mx1	−1.85	−1.16	−1.01	0.00	0.001	0.77
Tnfaip3	−1.84	2.98	−1.17	0.00	0.000	4.03
C030015A19Rik	−1.84	1.61	−1.38	0.00	0.000	1.30
Tmem241	−1.84	1.78	−1.89	0.00	0.000	3.73
Kyat3	−1.84	3.09	−10.44	0.00	0.000	8.36
Xlr4b	−1.83	1.67	−1.18	0.00	0.000	0.94
Rpgrip1	−1.83	0.68	−1.16	0.00	0.000	2.65
Ptprn	−1.83	7.17	−17.86	0.00	0.000	14.89
Gm48653	−1.83	0.07	−1.87	0.00	0.004	-1.25
Gm47775	−1.82	0.23	−1.77	0.00	0.001	0.36
Firre	−1.82	3.83	−1.48	0.00	0.000	1.04
Acss2	−1.82	3.64	−13.68	0.00	0.000	11.63
Irf5	−1.82	0.97	−1.41	0.00	0.000	3.06
Vwa5a	−1.82	4.44	−12.03	0.00	0.000	9.93
Lrp8os3	−1.82	−1.04	−1.11	0.00	0.001	0.99
Tle6	−1.81	6.17	−18.88	0.00	0.000	15.64
Gm28438	−1.81	3.51	−1.17	0.00	0.002	-1.26
Mef2b	−1.81	0.04	−1.72	0.00	0.001	0.27
Eps8l1	−1.81	0.78	−1.67	0.00	0.000	4.92
Smim26	−1.81	2.65	−1.56	0.00	0.000	3.12
Gipc3	−1.81	5.10	−14.87	0.00	0.000	12.60
Ttc28	−1.80	3.24	−11.62	0.00	0.000	9.63
Pkd1	−1.80	5.98	−12.35	0.00	0.000	10.13
Tmem256	−1.80	4.47	−1.36	0.00	0.000	5.50
Ldlrad4	−1.80	1.25	−1.74	0.00	0.000	3.56
Iqcg	−1.79	3.80	−12.51	0.00	0.000	10.52
Airn	−1.79	1.37	−1.63	0.00	0.000	1.73
Plat	−1.79	3.76	−1.47	0.00	0.000	5.77
Itga1	−1.79	4.55	−11.92	0.00	0.000	9.84
mt-Atp8	−1.79	3.09	−1.77	0.00	0.005	-1.99
Gpnmb	−1.79	9.35	−10.49	0.00	0.000	16.68
Car11	−1.79	3.53	−10.34	0.00	0.000	8.18
Aspg	−1.78	4.30	−12.81	0.00	0.000	10.76
Prokr1	−1.78	2.55	−1.63	0.00	0.000	7.44
mt-Nd4	−1.78	9.41	−1.84	0.00	0.004	-2.58
Clk1	−1.78	5.91	−1.19	0.00	0.000	5.07
2810403D21Rik	−1.78	2.09	−1.74	0.00	0.000	3.47
Aldh5a1	−1.78	4.15	−12.30	0.00	0.000	10.26
Gm3764	−1.78	0.89	−1.71	0.00	0.000	1.96
Fgfr3	−1.78	0.15	−1.36	0.00	0.002	-0.35
Gm44250	−1.78	5.18	−16.81	0.00	0.000	14.18
Zfp85	−1.78	2.24	−1.06	0.00	0.000	6.73

Kctd21	−1.77	2.47	−1.73	0.00	0.000	6.29
Egr3	−1.77	1.47	−1.75	0.00	0.000	3.52
Adprh	−1.77	5.79	−17.60	0.00	0.000	14.75
Inka1	−1.77	2.17	−1.38	0.00	0.000	5.83
C1qtnf1	−1.77	3.24	−10.71	0.00	0.000	8.60
Grik5	−1.77	0.95	−1.61	0.00	0.000	3.37
Btn2a2	−1.77	0.62	−1.48	0.00	0.001	-0.19
Sh3kbp1	−1.77	3.52	−1.51	0.00	0.000	5.88
D830044D21Rik	−1.77	0.46	−1.64	0.00	0.006	-1.70
Nrbp2	−1.77	3.55	−10.09	0.00	0.000	7.87
Ephx1	−1.77	6.44	−11.56	0.00	0.000	17.38
Gm45833	−1.77	0.81	−1.37	0.00	0.002	-0.38
Hfe	−1.77	3.73	−1.97	0.00	0.000	7.73
Npc1	−1.76	5.82	−16.23	0.00	0.000	13.70
Crb2	−1.76	2.81	−1.49	0.00	0.000	7.23
Kazald1	−1.76	3.58	−1.61	0.00	0.000	7.25
Zfp712	−1.75	1.20	−1.82	0.00	0.001	0.34
Pcbp3	−1.75	1.38	−1.28	0.00	0.000	2.78
Car7	−1.75	−1.12	−1.65	0.00	0.006	-1.63
Mterf2	−1.75	2.58	−1.01	0.00	0.000	6.63
1700003F12Rik	−1.75	1.90	−1.43	0.00	0.000	2.99
Slc2a8	−1.74	4.49	−10.25	0.00	0.000	7.94
Gm34934	−1.74	0.70	−1.18	0.00	0.002	-0.76
Tap1	−1.74	6.15	−16.10	0.00	0.000	13.56
Notch1	−1.74	5.35	−18.13	0.00	0.000	15.15
Lrig1	−1.74	5.84	−16.96	0.00	0.000	14.25
Mmp19	−1.74	4.08	−12.68	0.00	0.000	10.65
Gm44067	−1.73	2.99	−1.07	0.00	0.003	-1.39
Junb	−1.73	6.78	−12.05	0.00	0.000	17.67
Il4ra	−1.73	2.92	−10.88	0.00	0.000	8.85
9130002K18Rik	−1.73	0.01	−1.71	0.02	0.028	-3.36
Zdhhc1	−1.73	4.96	−1.60	0.00	0.000	7.07
Emc9	−1.72	1.50	−1.13	0.00	0.000	2.53
Tmem8b	−1.72	3.28	−10.10	0.00	0.000	7.94
Atg10	−1.72	2.63	−1.82	0.00	0.000	6.40
Wdr45	−1.72	2.06	−1.76	0.00	0.000	4.99
Abcd4	−1.72	5.90	−15.38	0.00	0.000	12.97
Neat1	−1.72	7.10	−1.53	0.00	0.001	-1.20
Gm20756	−1.72	1.85	−1.37	0.00	0.002	-0.62
Oplah	−1.72	4.48	−15.40	0.00	0.000	13.08
Lyst	−1.71	5.72	−10.28	0.00	0.000	7.83
Mrpl24	−1.71	5.18	−14.29	0.00	0.000	12.08
Cd248	−1.71	2.38	−1.53	0.00	0.000	4.58
Cfl2	−1.71	5.17	−12.70	0.00	0.000	10.58
Gm49201	−1.71	0.78	−1.56	0.00	0.000	1.66
L3hypdh	−1.71	4.33	−11.17	0.00	0.000	9.04
Eri3	−1.70	5.27	−15.81	0.00	0.000	13.38
Cd22	−1.70	0.20	−1.09	0.00	0.001	0.91
Doc2g	−1.70	0.78	−1.02	0.00	0.003	-1.06
Gm37893	−1.70	0.42	−1.93	0.01	0.020	-3.02
Mst1	−1.70	5.90	−11.81	0.00	0.000	9.55

Zfas1	−1.70	6.68	−1.99	0.00	0.000	4.66
Dgka	−1.70	4.99	−15.39	0.00	0.000	13.05
Ganc	−1.69	1.57	−1.34	0.00	0.000	4.43
Smim15	−1.69	4.63	−12.64	0.00	0.000	10.57
Map1a	−1.69	2.81	−1.01	0.00	0.000	6.61
Dnase1l1	−1.69	5.01	−14.40	0.00	0.000	12.18
Porcn	−1.69	1.80	−1.01	0.00	0.000	2.32
Abtb1	−1.69	4.68	−15.73	0.00	0.000	13.35
Nsmaf	−1.69	4.24	−14.24	0.00	0.000	12.12
Dhrs3	−1.68	1.47	−1.32	0.00	0.000	1.15
Axl	−1.68	8.27	−19.58	0.00	0.000	16.08
Rps6ka5	−1.68	2.45	−1.12	0.00	0.000	5.46
Ddt	−1.68	1.52	−1.65	0.00	0.000	3.37
Crtap	−1.67	5.46	−14.69	0.00	0.000	12.42
Arhgef37	−1.67	−1.16	−1.21	0.00	0.002	-0.63
Spag6	−1.67	2.40	−1.77	0.00	0.000	3.44
Mmp11	−1.67	6.27	−14.78	0.00	0.000	12.43
Slc9a5	−1.67	5.80	−1.39	0.00	0.000	3.87
Mindy2	−1.67	4.06	−1.93	0.00	0.000	7.59
Zfp692	−1.67	4.91	−1.74	0.00	0.000	2.98
Rps12-ps9	−1.66	1.15	−1.16	0.00	0.002	-0.87
Bbc3	−1.66	5.35	−10.54	0.00	0.000	8.16
Ecm1	−1.66	5.62	−14.72	0.00	0.000	12.42
Rab3a	−1.66	1.01	−1.94	0.00	0.000	2.28
Klf4	−1.66	5.92	−16.67	0.00	0.000	14.02
Gm46209	−1.66	0.28	−1.18	0.00	0.002	-0.71
Thbs3	−1.66	3.78	−1.29	0.00	0.000	5.54
Rasip1	−1.66	1.43	−1.32	0.00	0.002	-0.64
Rpl22l1	−1.66	4.61	−1.32	0.00	0.000	2.33
Bckdha	−1.66	5.77	−16.90	0.00	0.000	14.21
Peak1	−1.66	6.05	−12.20	0.00	0.000	9.95
Nfkbie	−1.65	2.18	−1.17	0.00	0.000	5.56
Tor3a	−1.65	5.03	−15.75	0.00	0.000	13.34
Kyat1	−1.65	4.05	−11.47	0.00	0.000	9.40
Gm49284	−1.65	1.24	−1.54	0.00	0.001	-0.20
Tm7sf2	−1.65	1.73	−1.48	0.00	0.000	4.61
Copz2	−1.64	4.13	−13.57	0.00	0.000	11.50
Nat14	−1.64	1.94	−1.88	0.00	0.000	3.68
AC160336.1	−1.64	0.29	−1.68	0.02	0.030	-3.47
Prune2	−1.64	5.84	−1.33	0.00	0.000	6.60
Gm4961	−1.63	1.09	−1.78	0.00	0.000	3.60
D430040D24Rik	−1.63	0.49	−1.19	0.00	0.000	0.99
Rpl17-ps3	−1.63	2.18	−1.92	0.00	0.000	2.10
Atp2c2	−1.63	1.56	−1.61	0.00	0.000	1.63
Per3	−1.63	4.23	−10.86	0.00	0.000	8.68
Sh3bp1	−1.63	4.40	−1.52	0.00	0.000	5.70
Col6a1	−1.63	8.46	−11.40	0.00	0.000	17.26
Dbp	−1.63	6.30	−11.76	0.00	0.000	9.45
Osbp2	−1.63	3.67	−10.92	0.00	0.000	8.84
Lgals3bp	−1.63	5.69	−15.14	0.00	0.000	12.77
Dand5	−1.63	1.45	−1.02	0.00	0.000	3.95

Lama5	−1.62	8.19	−11.80	0.00	0.000	9.43
Smim11	−1.62	3.70	−1.29	0.00	0.000	6.85
Abca8b	−1.62	2.50	−1.30	0.00	0.000	4.27
Calcoco1	−1.62	6.18	−19.27	0.00	0.000	15.91
Rgs9bp	−1.62	0.25	−1.64	0.00	0.001	0.13
H2-T22	−1.62	5.22	−15.36	0.00	0.000	13.00
Fam8a1	−1.62	5.77	−15.95	0.00	0.000	13.46
Pdcd4	−1.61	6.26	−17.66	0.00	0.000	14.76
Cst3	−1.61	6.82	−14.83	0.00	0.000	12.43
Plppr2	−1.61	4.87	−13.97	0.00	0.000	11.82
Sardh	−1.61	4.89	−14.40	0.00	0.000	12.19
Tph1	−1.61	0.80	−1.43	0.00	0.002	-0.34
Itpka	−1.61	1.28	−1.25	0.00	0.000	4.27
Olfml2b	−1.61	1.02	−1.98	0.00	0.000	2.33
E230013L22Rik	−1.60	−1.22	−1.82	0.00	0.004	-1.38
Abca7	−1.60	5.32	−1.07	0.00	0.000	6.33
Etfbkmt	−1.60	3.94	−10.61	0.00	0.000	8.45
Tmem198b	−1.60	4.60	−12.61	0.00	0.000	10.53
Zfp455	−1.60	0.48	−1.31	0.00	0.002	-0.51
Snx29	−1.60	1.01	−1.23	0.00	0.002	-0.75
Gm28229	−1.60	1.14	−1.37	0.00	0.002	-0.46
Tcea2	−1.60	2.55	−1.54	0.00	0.000	3.08
Ccdc62	−1.60	0.07	−1.36	0.00	0.002	-0.37
Siae	−1.60	4.08	−13.15	0.00	0.000	11.11
Pde4b	−1.59	2.74	−1.40	0.00	0.000	5.81
Pcdhgc5	−1.59	3.91	−1.19	0.00	0.000	3.81
Tnni3	−1.59	0.39	−1.00	0.00	0.003	-1.07
Defb25	−1.59	0.68	−1.88	0.00	0.004	-1.34
Tmem231	−1.59	3.70	−10.14	0.00	0.000	7.91
Grina	−1.58	8.49	−17.28	0.00	0.000	14.42
Tmem185a	−1.58	6.06	−17.53	0.00	0.000	14.68
Gm26947	−1.58	3.09	−1.37	0.00	0.000	1.00
Pet100	−1.58	2.82	−1.61	0.00	0.000	4.66
Gm12940	−1.58	1.73	−1.24	0.00	0.002	-0.83
Camsap3	−1.58	−1.11	−1.73	0.00	0.005	-1.58
Nicn1	−1.58	3.82	−13.20	0.00	0.000	11.17
Pisd-ps1	−1.58	1.98	−1.46	0.00	0.000	3.02
St3gal2	−1.58	6.24	−16.85	0.00	0.000	14.14
Megf9	−1.58	2.91	−1.36	0.00	0.000	4.28
Snhg20	−1.57	2.69	−1.16	0.00	0.002	-1.14
Fbxo31	−1.57	6.54	−14.21	0.00	0.000	11.89
Gm4342	−1.57	−1.06	−1.59	0.00	0.001	0.04
Abcb10	−1.57	5.76	−16.21	0.00	0.000	13.66
Ust	−1.57	4.76	−13.52	0.00	0.000	11.40
Ache	−1.57	0.78	−1.62	0.00	0.001	-0.01
Lgals8	−1.56	5.89	−15.44	0.00	0.000	13.02
Irgm1	−1.56	3.88	−12.69	0.00	0.000	10.67
Akr1b8	−1.56	6.01	−13.43	0.00	0.000	11.20
A530017D24Rik	−1.56	3.03	−1.79	0.00	0.000	6.29
Tmem50a	−1.56	6.19	−16.55	0.00	0.000	13.92
Cox17	−1.56	0.23	−1.38	0.03	0.050	-3.96

Trim7	−1.56	2.82	−1.66	0.00	0.000	4.71
Aox1	−1.56	3.36	−1.18	0.00	0.000	2.28
4931406C07Rik	−1.56	4.86	−14.65	0.00	0.000	12.41
Adamtsl3	−1.55	3.45	−1.94	0.00	0.000	5.08
Abhd4	−1.55	5.93	−14.91	0.00	0.000	12.56
Rpl17-ps8	−1.55	0.47	−1.53	0.00	0.001	-0.11
Fstl1	−1.55	7.38	−18.47	0.00	0.000	15.31
Idh1	−1.55	5.52	−14.68	0.00	0.000	12.39
Gm13375	−1.55	1.81	−1.38	0.00	0.000	4.46
9330188P03Rik	−1.54	3.97	−1.40	0.00	0.000	4.16
Mrgpre	−1.54	2.77	−1.19	0.00	0.000	5.51
Abhd1	−1.54	1.39	−1.54	0.00	0.001	-0.23
Rap1gap	−1.54	5.10	−13.76	0.00	0.000	11.59
H2-D1	−1.54	10.03	−18.29	0.00	0.000	15.17
Dpy19l1	−1.54	5.12	−12.17	0.00	0.000	10.02
Klhl36	−1.54	2.74	−1.87	0.00	0.000	5.05
mt-Nd1	−1.54	9.90	−1.54	0.00	0.007	-3.16
Lrrc15	−1.54	3.66	−13.70	0.00	0.000	11.61
Gm12454	−1.54	0.56	−1.75	0.00	0.001	0.31
Pex6	−1.54	6.11	−14.70	0.00	0.000	12.36
A430103D13Rik	−1.53	1.45	−1.26	0.00	0.002	-0.77
CT010445.1	−1.53	1.49	−1.48	0.00	0.008	-2.21
Pcdhgb2	−1.53	2.44	−1.06	0.00	0.000	2.25
Fam76a	−1.53	5.09	−14.23	0.00	0.000	12.03
Gpsm3	−1.53	1.50	−1.00	0.00	0.000	2.29
Zfp874b	−1.52	3.52	−11.18	0.00	0.000	9.13
Ccdc181	−1.52	5.68	−11.61	0.00	0.000	9.35
Adgre5	−1.52	5.25	−13.33	0.00	0.000	11.15
Qk	−1.52	6.58	−11.08	0.00	0.000	8.68
Relb	−1.52	4.90	−1.50	0.00	0.000	6.94
Ago3	−1.52	4.30	−1.78	0.00	0.000	3.12
Pnpla7	−1.52	4.47	−13.39	0.00	0.000	11.30
Irf8	−1.52	5.08	−13.83	0.00	0.000	11.67
Nradd	−1.52	1.81	−1.21	0.00	0.000	4.18
Prob1	−1.52	3.18	−1.13	0.00	0.000	5.36
Fbln2	−1.51	8.40	−18.98	0.00	0.000	15.66
Akt3	−1.51	6.63	−12.72	0.00	0.000	10.45
Sdccag8	−1.51	3.71	−10.14	0.00	0.000	7.89
Apc2	−1.51	3.60	−11.91	0.00	0.000	9.86
BC048644	−1.51	1.17	−1.11	0.00	0.000	2.55
Gm49405	−1.51	0.51	−1.92	0.01	0.020	-3.10
Pqlc3	−1.51	5.15	−13.36	0.00	0.000	11.20
Zc3h6	−1.51	3.62	−1.12	0.00	0.001	0.41
Pdgfra	−1.50	6.93	−13.38	0.00	0.000	11.09
Tmc4	−1.50	2.77	−1.48	0.00	0.000	2.92
Khk	−1.50	4.60	−12.09	0.00	0.000	9.96
Raet1e	−1.50	4.67	−12.74	0.00	0.000	10.64
Cd82	−1.50	5.68	−12.57	0.00	0.000	10.37
mt-Nd5	−1.50	9.45	−1.04	0.01	0.016	-4.15
Elk3	−1.49	6.50	−16.72	0.00	0.000	14.03
Tmco6	−1.49	3.04	−1.32	0.00	0.000	6.99

Btc	−1.49	2.23	−1.35	0.00	0.000	2.81
1600020E01Rik	−1.49	2.09	−1.95	0.00	0.001	0.39
Tnfaip8	−1.49	5.21	−14.96	0.00	0.000	12.65
Anpep	−1.49	7.78	−16.65	0.00	0.000	13.93
Ccdc82	−1.49	4.15	−11.25	0.00	0.000	9.13
Sez6l2	−1.49	4.84	−14.65	0.00	0.000	12.41
Tnfrsf9	−1.49	4.65	−13.46	0.00	0.000	11.34
Speg	−1.49	4.31	−11.21	0.00	0.000	9.06
Itm2c	−1.49	6.84	−16.78	0.00	0.000	14.06
Rgs11	−1.49	3.18	−1.32	0.00	0.002	-1.04
Tm6sf1	−1.49	2.29	−1.36	0.00	0.000	4.37
Acot13	−1.48	5.40	−11.70	0.00	0.000	9.47
Spata2l	−1.48	1.36	−1.61	0.00	0.000	1.65
Nlgn2	−1.48	4.78	−13.42	0.00	0.000	11.28
Ak5	−1.48	0.02	−1.98	0.00	0.003	-1.03
Gnb5	−1.48	4.79	−13.39	0.00	0.000	11.25
Gdi1	−1.48	7.60	−12.72	0.00	0.000	10.41
Lrrn4cl	−1.48	2.40	−1.42	0.00	0.000	4.41
Npc2	−1.48	8.05	−16.01	0.00	0.000	13.41
Prkra	−1.48	4.51	−11.24	0.00	0.000	9.07
Ttc41	−1.48	0.11	−1.00	0.00	0.003	-1.03
4833407H14Rik	−1.48	1.54	−1.99	0.00	0.000	2.30
Frs3	−1.48	2.33	−1.00	0.00	0.000	3.81
Zswim4	−1.47	6.04	−13.94	0.00	0.000	11.68
Adamts20	−1.47	2.77	−1.32	0.00	0.000	2.71
Heg1	−1.47	5.01	−13.61	0.00	0.000	11.45
Tdpx-ps1	−1.47	0.22	−1.95	0.00	0.003	-1.13
Sptssa	−1.47	4.35	−1.79	0.00	0.000	7.40
Espnl	−1.47	0.07	−1.81	0.00	0.001	0.42
Arhgap45	−1.47	6.17	−14.81	0.00	0.000	12.45
Caprin2	−1.46	5.30	−10.10	0.00	0.000	7.64
Atpif1	−1.46	7.18	−13.89	0.00	0.000	11.57
Igip	−1.46	4.67	−10.01	0.00	0.000	7.61
Osbpl3	−1.46	5.66	−15.64	0.00	0.000	13.20
Txndc12	−1.46	6.45	−14.35	0.00	0.000	12.02
Mgst1	−1.46	5.58	−14.92	0.00	0.000	12.59
Plp1	−1.46	2.82	−1.92	0.00	0.001	0.22
Akr1b10	−1.46	4.84	−13.83	0.00	0.000	11.68
Parp10	−1.46	4.47	−10.83	0.00	0.000	8.59
Glt8d1	−1.45	4.80	−1.63	0.00	0.000	7.11
Gm14403	−1.45	0.74	−1.24	0.00	0.002	-0.69
Ccpg1os	−1.45	1.06	−1.18	0.00	0.000	0.96
Spata1	−1.45	1.41	−1.41	0.00	0.000	1.32
Cacna1c	−1.45	3.79	−1.33	0.00	0.000	5.52
4732471J01Rik	−1.45	−1.10	−1.93	0.00	0.004	-1.12
Tmem53	−1.45	1.75	−1.97	0.00	0.000	2.20
Zscan2	−1.45	2.10	−1.26	0.00	0.000	0.96
5830444B04Rik	−1.45	1.93	−1.19	0.00	0.000	4.13
Tln1	−1.45	7.97	−16.06	0.00	0.000	13.45
Slc38a9	−1.45	3.63	−1.70	0.00	0.000	3.12
Lgmn	−1.45	7.91	−16.35	0.00	0.000	13.69

Prpf40b	−1.45	4.51	−1.58	0.00	0.000	7.11
Gm38560	−1.45	0.23	−1.89	0.00	0.004	-1.23
Rpl13a	−1.45	5.27	−10.32	0.00	0.000	7.92
Naip6	−1.45	0.25	−1.94	0.00	0.003	-1.15
Oasl1	−1.45	1.21	−1.15	0.00	0.001	0.85
Zfand6	−1.45	5.37	−11.11	0.00	0.000	8.83
Adck5	−1.44	4.52	−10.45	0.00	0.000	8.15
Lrtm2	−1.44	0.09	−1.52	0.00	0.007	-1.96
Ppp1r18	−1.44	6.90	−16.25	0.00	0.000	13.64
Mrap	−1.44	3.76	−11.24	0.00	0.000	9.15
Itpr2	−1.44	5.77	−14.37	0.00	0.000	12.09
Acp5	−1.44	3.58	−10.02	0.00	0.000	7.75
Nid1	−1.44	7.52	−11.52	0.00	0.000	9.14
Ino80dos	−1.44	0.12	−1.80	0.00	0.004	-1.45
Bbs9	−1.44	3.17	−1.51	0.00	0.000	5.88
Gm8692	−1.44	0.40	−1.56	0.00	0.001	-0.04
Cd37	−1.44	4.25	−11.36	0.00	0.000	9.23
Sirt5	−1.44	4.04	−12.05	0.00	0.000	9.99
Itm2b	−1.44	8.18	−17.35	0.00	0.000	14.48
Rnf13	−1.44	6.05	−12.15	0.00	0.000	9.90
1700120C14Rik	−1.44	2.18	−1.83	0.00	0.004	-1.69
Sdcbp2	−1.44	0.09	−1.01	0.00	0.003	-1.03
Asah2	−1.44	4.30	−1.50	0.00	0.000	7.02
2310069B03Rik	−1.44	0.71	−1.06	0.00	0.001	0.81
Arhgef25	−1.43	5.95	−10.54	0.00	0.000	8.09
Gdap1l1	−1.43	2.76	−1.22	0.00	0.000	2.52
Syt2	−1.43	1.85	−1.39	0.00	0.000	2.91
Selenom	−1.43	5.61	−12.23	0.00	0.000	10.02
Ap3m2	−1.43	3.43	−1.19	0.00	0.000	6.75
Il17rc	−1.43	5.51	−13.19	0.00	0.000	11.00
Bdh2	−1.43	1.70	−1.26	0.00	0.002	-0.83
Elmo3	−1.43	0.31	−1.80	0.01	0.024	-3.27
Hspb2	−1.42	1.82	−1.88	0.00	0.000	3.70
Slc25a37	−1.42	6.98	−1.96	0.00	0.000	7.32
Nfia	−1.42	5.81	−14.87	0.00	0.000	12.53
Ikbip	−1.42	5.86	−11.70	0.00	0.000	9.42
Wipi1	−1.42	5.33	−12.29	0.00	0.000	10.11
Guca1a	−1.42	3.60	−1.29	0.00	0.000	2.43
Rps19-ps3	−1.41	1.46	−1.68	0.00	0.000	1.79
Dbndd2	−1.41	1.07	−1.31	0.00	0.002	-0.59
Clmp	−1.41	4.01	−1.64	0.00	0.000	7.23
Insl6	−1.41	2.97	−1.64	0.00	0.000	3.13
Gm17494	−1.41	0.96	−1.66	0.00	0.006	-1.82
Dnase2a	−1.41	6.15	−16.44	0.00	0.000	13.82
Fermt2	−1.40	6.34	−12.48	0.00	0.000	10.21
Akap6	−1.40	3.49	−1.03	0.00	0.000	2.03
Gm7363	−1.40	0.35	−1.05	0.00	0.003	-0.97
Abhd14a	−1.40	4.93	−13.32	0.00	0.000	11.18
9530018F02Rik	−1.40	0.00	−1.19	0.00	0.002	-0.68
Slc25a29	−1.40	3.05	−1.26	0.00	0.000	5.55
Naa80	−1.40	4.15	−1.85	0.00	0.000	4.79

Rpl17	−1.40	9.71	−1.29	0.00	0.000	3.55
Asap1	−1.40	6.82	−14.20	0.00	0.000	11.87
Cd40	−1.40	4.11	−1.20	0.00	0.000	6.70
Sept2	−1.40	0.53	−1.03	0.01	0.016	-2.90
Ttc23	−1.40	3.97	−11.56	0.00	0.000	9.50
Amt	−1.40	0.27	−1.00	0.00	0.003	-1.05
Acsf2	−1.40	5.65	−13.17	0.00	0.000	10.96
Map1lc3b	−1.39	6.58	−17.01	0.00	0.000	14.24
Gm40723	−1.39	0.12	−1.51	0.00	0.007	-1.91
Npr2	−1.39	5.09	−10.87	0.00	0.000	8.58
C2cd2l	−1.39	5.09	−14.09	0.00	0.000	11.90
Trp53inp1	−1.39	3.67	−1.92	0.00	0.000	4.97
E130311K13Rik	−1.39	3.86	−10.57	0.00	0.000	8.40
Cfp	−1.39	0.80	−1.40	0.00	0.009	-2.25
Zfp398	−1.39	4.85	−1.82	0.00	0.000	4.61
Ifit2	−1.39	3.70	−1.45	0.00	0.000	5.73
Trpt1	−1.39	2.31	−1.23	0.00	0.000	4.15
Akr1e1	−1.39	5.27	−14.27	0.00	0.000	12.04
Sp140	−1.39	2.54	−1.97	0.00	0.000	5.21
Dguok	−1.38	3.87	−1.19	0.00	0.000	5.34
Nbr1	−1.38	5.31	−12.35	0.00	0.000	10.18
F2r	−1.38	6.61	−12.73	0.00	0.000	10.46
Cyba	−1.38	6.10	−10.16	0.00	0.000	7.61
Slc35d2	−1.38	2.28	−1.26	0.00	0.000	4.19
Zrsr1	−1.38	4.73	−12.62	0.00	0.000	10.50
Samd14	−1.38	1.84	−1.11	0.00	0.000	2.47
Gm5075	−1.38	1.74	−1.66	0.00	0.000	1.72
Gm8624	−1.37	0.35	−1.07	0.00	0.003	-0.93
Gm11613	−1.37	0.41	−1.93	0.00	0.001	0.57
Dram2	−1.37	4.53	−10.82	0.00	0.000	8.58
Pak1	−1.37	6.45	−16.26	0.00	0.000	13.65
Rundc3a	−1.37	6.39	−14.44	0.00	0.000	12.11
Smpd2	−1.37	4.43	−10.71	0.00	0.000	8.48
Zfpm1	−1.37	6.02	−11.16	0.00	0.000	8.82
Pim1	−1.37	5.48	−10.67	0.00	0.000	8.30
Prickle3	−1.37	3.52	−1.11	0.00	0.000	5.26
Col6a3	−1.36	3.22	−1.62	0.00	0.000	4.61
Col5a3	−1.36	7.42	−14.83	0.00	0.000	12.41
Gm19705	−1.36	0.35	−1.74	0.00	0.005	-1.58
Rpl17-ps5	−1.36	4.52	−1.80	0.00	0.000	3.10
Arhgap22	−1.36	6.44	−14.01	0.00	0.000	11.72
Trpm4	−1.36	3.16	−1.93	0.00	0.000	3.52
Lrrc45	−1.36	4.95	−10.08	0.00	0.000	7.65
Ube2h	−1.36	7.03	−17.44	0.00	0.000	14.56
Csrp2	−1.36	3.97	−1.13	0.00	0.000	5.23
Fbxl20	−1.36	4.96	−13.42	0.00	0.000	11.27
Polk	−1.36	4.58	−1.40	0.00	0.000	6.86
Foxs1	−1.36	5.66	−12.84	0.00	0.000	10.62
Tpra1	−1.36	5.49	−12.40	0.00	0.000	10.21
Ccng2	−1.36	3.17	−1.06	0.00	0.001	0.34
Loxl2	−1.36	4.84	−12.04	0.00	0.000	9.89

Map3k13	−1.35	1.60	−1.99	0.00	0.001	0.57
Hpse	−1.35	0.18	−1.15	0.01	0.013	-2.63
Smpdl3b	−1.35	5.03	−14.34	0.00	0.000	12.10
Ccnl2	−1.35	6.13	−1.26	0.00	0.000	5.11
Rexo5	−1.35	3.33	−1.88	0.00	0.000	1.84
Tspan17	−1.35	5.00	−10.75	0.00	0.000	8.44
Hivep3	−1.35	4.29	−1.31	0.00	0.000	6.79
E430024I08Rik	−1.35	1.76	−1.92	0.00	0.004	-1.48
Tifa	−1.35	2.73	−1.88	0.00	0.000	3.58
Slc25a30	−1.35	6.13	−12.41	0.00	0.000	10.17
Tatdn3	−1.34	2.53	−1.71	0.00	0.000	4.86
Lima1	−1.34	6.85	−15.92	0.00	0.000	13.37
Socs3	−1.34	4.67	−12.71	0.00	0.000	10.60
Rhox2h	−1.34	5.03	−1.67	0.00	0.000	7.14
Creb3l1	−1.34	1.00	−1.26	0.00	0.000	1.12
Mrpl33	−1.34	4.57	−1.96	0.00	0.000	3.37
C730034F03Rik	−1.34	−1.19	−1.92	0.01	0.020	-2.97
Sil1	−1.34	5.08	−11.01	0.00	0.000	8.75
Cryz	−1.34	5.02	−12.47	0.00	0.000	10.32
Trerf1	−1.33	2.13	−1.25	0.00	0.000	4.19
Sec16b	−1.33	4.90	−10.14	0.00	0.000	7.74
Cyb5d2	−1.33	2.52	−1.36	0.00	0.000	2.73
Stx11	−1.33	4.35	−1.70	0.00	0.000	7.27
Abca2	−1.33	7.01	−13.09	0.00	0.000	10.79
Gm16288	−1.33	1.15	−1.94	0.01	0.019	-3.20
Lats2	−1.33	4.04	−10.69	0.00	0.000	8.52
Rps12	−1.33	10.57	−1.54	0.00	0.000	0.63
Shld2	−1.33	5.46	−11.50	0.00	0.000	9.24
Adhfe1	−1.33	3.60	−1.28	0.00	0.000	6.81
Gm20544	−1.32	0.84	−1.85	0.00	0.004	-1.50
Gm37254	−1.32	1.23	−1.32	0.00	0.002	-0.58
Myo6	−1.32	5.10	−1.50	0.00	0.000	6.90
Col16a1	−1.32	7.71	−14.74	0.00	0.000	12.33
Hspb11	−1.32	4.44	−1.00	0.00	0.000	6.35
Gm49774	−1.32	2.65	−1.64	0.00	0.000	3.24
AC122818.3	−1.32	2.14	−1.32	0.00	0.000	1.07
Mfsd4b4	−1.32	3.15	−1.79	0.00	0.000	4.87
Dst	−1.32	7.56	−1.94	0.00	0.000	7.27
Gzmm	−1.32	−1.07	−1.13	0.01	0.014	-2.63
Slc25a20	−1.32	2.74	−1.70	0.00	0.000	3.24
Gm14494	−1.32	3.59	−1.27	0.00	0.000	2.41
Trp53inp2	−1.32	4.15	−1.70	0.00	0.000	7.29
Rab24	−1.31	6.04	−13.44	0.00	0.000	11.19
St3gal6	−1.31	4.99	−11.66	0.00	0.000	9.46
Trim2	−1.31	3.55	−1.26	0.00	0.000	5.46
C430049B03Rik	−1.31	−1.06	−1.83	0.00	0.004	-1.35
Nr1d1	−1.31	6.79	−1.99	0.00	0.000	7.37
Pdlim5	−1.31	5.43	−1.10	0.00	0.000	6.35
Dnajb4	−1.31	5.95	−11.32	0.00	0.000	8.99
Zmat5	−1.31	3.71	−1.68	0.00	0.000	4.58
Atg9b	−1.31	3.77	−1.31	0.00	0.000	0.65

Ptpn14	−1.31	5.57	−1.88	0.00	0.000	7.34
Ucn2	−1.31	0.20	−1.42	0.03	0.047	-3.96
Vbp1	−1.31	7.36	−11.86	0.00	0.000	9.51
Amotl2	−1.31	5.67	−13.40	0.00	0.000	11.18
Nudt16	−1.30	1.58	−1.60	0.00	0.000	1.62
Zbtb11os1	−1.30	0.70	−1.22	0.00	0.002	-0.77
Tgfbi	−1.30	5.03	−13.75	0.00	0.000	11.55
Lrig3	−1.30	5.16	−12.98	0.00	0.000	10.83
Xdh	−1.30	7.38	−15.85	0.00	0.000	13.29
Bcas3	−1.30	4.34	−1.28	0.00	0.000	5.36
S1pr2	−1.30	4.68	−11.35	0.00	0.000	9.16
4930481A15Rik	−1.30	−1.12	−1.50	0.00	0.007	-1.99
Ccdc122	−1.30	1.81	−1.47	0.00	0.001	-0.47
Acsf3	−1.30	2.54	−1.36	0.00	0.000	2.79
Hid1	−1.30	4.95	−10.87	0.00	0.000	8.59
Srgap3	−1.30	6.63	−12.34	0.00	0.000	10.04
Gm4707	−1.29	−1.11	−1.47	0.03	0.043	-3.79
Chst13	−1.29	1.43	−1.05	0.00	0.003	-1.13
Sh2d6	−1.29	2.09	−1.32	0.00	0.002	-0.66
Plod1	−1.29	6.48	−15.48	0.00	0.000	13.01
Commd3	−1.29	6.12	−12.39	0.00	0.000	10.14
Kank3	−1.29	0.46	−1.90	0.00	0.001	0.48
Mppe1	−1.29	3.42	−1.97	0.00	0.000	6.44
Zfp949	−1.29	2.70	−1.95	0.00	0.000	3.67
Nqo2	−1.29	5.10	−13.44	0.00	0.000	11.29
Gm10054	−1.29	0.81	−1.95	0.00	0.003	-1.21
Serinc1	−1.29	7.31	−11.42	0.00	0.000	9.03
Pam	−1.28	6.80	−14.81	0.00	0.000	12.42
Zfp658	−1.28	0.94	−1.26	0.00	0.002	-0.69
Spa17	−1.28	0.13	−1.24	0.01	0.012	-2.46
Ccdc171	−1.28	1.63	−1.99	0.00	0.001	0.52
Rps12-ps3	−1.28	2.47	−1.15	0.00	0.002	-1.15
Rhox2g	−1.28	4.52	−11.82	0.00	0.000	9.70
Lancl1	−1.28	6.11	−14.46	0.00	0.000	12.15
Prr13	−1.28	6.70	−11.18	0.00	0.000	8.78
4921524J17Rik	−1.28	4.73	−1.63	0.00	0.000	5.80
Ggcx	−1.28	3.97	−1.83	0.00	0.000	6.21
Rpl17-ps10	−1.28	5.38	−1.63	0.00	0.000	2.69
Nlrc5	−1.28	4.76	−10.14	0.00	0.000	7.76
Dirc2	−1.28	4.60	−10.78	0.00	0.000	8.52
Cacng7	−1.28	4.37	−10.57	0.00	0.000	8.30
Sertad3	−1.28	3.66	−10.07	0.00	0.000	7.82
Pls3	−1.28	6.75	−13.31	0.00	0.000	11.03
Slc39a10	−1.27	3.98	−1.58	0.00	0.000	4.47
6030445D17Rik	−1.27	−1.01	−1.20	0.01	0.012	-2.51
Gm45495	−1.27	0.12	−1.41	0.00	0.002	-0.33
Eng	−1.27	7.33	−11.34	0.00	0.000	8.92
Cast	−1.27	6.69	−12.06	0.00	0.000	9.76
Hibadh	−1.27	6.84	−15.89	0.00	0.000	13.34
Trmu	−1.27	3.15	−1.55	0.00	0.000	2.96
Fblim1	−1.27	5.51	−13.59	0.00	0.000	11.37

Mvd	−1.27	4.38	−1.81	0.00	0.000	7.37
Klra4	−1.27	2.04	−1.35	0.00	0.000	1.12
Hes7	−1.27	4.97	−1.78	0.00	0.000	5.96
Lurap1	−1.27	2.92	−1.19	0.00	0.000	3.98
A530020G20Rik	−1.27	2.94	−1.70	0.00	0.005	-2.17
Slc46a1	−1.27	1.73	−1.50	0.00	0.000	1.42
Ago4	−1.27	3.64	−1.67	0.00	0.000	4.59
Rsrp1	−1.27	5.10	−1.44	0.00	0.002	-1.16
Svbp	−1.27	2.83	−1.83	0.00	0.001	0.02
Cyp4v3	−1.27	1.55	−1.14	0.00	0.001	0.81
Hspg2	−1.26	9.40	−12.39	0.00	0.000	10.05
Iqsec2	−1.26	2.21	−1.76	0.00	0.000	1.79
Nfat5	−1.26	7.06	−1.39	0.00	0.000	2.13
B330016D10Rik	−1.26	0.39	−1.37	0.00	0.009	-2.26
Ropn1l	−1.26	0.27	−1.78	0.00	0.005	-1.51
AW549877	−1.26	5.66	−1.49	0.00	0.000	5.48
Dpp7	−1.26	4.89	−1.73	0.00	0.000	4.48
Gm30074	−1.26	1.46	−1.67	0.00	0.006	-1.97
Gm28439	−1.26	5.05	−1.69	0.02	0.030	-4.50
Adamts4	−1.26	4.11	−1.41	0.00	0.000	5.58
Usp18	−1.26	2.25	−1.06	0.00	0.000	6.59
Gstm2	−1.26	5.65	−12.80	0.00	0.000	10.59
Mettl15	−1.26	1.90	−1.71	0.00	0.001	0.00
Ifi35	−1.26	4.38	−1.71	0.00	0.000	5.93
Gm12816	−1.26	1.11	−1.74	0.00	0.001	0.19
Snx10	−1.26	4.92	−1.48	0.00	0.000	5.58
Cep41	−1.25	4.04	−1.45	0.00	0.000	6.98
Tmem145	−1.25	0.82	−1.53	0.00	0.007	-2.02
Tm2d1	−1.25	3.73	−1.12	0.00	0.000	3.78
Gm13848	−1.25	1.97	−1.43	0.00	0.000	2.94
B2m	−1.25	9.62	−13.53	0.00	0.000	11.20
Pitpnm1	−1.25	5.91	−1.84	0.00	0.000	7.24
Fuz	−1.25	3.79	−1.05	0.00	0.000	6.50
Zfp580	−1.25	3.17	−1.42	0.00	0.000	4.32
Thap3	−1.25	5.07	−11.35	0.00	0.000	9.11
Mylpf	−1.25	0.46	−1.84	0.00	0.004	-1.41
Cmc4	−1.25	1.66	−1.40	0.00	0.000	1.26
Insig1	−1.25	5.81	−10.74	0.00	0.000	8.33
Pltp	−1.25	7.11	−12.46	0.00	0.000	10.14
Cfap58	−1.25	0.55	−1.24	0.01	0.011	-2.49
Stap2	−1.24	1.50	−1.34	0.00	0.000	1.16
Cotl1	−1.24	5.25	−10.51	0.00	0.000	8.13
Cfap57	−1.24	0.46	−1.76	0.00	0.001	0.25
E230016M11Rik	−1.24	1.75	−1.40	0.00	0.002	-0.56
Vps37d	−1.24	1.27	−1.97	0.00	0.001	0.51
Mknk1	−1.24	5.93	−13.74	0.00	0.000	11.50
Slc26a11	−1.24	3.95	−1.92	0.00	0.000	4.93
Hap1	−1.24	0.49	−1.16	0.00	0.002	-0.83
Akap13	−1.24	5.16	−1.71	0.00	0.000	5.82
Asap3	−1.24	2.63	−1.90	0.00	0.000	5.08
Sat1	−1.23	5.40	−1.91	0.00	0.000	4.67

Rad52	−1.23	4.83	−1.77	0.00	0.000	7.28
Pfdn5	−1.23	7.25	−10.27	0.00	0.000	7.69
Fam214b	−1.23	4.34	−10.69	0.00	0.000	8.48
Col6a2	−1.23	7.15	−15.09	0.00	0.000	12.64
Syne2	−1.23	7.68	−1.52	0.00	0.000	5.38
Slc37a2	−1.23	2.09	−1.78	0.00	0.001	0.11
9130008F23Rik	−1.23	1.23	−1.41	0.00	0.002	-0.42
Tmem106c	−1.23	4.73	−10.30	0.00	0.000	7.93
Rpl15-ps2	−1.23	0.49	−1.02	0.00	0.003	-1.04
Gm14286	−1.23	0.03	−1.42	0.03	0.047	-3.89
Sorbs3	−1.23	5.91	−12.74	0.00	0.000	10.50
Inpp5k	−1.23	3.84	−1.85	0.00	0.000	4.80
Cpne7	−1.23	4.62	−1.39	0.00	0.000	4.02
Psmb8	−1.23	4.18	−1.38	0.00	0.000	6.87
Mmaa	−1.23	3.64	−1.78	0.00	0.000	4.77
Plekha3	−1.23	4.79	−10.10	0.00	0.000	7.71
Fam131a	−1.23	2.91	−1.81	0.00	0.000	3.40
Six5	−1.23	3.82	−1.46	0.00	0.000	4.23
Ss18l2	−1.23	3.53	−1.49	0.00	0.000	5.81
Dennd5b	−1.22	5.74	−1.63	0.00	0.000	5.66
Gramd2	−1.22	0.58	−1.81	0.00	0.004	-1.47
Zfp579	−1.22	3.57	−1.11	0.00	0.000	2.12
Dab2	−1.22	5.65	−1.45	0.00	0.000	6.77
Aamdc	−1.22	2.97	−1.42	0.00	0.000	2.72
Mtln	−1.22	2.52	−1.42	0.00	0.000	1.18
Sirt4	−1.22	2.82	−1.02	0.00	0.000	3.75
Arsg	−1.22	1.97	−1.46	0.00	0.000	2.97
Lipt1	−1.22	3.08	−1.10	0.00	0.000	3.82
Zbtb12	−1.22	4.96	−12.13	0.00	0.000	9.95
Rusc2	−1.22	5.94	−13.20	0.00	0.000	10.99
Gm5644	−1.22	0.38	−1.56	0.00	0.007	-1.91
Tecr	−1.22	6.77	−12.11	0.00	0.000	9.80
Pecr	−1.22	4.53	−11.12	0.00	0.000	8.93
Cmbl	−1.22	1.25	−1.28	0.00	0.000	1.02
Timm21	−1.22	3.88	−1.76	0.00	0.000	4.70
Gm13657	−1.22	0.10	−1.62	0.00	0.006	-1.80
Bphl	−1.22	4.41	−11.17	0.00	0.000	8.99
Tnfsf13	−1.22	0.46	−1.31	0.01	0.010	-2.49
Rpl36-ps3	−1.22	0.15	−1.21	0.01	0.012	-2.53
Gm42688	−1.21	0.00	−1.87	0.01	0.022	-3.12
Aldh3b1	−1.21	4.97	−10.81	0.00	0.000	8.50
Nrg2	−1.21	3.83	−1.74	0.00	0.000	6.10
Map1lc3a	−1.21	4.99	−10.25	0.00	0.000	7.84
Tnfaip2	−1.21	6.93	−15.33	0.00	0.000	12.86
Gm41442	−1.21	1.46	−1.25	0.00	0.000	1.03
Irx5	−1.21	0.60	−1.43	0.00	0.008	-2.29
Man2b2	−1.21	5.45	−12.59	0.00	0.000	10.39
Dync2h1	−1.21	3.64	−1.12	0.00	0.000	3.77
Sdc2	−1.21	6.13	−13.96	0.00	0.000	11.68
Gm9828	−1.21	0.50	−1.69	0.00	0.005	-1.70
Vamp4	−1.21	5.13	−12.29	0.00	0.000	10.12

Ackr3	−1.21	3.17	−1.14	0.00	0.000	3.89
Kcnq1ot1	−1.21	3.55	−1.48	0.03	0.043	-4.66
Spsb2	−1.21	4.28	−1.12	0.00	0.000	5.13
Bcl2l12	−1.20	3.81	−1.92	0.00	0.000	3.40
Zfp40	−1.20	2.38	−1.09	0.00	0.000	2.33
Pbxip1	−1.20	4.92	−11.91	0.00	0.000	9.74
Plin2	−1.20	6.75	−12.49	0.00	0.000	10.20
Grk4	−1.20	2.43	−1.69	0.00	0.000	1.65
Zmym6	−1.20	4.81	−1.70	0.00	0.000	1.18
Zfp874a	−1.20	3.20	−1.86	0.00	0.000	3.44
Gm6055	−1.20	2.25	−1.01	0.00	0.000	2.22
Gm5963	−1.20	2.70	−1.47	0.00	0.001	-0.65
Rps12-ps4	−1.20	4.23	−1.80	0.00	0.001	-0.33
Rbms2	−1.20	6.36	−14.17	0.00	0.000	11.86
Rab3il1	−1.20	2.23	−1.21	0.00	0.000	2.48
Ralgds	−1.20	5.51	−12.85	0.00	0.000	10.65
5730455P16Rik	−1.20	3.70	−1.12	0.00	0.000	5.25
Jak3	−1.20	5.05	−11.92	0.00	0.000	9.72
Bcl2	−1.20	3.82	−1.15	0.00	0.000	6.66
Rb1	−1.20	4.01	−1.79	0.00	0.000	4.70
Zfp112	−1.20	0.41	−1.92	0.00	0.004	-1.25
Evi2a	−1.20	1.33	−1.47	0.00	0.001	-0.30
Gata2	1.20	1.99	4.56	0.00	0.001	-0.34
Slc25a21	1.20	0.37	3.82	0.00	0.004	-1.47
D930048N14Rik	1.20	1.16	4.38	0.00	0.002	-0.50
Slc35e1	1.20	5.80	12.46	0.00	0.000	10.22
Gm8666	1.20	1.46	4.79	0.00	0.001	0.22
Gstm7	1.20	1.59	5.32	0.00	0.000	1.06
Spag5	1.20	5.94	11.96	0.00	0.000	9.68
Ccnd3	1.20	6.74	9.78	0.00	0.000	7.09
Opn3	1.20	1.57	4.82	0.00	0.001	0.22
Ccdc91	1.20	5.99	11.59	0.00	0.000	9.28
Wdr4	1.20	5.82	11.73	0.00	0.000	9.44
Birc5	1.20	6.45	12.23	0.00	0.000	9.93
Srsf1	1.20	7.82	12.49	0.00	0.000	10.15
Gm6501	1.20	1.97	5.16	0.00	0.000	0.78
Eif2b1	1.21	5.73	9.95	0.00	0.000	7.37
Fancb	1.21	2.66	6.00	0.00	0.000	2.10
Sf3b3	1.21	7.86	13.37	0.00	0.000	11.04
AC090479.2	1.21	0.02	2.51	0.03	0.040	-3.76
Tpm3-rs7	1.21	4.25	9.40	0.00	0.000	6.87
Cse1l	1.21	6.58	12.36	0.00	0.000	10.07
Ccnf	1.21	6.61	10.99	0.00	0.000	8.55
Itga3	1.21	6.51	15.00	0.00	0.000	12.58
Lrrc73	1.22	1.96	4.41	0.00	0.002	-0.65
Gm7233	1.22	−1.20	2.65	0.02	0.031	-3.54
Mcm10	1.22	5.49	12.54	0.00	0.000	10.32
Aunip	1.22	4.34	8.80	0.00	0.000	6.05
Pex26	1.22	4.07	9.46	0.00	0.000	6.92
Sgsm1	1.22	5.08	9.93	0.00	0.000	7.42
Enoph1	1.22	5.54	13.79	0.00	0.000	11.56

Notch2	1.22	7.62	10.29	0.00	0.000	7.68
Gm7292	1.22	0.53	3.71	0.00	0.005	-1.70
Spns3	1.22	−1.08	3.58	0.00	0.006	-1.85
6030458C11Rik	1.22	5.54	13.68	0.00	0.000	11.44
Artn	1.22	3.53	8.94	0.00	0.000	6.38
AC131675.2	1.23	1.93	6.04	0.00	0.000	2.31
Spc24	1.23	4.93	11.92	0.00	0.000	9.73
Slc25a43	1.23	1.35	5.72	0.00	0.000	1.82
Ticrr	1.23	5.25	12.38	0.00	0.000	10.17
Fbxl15	1.23	2.39	7.61	0.00	0.000	4.67
Rnf34	1.23	4.66	11.24	0.00	0.000	9.01
Entpd5	1.23	3.44	5.51	0.00	0.000	1.13
Abhd11	1.23	4.48	10.64	0.00	0.000	8.36
Kntc1	1.23	5.55	10.67	0.00	0.000	8.26
Baz1b	1.24	7.47	13.49	0.00	0.000	11.16
Hcfc1	1.24	7.57	10.42	0.00	0.000	7.84
Tubb4b	1.24	8.55	13.98	0.00	0.000	11.62
Gm12919	1.24	0.24	4.26	0.00	0.002	-0.59
Gm12854	1.24	1.03	4.50	0.00	0.001	-0.29
Gm7665	1.24	2.73	6.58	0.00	0.000	3.02
Rbm27	1.24	5.89	10.19	0.00	0.000	7.65
Nbn	1.25	4.74	10.48	0.00	0.000	8.14
1700008J07Rik	1.25	0.59	4.67	0.00	0.001	0.08
Coq5	1.25	5.43	11.65	0.00	0.000	9.38
3010003L21Rik	1.25	1.87	5.04	0.00	0.001	0.50
Rfc2	1.25	5.75	13.03	0.00	0.000	10.80
Gm15850	1.25	1.10	3.47	0.00	0.008	-2.21
Otud6b	1.25	5.42	9.28	0.00	0.000	6.54
Gm5864	1.25	0.31	3.86	0.00	0.004	-1.37
Fbxl14	1.25	6.14	14.24	0.00	0.000	11.92
Ap1ar	1.25	5.79	12.42	0.00	0.000	10.18
Gm20604	1.26	1.02	4.54	0.00	0.001	-0.23
Fgf21	1.26	1.32	4.52	0.00	0.001	-0.36
Prrg2	1.26	2.87	6.27	0.00	0.000	2.45
Pcgf6	1.26	6.01	13.68	0.00	0.000	11.41
Slc25a5	1.26	7.91	15.62	0.00	0.000	13.08
Gm15387	1.26	0.78	4.71	0.00	0.001	0.15
Lbh	1.26	1.67	5.87	0.00	0.000	2.08
Zfp664	1.26	6.00	13.05	0.00	0.000	10.80
Rai14	1.26	5.98	11.74	0.00	0.000	9.44
AV356131	1.26	1.01	4.41	0.00	0.002	-0.38
Ssx2ip	1.27	5.01	12.44	0.00	0.000	10.26
Rpa1	1.27	6.83	13.53	0.00	0.000	11.22
Aven	1.27	4.37	10.00	0.00	0.000	7.62
Iba57	1.27	2.16	6.22	0.00	0.000	2.48
Ap4s1	1.27	3.01	8.65	0.00	0.000	6.05
Ccne2	1.27	4.72	9.35	0.00	0.000	6.73
Hmgn2	1.27	5.78	11.44	0.00	0.000	9.13
Gm10156	1.27	1.21	4.88	0.00	0.001	0.42
Adrm1	1.27	7.18	9.87	0.00	0.000	7.19
Il4i1	1.27	1.52	4.73	0.00	0.001	0.08

Nup155	1.27	6.25	13.71	0.00	0.000	11.42
Pole	1.27	6.14	14.28	0.00	0.000	11.96
Rrm1	1.27	7.83	13.53	0.00	0.000	11.19
Reep6	1.28	8.01	11.47	0.00	0.000	9.06
C030034I22Rik	1.28	2.02	5.11	0.00	0.001	0.72
Ldha	1.28	10.41	13.51	0.00	0.000	11.19
Traf3ip1	1.28	3.92	8.58	0.00	0.000	5.81
Phf19	1.28	5.53	12.50	0.00	0.000	10.28
Gm17018	1.28	3.15	8.54	0.00	0.000	5.90
Brca1	1.28	4.72	9.58	0.00	0.000	7.02
Prkcz	1.28	0.10	3.84	0.00	0.004	-1.38
Hspa12a	1.28	3.11	9.77	0.00	0.000	7.38
Gm14117	1.29	0.44	4.01	0.00	0.003	-1.10
Gclm	1.29	5.80	10.67	0.00	0.000	8.24
Rnf145	1.29	6.89	15.67	0.00	0.000	13.14
5033406O09Rik	1.29	0.13	4.30	0.00	0.002	-0.52
Eif3b	1.29	9.15	13.02	0.00	0.000	10.70
Hmgb1-ps2	1.29	0.94	5.03	0.00	0.001	0.72
Hsp90aa1	1.29	9.56	13.13	0.00	0.000	10.81
AI661453	1.29	4.05	10.09	0.00	0.000	7.73
Adamts1	1.29	4.52	10.94	0.00	0.000	8.74
Tonsl	1.29	4.44	8.22	0.00	0.000	5.20
AC122861.1	1.29	2.15	6.47	0.00	0.000	2.98
Nasp	1.29	7.27	14.19	0.00	0.000	11.83
Topbp1	1.29	7.07	14.47	0.00	0.000	12.09
Ints2	1.29	4.11	8.22	0.00	0.000	5.30
Crybg1	1.29	5.38	12.82	0.00	0.000	10.62
Gm12435	1.30	2.70	7.90	0.00	0.000	5.06
Avil	1.30	5.55	10.04	0.00	0.000	7.61
Dsn1	1.30	4.31	10.57	0.00	0.000	8.29
Nbl1	1.30	3.95	7.92	0.00	0.000	4.89
Dyrk1b	1.30	0.29	4.07	0.00	0.003	-0.98
Bend3	1.30	5.18	11.07	0.00	0.000	8.76
Ggact	1.30	3.43	7.63	0.00	0.000	4.54
Rnf227	1.30	2.71	8.03	0.00	0.000	5.24
Gm48583	1.30	1.10	4.80	0.00	0.001	0.28
N4bp2	1.30	1.69	4.84	0.00	0.001	0.21
Ubxn8	1.31	3.08	7.65	0.00	0.000	4.65
Dhcr24	1.31	5.86	13.24	0.00	0.000	10.99
Fanci	1.31	3.82	10.28	0.00	0.000	8.01
Azin1	1.31	7.05	11.92	0.00	0.000	9.57
Rrp12	1.31	5.76	10.82	0.00	0.000	8.42
Prps1l3	1.31	5.14	9.01	0.00	0.000	6.21
Gbe1	1.32	0.91	3.32	0.01	0.010	-2.44
Otud3	1.32	3.48	7.37	0.00	0.000	4.15
Psph	1.32	6.62	15.13	0.00	0.000	12.70
Gm7125	1.32	−1.13	3.79	0.00	0.005	-1.43
Knl1	1.33	5.40	7.86	0.00	0.000	4.58
Gm7931	1.33	0.33	4.23	0.00	0.002	-0.66
Gm4285	1.33	0.13	3.14	0.01	0.014	-2.70
Id2	1.33	3.74	6.77	0.00	0.000	3.17

Chek1	1.33	4.95	10.83	0.00	0.000	8.53
Gm42715	1.33	−1.10	3.68	0.00	0.005	-1.66
2200002D01Rik	1.33	3.86	9.53	0.00	0.000	7.09
Prpf3	1.33	6.69	11.45	0.00	0.000	9.08
Rgs19	1.33	3.77	9.98	0.00	0.000	7.66
Chaf1a	1.33	6.71	12.43	0.00	0.000	10.12
Slc7a11	1.33	3.91	8.15	0.00	0.000	5.21
Sgms2	1.34	4.74	9.48	0.00	0.000	6.89
AC168306.1	1.34	0.83	4.87	0.00	0.001	0.42
Chtf18	1.34	4.96	11.08	0.00	0.000	8.81
Snhg10	1.34	−1.12	2.99	0.01	0.018	-2.88
Haus5	1.34	0.97	4.30	0.00	0.002	-0.61
Gm12346	1.34	5.39	12.51	0.00	0.000	10.31
Hsph1	1.35	7.48	13.20	0.00	0.000	10.88
Tpi-rs11	1.35	1.23	5.02	0.00	0.001	0.64
Cdon	1.35	3.30	7.57	0.00	0.000	4.47
Slc29a4	1.35	−1.33	3.38	0.00	0.009	-2.28
3110083C13Rik	1.35	1.75	5.06	0.00	0.001	0.64
Gm5487	1.35	1.93	5.53	0.00	0.000	1.43
Rps6ka1	1.36	4.18	11.74	0.00	0.000	9.61
Cdc45	1.36	4.81	9.72	0.00	0.000	7.19
Uchl3	1.36	5.63	13.39	0.00	0.000	11.17
Hmgxb4	1.36	3.86	11.53	0.00	0.000	9.44
Ide	1.36	6.29	13.20	0.00	0.000	10.93
Tarbp2	1.36	4.20	10.45	0.00	0.000	8.17
Gbx2	1.36	1.03	4.83	0.00	0.001	0.35
Tubb4b-ps1	1.36	2.95	8.63	0.00	0.000	6.03
Rffl	1.37	2.10	6.78	0.00	0.000	3.47
Rhpn2	1.37	4.32	11.77	0.00	0.000	9.65
Sars2	1.37	3.56	7.46	0.00	0.000	4.29
Relt	1.37	3.32	9.43	0.00	0.000	7.07
Gm11175	1.37	2.72	6.24	0.00	0.000	2.48
Gm9774	1.37	3.35	7.75	0.00	0.000	4.74
Gm3325	1.37	2.07	4.31	0.00	0.002	-0.82
Dock4	1.37	3.49	6.48	0.00	0.000	2.70
Gm9761	1.38	1.94	7.57	0.00	0.000	4.67
Ddias	1.38	3.12	8.17	0.00	0.000	5.40
Mcm4	1.38	6.24	12.30	0.00	0.000	10.03
Cdca5	1.38	5.29	12.52	0.00	0.000	10.32
Sh3tc1	1.38	1.59	7.08	0.00	0.000	3.96
Pom121	1.38	7.11	17.06	0.00	0.000	14.26
Vps33a	1.38	5.49	11.97	0.00	0.000	9.73
Bicdl1	1.38	2.84	8.16	0.00	0.000	5.40
Sptb	1.38	1.18	4.52	0.00	0.001	-0.15
Llgl2	1.39	3.01	8.74	0.00	0.000	6.15
Coa7	1.39	3.99	10.08	0.00	0.000	7.75
Mad1l1	1.39	5.71	11.43	0.00	0.000	9.12
Mcm7	1.39	7.41	14.08	0.00	0.000	11.73
Esco2	1.40	4.62	13.85	0.00	0.000	11.69
Gm7446	1.40	1.72	6.08	0.00	0.000	2.39
Ing5	1.40	4.73	10.50	0.00	0.000	8.16

Padi4	1.40	0.70	5.06	0.00	0.001	0.72
Dhx37	1.40	5.77	12.85	0.00	0.000	10.61
Gm5529	1.40	0.62	4.52	0.00	0.001	-0.16
Sart3	1.40	6.07	12.70	0.00	0.000	10.45
Bms1	1.40	6.98	16.33	0.00	0.000	13.69
Parvg	1.40	0.10	4.28	0.00	0.002	-0.51
Sfpq	1.40	7.47	12.91	0.00	0.000	10.59
Tnfrsf23	1.41	3.67	10.58	0.00	0.000	8.39
Wdr76	1.41	3.68	10.16	0.00	0.000	7.89
Sept11	1.41	7.57	12.49	0.00	0.000	10.16
Lbhd1	1.41	1.64	6.30	0.00	0.000	2.75
Mthfsl	1.41	0.09	3.99	0.00	0.003	-1.04
Ect2	1.41	6.41	10.78	0.00	0.000	8.33
Uchl4	1.41	0.75	4.98	0.00	0.001	0.64
Ppip5k2	1.42	6.17	12.15	0.00	0.000	9.87
Pcdh10	1.42	1.21	5.16	0.00	0.000	0.88
Gm9144	1.42	0.21	4.29	0.00	0.002	-0.56
2410131K14Rik	1.42	4.81	11.38	0.00	0.000	9.15
Lrrc14b	1.42	−1.37	3.92	0.00	0.004	-1.24
Chml	1.42	4.31	8.13	0.00	0.000	5.12
Noc4l	1.42	5.92	11.91	0.00	0.000	9.63
BC085271	1.43	1.22	6.15	0.00	0.000	2.60
Gm10282	1.43	4.29	12.23	0.00	0.000	10.15
Slc4a11	1.43	4.38	12.12	0.00	0.000	10.00
Pgam5	1.43	6.02	16.72	0.00	0.000	14.04
Mirt1	1.43	0.82	4.12	0.00	0.003	-0.92
Zgrf1	1.44	3.10	8.23	0.00	0.000	5.48
Gm5452	1.44	0.61	5.12	0.00	0.001	0.92
Ubash3b	1.44	3.75	11.30	0.00	0.000	9.21
Lrr1	1.44	2.59	9.12	0.00	0.000	6.73
Gm5847	1.45	0.26	4.40	0.00	0.002	-0.31
Phlda2	1.45	5.13	13.28	0.00	0.000	11.10
Gm6750	1.45	0.28	4.87	0.00	0.001	0.51
Lactb	1.45	4.83	14.05	0.00	0.000	11.86
Rpa2	1.46	5.18	13.00	0.00	0.000	10.82
Cdsn	1.47	5.60	11.61	0.00	0.000	9.41
Gusb	1.47	5.89	16.77	0.00	0.000	14.09
AC135509.1	1.47	−1.10	4.54	0.00	0.001	-0.04
Cry1	1.47	4.49	12.77	0.00	0.000	10.68
Kif24	1.47	2.75	6.48	0.00	0.000	2.88
Gm5844	1.48	3.41	10.12	0.00	0.000	7.91
Clspn	1.48	5.26	13.39	0.00	0.000	11.19
Gm29093	1.48	0.39	4.74	0.00	0.001	0.26
Sssca1	1.48	1.34	5.20	0.00	0.000	0.94
Gm48284	1.48	0.30	4.11	0.00	0.003	-0.91
Acyp2	1.48	1.00	3.42	0.00	0.008	-2.25
Gm11814	1.49	0.26	4.96	0.00	0.001	0.69
BC005561	1.49	1.96	4.03	0.00	0.003	-1.33
Galnt3	1.49	4.09	11.96	0.00	0.000	9.88
Cpsf2	1.49	6.03	14.34	0.00	0.000	12.03
N6amt1	1.49	3.95	11.25	0.00	0.000	9.14

Lmln	1.50	2.63	8.57	0.00	0.000	6.00
4921531C22Rik	1.50	0.09	4.21	0.00	0.002	-0.67
Dnajc9	1.50	5.05	15.93	0.00	0.000	13.47
Tes	1.50	6.64	18.71	0.00	0.000	15.50
Cars2	1.50	4.00	11.50	0.00	0.000	9.40
Mapkapk5	1.50	−1.07	3.76	0.00	0.005	-1.46
Mgst3	1.50	2.99	9.26	0.00	0.000	6.84
Lrrc59	1.51	7.85	16.70	0.00	0.000	13.97
Ly6a	1.51	0.48	4.00	0.00	0.003	-1.16
Siah1b	1.51	2.23	7.14	0.00	0.000	4.00
Tns3	1.51	3.92	12.55	0.00	0.000	10.50
Rtl10	1.51	−1.11	4.82	0.00	0.001	0.46
Pappa2	1.52	−1.05	2.97	0.01	0.018	-2.92
Anapc5	1.52	7.64	17.34	0.00	0.000	14.47
Eif5	1.52	8.06	13.33	0.00	0.000	11.00
Apobec3	1.52	2.03	7.75	0.00	0.000	4.85
Melk	1.52	5.25	14.32	0.00	0.000	12.07
AC133523.4	1.52	0.39	4.35	0.00	0.002	-0.44
Zik1	1.52	2.35	6.51	0.00	0.000	3.00
Snai2	1.52	1.48	5.33	0.00	0.000	1.21
Ppa1	1.52	7.31	16.14	0.00	0.000	13.53
Sh3tc2	1.53	3.96	11.92	0.00	0.000	9.87
Slc25a10	1.53	5.20	10.90	0.00	0.000	8.57
Fut11	1.53	0.69	5.47	0.00	0.000	1.51
Ankrd13a	1.53	6.98	17.61	0.00	0.000	14.68
Hmga2	1.53	6.96	16.84	0.00	0.000	14.09
Hist1h1c	1.53	3.26	9.16	0.00	0.000	6.72
Tyms	1.54	4.77	12.39	0.00	0.000	10.26
Nudt15	1.54	0.54	6.04	0.00	0.000	2.48
Ccdc86	1.55	5.59	10.87	0.00	0.000	8.49
Pdss1	1.55	4.06	12.24	0.00	0.000	10.19
Rwdd2b	1.56	2.26	8.78	0.00	0.000	6.33
Tmem79	1.56	1.47	5.87	0.00	0.000	2.09
Pnp2	1.56	1.10	6.78	0.00	0.000	3.58
Wdhd1	1.56	5.36	15.53	0.00	0.000	13.12
Serpinb9b	1.56	5.93	17.59	0.00	0.000	14.73
Dusp3	1.56	5.97	14.87	0.00	0.000	12.52
Adm2	1.57	5.59	11.02	0.00	0.000	8.66
Prph	1.57	−1.30	3.43	0.00	0.008	-2.09
Fam241a	1.57	3.18	8.91	0.00	0.000	6.41
Dnaaf5	1.57	5.33	16.38	0.00	0.000	13.82
Itgb7	1.57	4.89	12.26	0.00	0.000	10.17
Zfp568	1.58	4.85	10.81	0.00	0.000	8.50
Hist1h3d	1.58	0.21	4.04	0.00	0.003	-1.01
2700038G22Rik	1.58	1.99	6.38	0.00	0.000	2.88
Ncbp2	1.58	4.58	14.12	0.00	0.000	11.94
Dhx9	1.58	7.00	13.67	0.00	0.000	11.35
E2f2	1.59	4.76	11.66	0.00	0.000	9.48
Gnptab	1.59	7.09	13.42	0.00	0.000	11.11
Cenpu	1.59	4.22	14.18	0.00	0.000	12.03
Slc25a22	1.59	4.78	13.51	0.00	0.000	11.36

Cpeb2	1.60	3.77	7.17	0.00	0.000	3.79
Dis3	1.60	5.72	14.24	0.00	0.000	11.96
Pfkfb1	1.60	1.18	6.08	0.00	0.000	2.47
Smg5	1.60	7.37	15.55	0.00	0.000	13.03
Bpifb4	1.61	0.08	4.24	0.00	0.002	-0.62
Pclaf	1.61	4.96	15.25	0.00	0.000	12.91
Ldha-ps2	1.61	1.64	7.47	0.00	0.000	4.60
B230377A18Rik	1.61	−1.07	3.87	0.00	0.004	-1.30
Ckb	1.61	2.98	10.81	0.00	0.000	8.74
Fignl1	1.62	4.45	14.24	0.00	0.000	12.07
6430548M08Rik	1.62	4.46	12.66	0.00	0.000	10.53
Gm36266	1.62	−1.28	3.01	0.01	0.017	-2.81
Jup	1.62	6.40	14.10	0.00	0.000	11.76
Epn3	1.62	3.05	10.16	0.00	0.000	7.97
Tgfb2	1.63	0.67	5.57	0.00	0.000	1.69
Pop1	1.63	4.87	12.13	0.00	0.000	9.98
Gm9247	1.63	1.50	7.01	0.00	0.000	3.90
Cass4	1.63	0.56	3.91	0.00	0.004	-1.25
Zfp985	1.64	4.04	12.57	0.00	0.000	10.51
Dtx1	1.64	0.05	4.89	0.00	0.001	0.51
Aldh1a3	1.64	0.12	4.72	0.00	0.001	0.28
Arhgef28	1.64	1.93	8.03	0.00	0.000	5.35
Nagpa	1.65	4.02	12.36	0.00	0.000	10.32
Mns1	1.66	3.08	8.70	0.00	0.000	6.15
Taf7	1.66	3.29	12.84	0.00	0.000	10.84
Gm37691	1.67	0.00	4.57	0.00	0.001	0.04
Isoc1	1.67	2.99	10.40	0.00	0.000	8.30
Lck	1.67	2.69	8.90	0.00	0.000	6.46
Hvcn1	1.68	1.28	6.06	0.00	0.000	2.44
Nphp3	1.68	0.72	3.96	0.00	0.003	-1.20
Gm14124	1.68	−1.27	4.06	0.00	0.003	-0.94
Thg1l	1.68	3.53	9.76	0.00	0.000	7.46
Lrrc8b	1.69	4.59	12.73	0.00	0.000	10.62
Ptgfrn	1.69	8.92	17.66	0.00	0.000	14.70
Pnma3	1.69	0.30	5.40	0.00	0.000	1.43
Krt8-ps	1.69	3.98	13.07	0.00	0.000	11.02
Lmnb1	1.69	7.14	17.82	0.00	0.000	14.84
Fam161b	1.69	−1.19	4.24	0.00	0.002	-0.58
Sdr42e1	1.70	−1.17	3.25	0.01	0.011	-2.33
Ptgis	1.70	0.78	5.14	0.00	0.001	0.87
Orc1	1.70	3.35	12.11	0.00	0.000	10.12
Gm10357	1.70	−1.16	4.19	0.00	0.002	-0.69
Zfp473	1.70	2.43	7.74	0.00	0.000	4.84
Ssc4d	1.71	3.34	10.90	0.00	0.000	8.80
Gpatch3	1.71	2.59	8.87	0.00	0.000	6.40
Ap5b1	1.71	3.36	10.90	0.00	0.000	8.78
Ciart	1.71	5.43	16.94	0.00	0.000	14.26
Gm37855	1.72	0.86	6.37	0.00	0.000	2.96
Exo1	1.72	5.11	16.06	0.00	0.000	13.57
Eme1	1.72	2.86	9.61	0.00	0.000	7.34
Krt8	1.72	9.41	15.91	0.00	0.000	13.33

Ptpn18	1.72	−1.86	3.56	0.00	0.007	-1.90
Serpinb9	1.73	3.36	11.96	0.00	0.000	9.97
Trim36	1.73	1.04	6.48	0.00	0.000	3.15
Wars2	1.74	3.11	7.83	0.00	0.000	4.93
Gm10516	1.74	1.37	7.41	0.00	0.000	4.52
Ly9	1.74	1.59	7.60	0.00	0.000	4.78
Fen1	1.75	5.60	14.15	0.00	0.000	11.87
Rbm19	1.75	5.59	13.88	0.00	0.000	11.64
Polq	1.75	3.82	10.93	0.00	0.000	8.80
4930503E14Rik	1.76	−1.14	4.57	0.00	0.001	0.04
Gm4430	1.76	0.53	5.87	0.00	0.000	2.16
Pfkfb2	1.77	0.68	4.01	0.00	0.003	-1.13
Uhrf1	1.77	7.35	18.11	0.00	0.000	15.06
Fhdc1	1.77	3.66	13.80	0.00	0.000	11.72
Gmfg	1.77	1.45	8.56	0.00	0.000	6.10
Tedc1	1.78	4.24	16.29	0.00	0.000	13.80
Utp14b	1.78	4.14	9.73	0.00	0.000	7.30
Dna2	1.78	2.93	9.76	0.00	0.000	7.53
Gm14276	1.79	1.47	7.77	0.00	0.000	5.03
Slamf9	1.79	0.43	5.07	0.00	0.001	0.89
Brpf3	1.79	3.90	13.23	0.00	0.000	11.18
Stx1a	1.80	0.61	4.73	0.00	0.001	0.20
Ocln	1.80	0.55	6.26	0.00	0.000	2.83
Foxa1	1.80	2.35	9.47	0.00	0.000	7.21
Tcam1	1.80	3.17	12.71	0.00	0.000	10.74
Ift122	1.81	4.55	16.22	0.00	0.000	13.73
Zfp978	1.82	0.34	3.82	0.00	0.004	-1.45
Pls1	1.82	0.26	4.85	0.00	0.001	0.50
Hells	1.82	6.01	16.03	0.00	0.000	13.50
BC039966	1.82	−1.28	5.50	0.00	0.000	1.61
Gm44335	1.83	0.33	6.10	0.00	0.000	2.59
Gmpr	1.83	−1.57	4.56	0.00	0.001	-0.08
1700034H15Rik	1.83	−1.05	5.58	0.00	0.000	1.74
Ctf1	1.83	1.48	7.96	0.00	0.000	5.30
Ints1	1.83	6.17	16.20	0.00	0.000	13.63
Gm5446	1.83	0.55	6.09	0.00	0.000	2.56
Mphosph9	1.84	4.33	12.87	0.00	0.000	10.81
Mcm5	1.84	6.91	15.57	0.00	0.000	13.07
Gm9958	1.84	0.33	6.10	0.00	0.000	2.60
Trim45	1.85	4.64	11.47	0.00	0.000	9.26
Rad51	1.87	5.66	18.34	0.00	0.000	15.27
Man1a2	1.87	7.44	21.18	0.00	0.000	17.13
Fbxo48	1.87	−1.09	4.05	0.00	0.003	-0.93
E2f7	1.88	4.41	15.50	0.00	0.000	13.16
Hist1h3f	1.88	−1.13	5.91	0.00	0.000	2.31
Ksr2	1.89	4.72	13.89	0.00	0.000	11.73
Fbxo2	1.89	1.69	6.73	0.00	0.000	3.37
Ccne1	1.89	4.16	13.88	0.00	0.000	11.77
Mboat1	1.89	1.92	9.13	0.00	0.000	6.83
Sh3yl1	1.90	0.59	5.27	0.00	0.000	1.17
Gm3320	1.90	1.65	7.10	0.00	0.000	4.05

Angptl4	1.91	4.08	13.49	0.00	0.000	11.43
Kbtbd8	1.92	2.77	9.28	0.00	0.000	6.94
Mybl2	1.92	6.44	19.95	0.00	0.000	16.36
Gm6970	1.92	−1.91	3.47	0.00	0.008	-2.03
Acox2	1.92	2.14	7.51	0.00	0.000	4.64
Gcnt1	1.94	4.31	14.63	0.00	0.000	12.42
Homer1	1.95	3.01	11.02	0.00	0.000	8.99
Lin54	1.95	4.72	12.09	0.00	0.000	9.95
Tfcp2l1	1.95	1.40	7.19	0.00	0.000	4.23
Hsd11b2	1.96	−1.01	5.26	0.00	0.000	1.22
Srrt	1.97	6.80	24.53	0.00	0.000	19.07
Fcer2a	1.97	1.03	8.72	0.00	0.000	6.28
6330549D23Rik	1.97	0.73	6.45	0.00	0.000	3.12
Nudt1	1.97	2.71	8.36	0.00	0.000	5.76
D130058E05Rik	1.97	0.30	5.29	0.00	0.000	1.27
AC113104.4	1.97	−1.19	4.98	0.00	0.001	0.74
1110006O24Rik	1.99	−1.11	4.16	0.00	0.002	-0.72
Matn4	1.99	0.77	7.00	0.00	0.000	3.92
Dpep1	2.00	1.63	9.60	0.00	0.000	7.41
Haspin	2.00	3.86	11.77	0.00	0.000	9.70
Usp2	2.02	2.76	12.58	0.00	0.000	10.63
Sbsn	2.03	3.05	11.19	0.00	0.000	9.17
Cbx2	2.04	−1.01	4.76	0.00	0.001	0.34
Clip4	2.04	1.68	9.55	0.00	0.000	7.36
Gm2381	2.04	1.42	5.85	0.00	0.000	2.07
Sdr39u1	2.04	4.60	15.41	0.00	0.000	13.07
Sema4d	2.05	3.08	13.84	0.00	0.000	11.79
Slco2a1	2.05	3.39	15.18	0.00	0.000	12.94
Slc22a3	2.05	1.07	7.83	0.00	0.000	5.14
Cyth4	2.06	2.00	8.51	0.00	0.000	6.00
Ptpn3	2.06	−1.08	6.05	0.00	0.000	2.50
B130055M24Rik	2.08	0.91	6.46	0.00	0.000	3.12
Kcnh1	2.09	0.08	4.91	0.00	0.001	0.62
Dhrs4	2.09	2.15	10.73	0.00	0.000	8.71
Cd38	2.10	3.08	12.38	0.00	0.000	10.42
Dnmt3l	2.10	1.82	8.15	0.00	0.000	5.57
Krt80	2.10	3.06	11.17	0.00	0.000	9.17
Plekhh1	2.10	3.66	13.99	0.00	0.000	11.90
Rfc5	2.11	5.42	18.02	0.00	0.000	15.06
4931428F04Rik	2.11	3.05	10.41	0.00	0.000	8.32
Fhad1	2.11	−1.09	6.70	0.00	0.000	3.52
Alpl	2.12	1.07	6.94	0.00	0.000	3.78
Amhr2	2.13	0.83	7.50	0.00	0.000	4.70
Chrnb1	2.14	4.15	16.86	0.00	0.000	14.26
Gm17399	2.15	0.28	5.59	0.00	0.000	1.76
Dck	2.16	3.75	15.50	0.00	0.000	13.20
Ccdc38	2.16	0.03	6.63	0.00	0.000	3.42
Cdc6	2.17	3.87	14.21	0.00	0.000	12.09
Ptpn6	2.17	2.20	11.24	0.00	0.000	9.26
Gm5648	2.17	0.73	8.01	0.00	0.000	5.40
Fam83a	2.18	1.02	8.14	0.00	0.000	5.56

Dscc1	2.18	2.53	9.87	0.00	0.000	7.71
Rmi2	2.19	3.53	14.68	0.00	0.000	12.52
Kcnq1	2.19	−1.45	5.54	0.00	0.000	1.68
4930461G14Rik	2.19	1.00	10.20	0.00	0.000	8.11
Cdkl2	2.19	3.80	14.39	0.00	0.000	12.26
Gm47813	2.22	0.90	7.43	0.00	0.000	4.59
Gpat2	2.22	−1.66	5.65	0.00	0.000	1.84
Tmco4	2.24	4.28	18.43	0.00	0.000	15.39
2310040G24Rik	2.27	0.99	8.98	0.00	0.000	6.66
Scel	2.27	1.07	7.39	0.00	0.000	4.50
Ccdc120	2.30	0.75	7.51	0.00	0.000	4.71
Samd10	2.30	2.21	10.62	0.00	0.000	8.59
Gm35823	2.32	0.95	7.57	0.00	0.000	4.78
Grhl2	2.33	0.30	7.05	0.00	0.000	4.05
Rhof	2.34	0.49	7.85	0.00	0.000	5.16
Bcar3	2.36	2.29	12.98	0.00	0.000	10.99
Syne4	2.36	0.58	7.23	0.00	0.000	4.29
Elovl3	2.40	2.12	9.70	0.00	0.000	7.53
Zfp784	2.40	−1.42	6.13	0.00	0.000	2.66
Cdh24	2.41	0.36	6.06	0.00	0.000	2.52
Mbnl3	2.42	2.37	8.33	0.00	0.000	5.73
Sema4f	2.42	−1.09	5.14	0.00	0.001	1.04
Fnbp1l	2.43	3.63	16.84	0.00	0.000	14.24
Sh3bp4	2.43	1.92	10.30	0.00	0.000	8.24
Slc52a3	2.45	2.08	12.94	0.00	0.000	10.95
Rasgrp3	2.48	−1.39	4.71	0.00	0.001	0.31
Tex101	2.48	3.32	12.11	0.00	0.000	10.12
Tigit	2.49	3.63	18.46	0.00	0.000	15.39
Trim66	2.50	0.49	7.39	0.00	0.000	4.54
Omp	2.51	−1.09	5.83	0.00	0.000	2.17
Cyp4f40	2.52	0.91	7.72	0.00	0.000	4.99
Traf1	2.54	2.28	10.35	0.00	0.000	8.28
Tmem62	2.54	0.97	7.56	0.00	0.000	4.77
Prss32	2.56	−1.20	8.27	0.00	0.000	5.70
Mat1a	2.59	0.40	7.18	0.00	0.000	4.23
Ccdc169	2.60	−1.17	6.42	0.00	0.000	3.10
Sspo	2.61	2.27	7.56	0.00	0.000	4.75
Cd74	2.62	1.49	10.19	0.00	0.000	8.08
Gm38416	2.63	−1.38	6.24	0.00	0.000	2.83
Nfatc2	2.64	1.17	9.55	0.00	0.000	7.36
Tapbpl	2.67	4.00	18.42	0.00	0.000	15.38
Plek2	2.67	2.69	13.30	0.00	0.000	11.31
Aqp3	2.68	−1.83	6.15	0.00	0.000	2.69
Igf2bp1	2.69	2.60	14.88	0.00	0.000	12.64
Nedd9	2.70	0.68	8.86	0.00	0.000	6.49
Car6	2.71	6.44	24.47	0.00	0.000	19.03
Lcn2	2.73	1.61	8.18	0.00	0.000	5.62
Atp2a3	2.73	2.88	15.54	0.00	0.000	13.15
Zfp335os	2.76	0.12	6.52	0.00	0.000	3.26
Ccdc88c	2.78	1.96	12.17	0.00	0.000	10.21
Gata4	2.79	1.50	6.79	0.00	0.000	3.58

Elf3	2.80	2.37	15.06	0.00	0.000	12.79
Fam83h	2.85	1.61	11.87	0.00	0.000	9.91
Tmem116	2.88	0.94	8.94	0.00	0.000	6.61
Dusp8	2.89	3.69	19.17	0.00	0.000	15.80
Otog	2.90	1.37	7.54	0.00	0.000	4.74
Gm10762	2.93	−1.03	6.62	0.00	0.000	3.41
Fmnl1	2.94	−1.58	6.80	0.00	0.000	3.66
Sorl1	3.00	1.16	10.29	0.00	0.000	8.18
Tmem238	3.03	4.54	14.86	0.00	0.000	12.64
Hspb7	3.05	−1.66	6.12	0.00	0.000	2.65
Vmn2r3	3.05	−1.78	4.23	0.00	0.002	-0.46
Rab17	3.05	0.28	7.21	0.00	0.000	4.24
Cnksr1	3.08	3.04	21.79	0.00	0.000	17.32
Esrp2	3.09	−1.45	7.28	0.00	0.000	4.37
Dennd2d	3.12	0.79	8.50	0.00	0.000	6.02
Ccdc116	3.14	0.49	11.77	0.00	0.000	9.59
Padi1	3.15	−1.91	8.89	0.00	0.000	6.42
Tfrc	3.15	5.55	24.14	0.00	0.000	18.86
Ostn	3.16	−1.31	3.73	0.00	0.005	-1.32
5830416I19Rik	3.18	−1.22	8.00	0.00	0.000	5.32
AU018091	3.19	−1.14	7.34	0.00	0.000	4.39
Rph3al	3.19	0.21	11.58	0.00	0.000	9.35
Mamdc2	3.19	2.68	14.05	0.00	0.000	11.81
Itgal	3.20	1.86	13.51	0.00	0.000	11.47
Bmp7	3.22	3.42	19.63	0.00	0.000	16.09
Trpv3	3.26	−1.12	7.19	0.00	0.000	4.24
Gm17184	3.26	−1.55	6.82	0.00	0.000	3.68
Ccdc92	3.29	2.70	16.59	0.00	0.000	14.00
Rbmx	3.34	3.13	15.38	0.00	0.000	13.05
Gm26692	3.35	−1.52	5.40	0.00	0.000	1.50
Serpinb9c	3.39	0.11	9.43	0.00	0.000	7.11
Emb	3.40	2.10	15.90	0.00	0.000	13.34
Tmem102	3.44	−1.03	7.71	0.00	0.000	4.87
Aox3	3.44	−1.66	5.01	0.00	0.001	0.86
Sult4a1	3.44	0.36	9.36	0.00	0.000	7.01
Plet1	3.47	1.49	13.69	0.00	0.000	11.43
Stx3	3.48	3.48	21.06	0.00	0.000	16.90
Gm3336	3.60	−1.91	6.27	0.00	0.000	2.88
Tns4	3.65	−1.34	8.88	0.00	0.000	6.41
Spry3	3.69	−1.14	5.59	0.00	0.000	1.80
Sptbn2	3.77	2.91	20.19	0.00	0.000	16.33
Tmem40	3.83	−1.85	6.55	0.00	0.000	3.30
Prkg2	3.88	0.03	8.37	0.00	0.000	5.77
Ak7	3.93	−1.26	10.46	0.00	0.000	8.08
Utp20	3.97	7.83	31.68	0.00	0.000	22.37
Zdhhc23	3.97	0.49	10.42	0.00	0.000	8.12
Ovol1	4.00	2.44	16.18	0.00	0.000	13.52
Pkp3	4.01	3.86	24.75	0.00	0.000	18.96
Mylk	4.06	2.16	16.56	0.00	0.000	13.77
Creb3l3	4.07	−1.62	9.79	0.00	0.000	7.29
Clec2f	4.10	−1.11	9.42	0.00	0.000	7.02

Crabp2	4.20	−1.88	8.02	0.00	0.000	5.24
Fcgbp	4.36	0.17	13.23	0.00	0.000	10.57
Frmd4b	4.36	−1.11	8.85	0.00	0.000	6.07
Dpf1	4.39	1.01	6.78	0.00	0.000	3.65
Sbk3	4.44	1.58	16.52	0.00	0.000	13.62
Taf7l	4.46	0.13	9.14	0.00	0.000	6.39
Acsbg1	4.49	−1.89	5.78	0.00	0.000	2.05
Gm4316	4.62	1.63	13.37	0.00	0.000	11.15
Lncenc1	4.66	1.90	12.35	0.00	0.000	10.36
Kcnma1	4.69	−1.71	4.95	0.00	0.001	0.74
Lamb3	4.70	1.51	19.19	0.00	0.000	14.91
Spdye4b	4.85	−1.03	10.41	0.00	0.000	7.63
Hgfac	5.00	−1.40	6.33	0.00	0.000	2.87
Ano9	5.01	−1.01	5.63	0.00	0.000	1.83
Plekha7	5.03	−1.92	10.05	0.00	0.000	7.37
Prss22	5.04	−1.22	6.26	0.00	0.000	2.75
Stra6	5.12	0.38	9.58	0.00	0.000	7.06
Arhgap27	5.28	−1.75	10.27	0.00	0.000	7.55
Pou5f1	5.42	−1.28	7.40	0.00	0.000	4.28
Scd3	5.44	−1.04	13.17	0.00	0.000	10.11
Fam167a	5.44	−1.38	11.93	0.00	0.000	9.10
Gjb4	5.54	−1.98	7.36	0.00	0.000	4.24
Cyp4f39	5.68	−1.99	8.95	0.00	0.000	6.08
Slc18a1	5.81	−1.53	8.24	0.00	0.000	5.27
Tmem144	5.95	−1.78	7.05	0.00	0.000	3.84
Tmem266	6.18	−1.56	7.95	0.00	0.000	5.02
Gm2a	6.38	−1.76	8.24	0.00	0.000	5.27
Gm5640	6.39	−1.65	8.34	0.00	0.000	5.39
Eya2	6.69	−1.24	12.41	0.00	0.000	9.26
Mapk8ip2	6.70	−1.47	10.36	0.00	0.000	7.42
Gm5936	6.87	−1.07	11.72	0.00	0.000	8.38
Ptgs1	7.04	−1.14	14.82	0.00	0.000	10.39
Blnk	7.19	−1.67	9.07	0.00	0.000	6.22
Gpa33	7.58	−1.79	13.92	0.00	0.000	9.87
Il24	7.70	−1.35	15.47	0.00	0.000	10.53
Sncg	7.72	−1.86	13.62	0.00	0.000	9.69
Lpo	7.87	−1.87	16.43	0.00	0.000	10.97
Fgfbp1	7.92	−1.36	17.68	0.00	0.000	11.46
Krt14	7.94	−1.35	17.85	0.00	0.000	11.52
Bicd1	7.97	−1.33	15.14	0.00	0.000	10.43
Vrtn	8.09	−1.30	16.37	0.00	0.000	10.98
Nxf7	8.35	−1.51	15.09	0.00	0.000	10.51
Krt18	8.38	−1.30	15.93	0.00	0.000	10.97
Dmkn	8.40	−1.83	15.68	0.00	0.000	10.73
Lypd5	8.52	−1.84	16.56	0.00	0.000	11.12
Inhbe	8.93	−1.85	11.39	0.00	0.000	8.32
Lypd3	9.87	−1.05	18.34	0.00	0.000	11.96
Slc6a13	9.97	−1.19	20.74	0.00	0.000	12.69

Table S4. Alternatively spliced genes in TNBC cells following miR-200c restoration.

#	Gene Symbol	*p*-Value	RefSeq	Gene Gene
1	KIF1B	2.71E-153	NM_015074	Kinesin family member 1B
2	MACF1	5.39E-146	NM_012090	Microtubule-actin crosslinking factor 1
3	DST	1.52E-141	NM_001723	Dystonin
4	UBB	1.61E-109	NM_018955	Ubiquitin B
5	DDR1	9.28E-103	NM_001202521	Discoidin domain receptor tyrosine kinase 1
6	ANK2	1.29E-98	NM_001127493	Ankyrin 2
7	NPNT	1.32E-93	NM_001033047	Nephronectin
8	GPR126	1.10E-87	NM_001032394	G-protein coupled receptor 126
9	LRBA	2.88E-87	NM_001199282	LPS responsive beige-like anchor protein
10	LPHN2	2.15E-73	NM_012302	Latrophilin 2
11	MARK3	3.27E-71	NM_001128918	Microtubule affinity regulating kinase 3
12	ESYT2	1.93E-68	NM_020728	Extended synaptotagmin 2
13	ITGB4	1.76E-61	NM_000213	Integrin subunit beta 4
14	PLCB4	1.79E-61	NM_000933	Phospholipase c beta 4
15	TPM1	3.80E-61	NM_000366	Tropomyosin 1
16	LAMA5	3.31E-56	NM_005560	Laminin subunit alpha 5
17	FHOD3	4.67E-54	NM_025135	Formin homology 2 domain containing 3
18	OSBPL6	4.99E-52	NM_001201480	Oxysterol binding protein like 6
19	LAMC2	8.60E-52	NM_005562	Laminin subunit alpha 5
20	NIN	1.49E-49	NM_016350	Ninein
21	KIF21A	2.67E-49	NM_001173463	Kinesin family member 21A
22	ERBB3	3.16E-47	NM_001005915	Erb-B2 receptor tyrosine kinase 3
23	TPM3	6.35E-47	NM_001043351	Tropomyosin 3
24	VEPH1	6.80E-46	NM_001167911	Ventricular zone expressed PH domain containing 1
25	ZEB1	2.87E-45	NM_001128128	Zinc finger E-box binding homeobox 1
26	ZNF385B	3.15E-45	NM_001113397	Zinc finger protein 385B
27	VAV3	1.60E-43	NM_001079874	Vav guanine nucleotide exchange factor 3
28	EPB41L4B	1.67E-43	NM_018424	Erythrocyte membrane protein band 4.1 like 4B
29	CCSER2	7.63E-43	NM_018999	Coiled-coil serine rich protein 2
30	NRXN3	8.47E-43	NM_001105250	Neurexin 3
31	RAI14	1.04E-42	NM_001145520	Retinoic acid induced 14
32	PCNXL2	1.26E-42	NM_014801	Pecanex homolog 2
33	FAT1	4.50E-42	NM_005245	FAT atypical cadherin 1
34	OSBPL8	6.69E-42	NM_001003712	Oxysterol binding protein like 8
35	SEPT11	1.79E-41	NM_018243	Septin 11
36	SYTL2	3.81E-41	NM_001162951	Synaptotagmin like 2
37	NALCN	2.55E-40	NM_052867	Sodium leak channel, non-selective
38	SLC12A8	2.86E-40	NM_001195483	Solute carrier family 12 member 8
39	ATP5C1	5.24E-38	NM_001001973	ATP synthase F1 subunit gamma
40	AP1S2	6.78E-38	NM_003916	Adaptor related protein complex 1 sigma 2 subunit
41	PLEKHA1	3.38E-37	NM_001001974	Pleckstrin homology domain containing A1
42	MST1R	4.96E-37	NM_001244937	Macrophage stimulating 1 receptor
43	TPD52L1	9.24E-37	NM_001003395	Tumor protein D52 like 1
44	CDH3	1.50E-36	NM_001793	Cadherin 3
45	MAP7	4.14E-36	NM_001198608	Microtubule associated protein 7
46	STXBP5	7.54E-36	NM_001127715	Syntaxin binding protein 5
47	CASK	1.67E-35	NM_001126054	Calcium/calmodulin dependent serine protein kinase
48	PLEKHA5	4.72E-35	NM_001143821	Pleckstrin homology domain containing A5
49	TIAF1	2.99E-34	NM_004740	TGFB1-induced anti-apoptotic factor 1
50	PRKAR1B	4.36E-33	NM_001164758	Protein kinase CAMP-dependent type I regulatory subunit beta
51	SPAG9	6.84E-33	NM_001130527	Sperm associated antigen 9
52	CHODL	7.93E-33	NM_001204174	Chondrolectin
53	CLSTN1	9.01E-33	NM_001009566	Calsyntenin 1

54	ANK3	9.24E-33	NM_001149	Ankyrin 3
55	STX2	9.41E-33	NM_001980	Syntaxin 2
56	OGDH	9.76E-33	NM_001003941	Oxoglutarate dehydrogenase
57	JAG2	1.85E-32	NM_002226	Jagged 2
58	DEPDC1	3.01E-31	NM_001114120	DEP domain containing 1
59	MFSD6	6.83E-31	NM_017694	Major facilitator superfamily domain containing 6
60	GOLGA4	1.10E-30	NM_001172713	Golgin A4
61	SLC29A2	4.12E-29	NM_001532	Solute carrier family 29 member 2
62	CKMT1A	9.23E-29	NM_001015001	Creatine kinase, mitochondrial 1A
63	ERBB2IP	1.04E-28	NM_001006600	Erbb2 interacting protein
64	ABCC3	1.30E-28	NM_001144070	ATP binding cassette subfamily c member 3
65	H2AFY	7.10E-28	NM_001040158	H2A histone family member Y
66	FAM13C	7.99E-28	NM_001001971	Family with sequence similarity 13 member C
67	SMPDL3B	3.46E-27	NM_001009568	Sphingomyelin phosphodiesterase acid like 3B
68	SLAIN1	1.19E-26	NM_001040153	SLAIN motif family member 1
69	UPK3B	1.30E-26	NM_030570	Uroplakin 3B
70	PHKA2	7.33E-26	NM_000292	Phosphorylase kinase regulatory subunit alpha 2
71	SLC44A3	1.14E-25	NM_001114106	Solute carrier family 44 member 3
72	CAST	5.10E-25	NM_001042440	Calpastatin
73	TPD52	6.44E-25	NM_001025252	Tumor protein D52
74	LRRFIP1	1.27E-24	NM_001137550	LRR binding FLII interacting protein 1
75	DLG1	1.50E-24	NM_001098424	Discs large MAGUK scaffold protein 1
76	MPRIP	1.84E-24	NM_015134	Myosin phosphatase Rho interacting protein
77	CNTN4	2.01E-24	NM_001206955	Contactin 4
78	AP1S3	2.94E-24	NM_001039569	Adaptor related protein complex 1 sigma 3 subunit
79	SEPT6	4.25E-24	NM_015129	Septin 6
80	EPCAM	2.74E-23	NM_002354	Epithelial cell adhesion molecule
81	MARC1	3.46E-23	NM_022746	Mitochondrial amidoxine reducting component 1
82	TMEM25	7.30E-23	NM_001144034	Transmembrane protein 25
83	DCAF6	1.27E-22	NM_001017977	DDB1 and CUL4 associated factor 6
84	GRB7	1.50E-22	NM_001030002	Growth factor receptor bound protein 7
85	WDFY3	1.66E-22	NM_014991	WD repeat and FYVE domain containing 3
86	NHSL1	1.70E-22	NM_001144060	NHS like 1
87	EPB41	2.17E-22	NM_001166005	Erythrocyte membrane protein band 4.1
88	GSAP	2.96E-22	NM_017439	Gamma-secretase activating protein
89	CENPT	3.34E-22	NM_025082	Centromere protein T
90	DTNB	4.35E-22	NM_001256303	Dystrobrevin beta
91	MBP	4.96E-22	NM_001025081	Myelin basic protein
92	VGLL4	7.12E-22	NM_001128219	Vestigial like family member 4
93	STX3	9.09E-22	NM_001178040	Syntaxin 3
94	SPINT2	1.94E-21	NM_001166103	Serine peptidase inhibitor, Kunitz type 2
95	STK10	2.32E-21	NM_005990	Serine/threonine kinase 10
96	NUMB	3.13E-21	NM_001005743	NUMB, endocytic adaptor protein
97	RAB11FIP4	3.21E-21	NM_032932	RAB11 famility interacting protein 4
98	WDR96	6.97E-21	NM_025145	WD repeat-containing protein 96
99	SVIL	1.62E-20	NM_003174	Supervillin
100	FOXP1	3.80E-20	NM_001012505	Forkhead box P1

Includes only the top 100 alternatively spliced genes identified by microarray and visually verified by two independent observers; TNBC, triple-negative breast cancer.

Table S5. Alternatively spliced genes in TNBC cells following miR-200c restoration.

#	Gene Symbol	*p*-value	RefSeq	Gene gene
1	KIF1B	2.71E-153	NM_015074	Kinesin family member 1B
2	MACF1	5.39E-146	NM_012090	Microtubule-actin crosslinking factor 1
3	DST	1.52E-141	NM_001723	Dystonin
4	UBB	1.61E-109	NM_018955	Ubiquitin B

5	DDR1	9.28E-103	NM_001202521	Discoidin domain receptor tyrosine kinase 1
6	ANK2	1.29E-98	NM_001127493	Ankyrin 2
9	LRBA	2.88E-87	NM_001199282	LPS responsive beige-like anchor protein
11	MARK3	3.27E-71	NM_001128918	Microtubule affinity regulating kinase 3
12	ESYT2	1.93E-68	NM_020728	Extended synaptotagmin 2
13	ITGB4	1.76E-61	NM_000213	Integrin subunit beta 4
15	TPM1	3.80E-61	NM_000366	Tropomyosin 1
16	LAMA5	3.31E-56	NM_005560	Laminin subunit alpha 5
17	FHOD3	4.67E-54	NM_025135	Formin homology 2 domain containing 3
19	LAMC2	8.60E-52	NM_005562	Laminin subunit alpha 5
20	NIN	1.49E-49	NM_016350	Ninein
23	TPM3	6.35E-47	NM_001043351	Tropomyosin 3
25	ZEB1	2.87E-45	NM_001128128	Zinc finger E-box binding homeobox 1
29	CCSER2	7.63E-43	NM_018999	Coiled-coil serine rich protein 2
31	RAI14	1.04E-42	NM_001145520	Retinoic acid induced 14
33	FAT1	4.50E-42	NM_005245	FAT atypical cadherin 1
34	OSBPL8	6.69E-42	NM_001003712	Oxysterol binding protein like 8
39	ATP5C1	5.24E-38	NM_001001973	ATP synthase F1 subunit gamma
40	AP1S2	6.78E-38	NM_003916	Adaptor related protein complex 1 sigma 2 subunit
41	PLEKHA1	3.38E-37	NM_001001974	Pleckstrin homology domain containing A1
42	MST1R	4.96E-37	NM_001244937	Macrophage stimulating 1 receptor
46	STXBP5	7.54E-36	NM_001127715	Syntaxin binding protein 5
47	CASK	1.67E-35	NM_001126054	Calcium/calmodulin dependent serine protein kinase
48	PLEKHA5	4.72E-35	NM_001143821	Pleckstrin homology domain containing A5
51	SPAG9	6.84E-33	NM_001130527	Sperm associated antigen 9
53	CLSTN1	9.01E-33	NM_001009566	Calsyntenin 1
54	ANK3	9.24E-33	NM_001149	Ankyrin 3
55	STX2	9.41E-33	NM_001980	Syntaxin 2
56	OGDH	9.76E-33	NM_001003941	Oxoglutarate dehydrogenase
58	DEPDC1	3.01E-31	NM_001114120	DEP domain containing 1
60	GOLGA4	1.10E-30	NM_001172713	Golgin A4
61	SLC29A2	4.12E-29	NM_001532	Solute carrier family 29 member 2
64	ABCC3	1.30E-28	NM_001144070	ATP binding cassette subfamily c member 3
67	SMPDL3B	3.46E-27	NM_001009568	Sphingomyelin phosphodiesterase acid like 3B
70	PHKA2	7.33E-26	NM_000292	Phosphorylase kinase regulatory subunit alpha 2
71	SLC44A3	1.14E-25	NM_001114106	Solute carrier family 44 member 3
72	CAST	5.10E-25	NM_001042440	Calpastatin
73	TPD52	6.44E-25	NM_001025252	Tumor protein D52
74	LRRFIP1	1.27E-24	NM_001137550	LRR binding FLII interacting protein 1
75	DLG1	1.50E-24	NM_001098424	Discs large MAGUK scaffold protein 1
76	MPRIP	1.84E-24	NM_015134	Myosin phosphatase Rho interacting protein
80	EPCAM	2.74E-23	NM_002354	Epithelial cell adhesion molecule
82	TMEM25	7.30E-23	NM_001144034	Transmembrane protein 25
83	DCAF6	1.27E-22	NM_001017977	DDB1 and CUL4 associated factor 6
85	WDFY3	1.66E-22	NM_014991	WD repeat and FYVE domain containing 3
86	NHSL1	1.70E-22	NM_001144060	NHS like 1
87	EPB41	2.17E-22	NM_001166005	Erythrocyte membrane protein band 4.1
89	CENPT	3.34E-22	NM_025082	Centromere protein T
90	DTNB	4.35E-22	NM_001256303	Dystrobrevin beta
92	VGLL4	7.12E-22	NM_001128219	Vestigial like family member 4

93	STX3	9.09E-22	NM_001178040	Syntaxin 3
94	SPINT2	1.94E-21	NM_001166103	Serine peptidase inhibitor, Kunitz type 2
95	STK10	2.32E-21	NM_005990	Serine/threonine kinase 10
96	NUMB	3.13E-21	NM_001005743	NUMB, endocytic adaptor protein
99	SVIL	1.62E-20	NM_003174	Supervillin
100	FOXP1	3.80E-20	NM_001012505	Forkhead box P1
63	ERBB2IP	1.04E-28	NM_001006600	Erbb2 interacting protein
65	H2AFY	7.10E-28	NM_001040158	H2A histone family member Y
35	SEPT11	1.79E-41	NM_018243	Septin 11
79	SEPT6	4.25E-24	NM_015129	Septin 6

Includes only the top 100 alternatively spliced genes identified by microarray and visually verified by two independent observers; TNBC, triple-negative breast cancer.
